# Development and Implementation of a Family Presence Facilitator Curriculum for Interprofessional Use in Pediatric Medical Resuscitations

**DOI:** 10.15766/mep_2374-8265.11445

**Published:** 2024-10-08

**Authors:** Ellen Duncan, Joanne Agnant, Kymme Napoli, Selin T. Sagalowsky

**Affiliations:** 1 Assistant Professor, Departments of Emergency Medicine and Pediatrics, New York University Langone Health; 2 Clinical Associate Professor, Departments of Emergency Medicine and Pediatrics, New York University Langone Health; 3 Child Life Specialist, Department of Child Life and Developmental Services, Bellevue Hospital Center; 4 Associate Professor, Departments of Emergency Medicine and Pediatrics, New York University Langone Health

**Keywords:** Patient-Centered Care, Family-Centered Care, Simulation, Simulated Participants, Emergency Medicine, Focus Groups/Interviews

## Abstract

**Introduction:**

Family presence during pediatric medical resuscitation has myriad benefits. However, there is significant heterogeneity in provider acceptance and implementation of the family support role. We designed this curriculum to teach all members of the health care team best practices in the Family Presence Facilitator (FPF) role during pediatric medical resuscitations.

**Methods:**

We applied Kern's six-step approach to develop an FPF curriculum comprising didactic and interactive elements, along with training for simulated participants. We implemented the curriculum through (a) live sessions (30-minute didactic or 90-minute workshop) for learners; (b) a 20-minute asynchronous version of the didactic curriculum for self-directed learning; and (c) a 1-hour, monthly, in situ simulation curriculum in a pediatric emergency department setting. Curriculum evaluation surveys queried self-reported engagement, satisfaction, relevance, confidence, commitment, knowledge, skills, and attitudes in a retrospective pre/post format.

**Results:**

We collected data from 153 learners, including attendings, fellows, residents, advanced practice providers, medical students, and child life specialists, between October 2022 and September 2023. Only 22% of participants had received similar prior training. One hundred percent of learners found the curriculum enjoyable and engaging; learners also agreed the curriculum improved their knowledge and skills in providing empathetic and respectful communication (99%); nonspeculative, clear information (100%); and nonverbal support (99%). Of respondents, 100% believed the curriculum would improve the patient care experience.

**Discussion:**

Facilitating family presence during pediatric medical resuscitations is a crucial skill. Our curriculum improves self-reported confidence, knowledge, and skills among interprofessional learners. Next steps include expanding this curriculum beyond the pediatric setting.

## Educational Objectives

By the end of this training, learners will be able to:

1. Prelearning/didactics: Articulate the purpose and benefits of the Family Presence Facilitator (FPF) role.
2. Prelearning/didactics: List the four components of the FPF role and key behaviors for each.
3. If participating in simulated participant training: Demonstrate the key personality types in a role-play scenario.
4. If participating in a simulated component: Demonstrate behaviors of the FPF Simulation Assessment Tool (FPF-SAT; i.e., respect and value, information-sharing, nonverbal communication, summary and follow-up) in a simulated resuscitation.

## Introduction

Pediatric medical resuscitations are low-frequency, high-stakes events that require the coordination of many factors, including emergent medical decision-making, technical skills, interprofessional teamwork, and communication with patients and families. Family presence during pediatric resuscitations is increasingly the standard of care, and benefits include allowing family members (including parents, caregivers, and/or loved ones) to comfort and advocate for patients, decreasing emotional trauma, and easing families’ grief in cases of patient death.^1–4^ Both the ACGME and the Joint Commission on Accreditation of Healthcare Organizations cite communication with patients and families as a core competency,^2,5^ supporting the premise that this is also a prescribed and normative need in health professions education.^6^ Moreover, there is substantial evidence that family presence during resuscitations does not interfere with, but rather benefits, patient care.^7–9^ Although families wish to be offered a supported presence, there are significant differences in how family presence during pediatric resuscitations is perceived by health care providers, with acceptance ranging from 35% to 85%, without significant differences between physicians and nurses.^10^ Despite data showing that health care providers are more comfortable facilitating family presence during invasive procedures and resuscitations after implementing specific practice guidelines,^11^ guidance and evidence are limited on how best to teach or evaluate team members providing emotional support to patients and families.

Facilitating family presence during resuscitations requires training, refinement, and thoughtful evaluation of our practices. For optimal impact, training in complex skills like patient and family communication during critical events should include both didactic and experiential components and, when possible, allow for practice in the team's resuscitation space.^12^ Interprofessional, in situ simulation provides an ideal setting in which to develop and teach such skills, as it enables learners to practice with authentic teams, resources, culture, leadership, and implementation climates. Structured debriefing, such as that described in the Promoting Excellence and Reflective Learning in Simulation (PEARLS) debriefing framework,^13^ allows for discussion of crucial learning objectives from a variety of fields. Indeed, the simulation literature suggests that practicing simulation in a so-called blended model—in which the pursuit of medical and technical learning objectives is accompanied by a focus on interprofessional teamwork, situational awareness, and patient- and family-centered communication—heightens emotional arousal and psychological fidelity, thereby enhancing learner engagement and retention of targeted knowledge and skills.^14,15^ However, creating such curricula in a manner that promotes psychological fidelity and experiential learning is resource intensive. Therefore, it is critical to create curricular materials that can be adapted and implemented across a variety of educational settings.

In this Educational Summary Report (ESR), we describe the development, implementation, and evaluation of a curriculum to teach and promote the role of Family Presence Facilitator (FPF) during pediatric resuscitations and provide resources for clinician-educators to implement this curriculum for interprofessional learners. This curriculum was developed based on generalized and targeted needs assessments, including (a) a comprehensive literature review; (b) an appraisal of communication domains from existing patient communication tools^6,16–20^; and (c) focus groups with families and youth who have had the experience of being patients and/or family members in our health care system.^21^ We developed and used a modified Delphi process to refine a novel FPF Simulation Assessment Tool (FPF-SAT) in parallel to curriculum development. The FPF curriculum was revised in an iterative fashion, applying feedback from learners and FPF-SAT development. Our curriculum was designed with a broad and interprofessional audience in mind, including physicians (attendings, fellows, residents), medical students, nurses, advanced practice providers (APPs), child life specialists, chaplains, and social workers. The overall goal of the FPF curriculum is to empower all individuals on the health care team to provide psychosocial support to patients and families, and to improve humanistic patient- and family-centered communication during pediatric medical resuscitations.

## Methods

### Development

#### Curriculum design

We used Kern's six-step approach,^22^ consisting of problem identification, general and targeted needs assessments, formulation of goals and objectives, creation of educational strategies, implementation of the curriculum, and evaluation and feedback from learners, to develop our curriculum. A literature review of existing curricula within medicine and allied professions demonstrated a paucity of structured curricula for the FPF role.^11^ Our local needs assessment identified existing resources, including a brief guide for communicating with patients and families during resuscitations, previously developed by members of our team.^23^ Additionally, our institution benefits from support and partnership with The Sala Institute for Child and Family Centered Care (i.e., “The Sala Institute”), a dedicated institute whose mission is to design, promote, and fund vital services in child and family support, resilience, safety, quality, and family partnership at NYU Langone Health/Hassenfeld Children's Hospital. The targeted needs assessment included key stakeholder meetings with health care providers and focus groups with The Sala Institute's family and youth advisory councils to identify behavioral domains essential to the FPF role. Family advisory council members consisted of parents of critically ill children and youth advisory council members composed of teenage patients who had experienced acute illness. We conducted two focus groups comprising six members each; because of the COVID-19 pandemic, focus groups were conducted using a virtual platform. We queried participants regarding key domains adapted from The Sala Institute's Patient and Family Faculty Program's Patient and Family Centered Competency Scorecard, which is a checklist previously developed to assess health care professionals in key patient- and family-centered communication skills.^21^ Domains comprised in this scorecard include role clarity (e.g., how patients and family members would like to be addressed and introduced), information-sharing practices, choice and autonomy (e.g., values and preferences, including deescalation techniques), culturally sensitive communication, nonverbal communication, and communication with conscious patients.

These results were applied to determine curricular goals, objectives, and methodology. Our focus group discussions were not originally designed to address communication and management of patient death. While creating our curriculum, we noticed that most models of delivering bad news and communicating patient death assume that families are not present at the time of patient death. As such, based on iterative learner feedback, we modified our curriculum to provide recommendations in cases of patient death in the family's presence, recognizing that guidelines for detailed summary communication between physicians and families are beyond the scope of this curriculum.

Our final curriculum is multimodal, with teaching materials of various lengths and formats that can be adapted to the learning needs, resources, time constraints, and preferences across various educational settings. Specifically, we created one set of materials designed to be delivered in didactic/workshop format, including (a) a 30-minute PowerPoint didactic to be delivered by an instructor (Appendix A), along with a prerecorded version of the PowerPoint for self-directed learning (Appendix B) and (b) a 90-minute workshop that pairs the didactic with an interactive session based on a role-play demonstration performed by instructors and learners in the group (scripts in Appendices C and D; worksheets in Appendices E and F). All learners were asked to complete a posttraining survey (Appendix G) after partaking in the workshop. No prerequisite knowledge was required to complete the curriculum.

Additionally, we created materials that facilitated experiential teaching and learning of the FPF role using interprofessional, in situ simulation as a curricular framework. We encouraged learners to experience the PowerPoint (as a didactic, workshop, or self-directed learning) prior to participating in simulation-based practice and learning the FPF skills. Because simulated participants (SPs) were crucial for the simulation-based portion of our curriculum, we also developed a 90-minute SP curriculum to train SPs to portray family members during simulated pediatric resuscitations. A PowerPoint (Appendix H) and simulation scenario (Appendix I) for SP training were created in collaboration with the New York Simulation Center for the Health Sciences (NYSIM). The SP training introduced the FPF curriculum and described three core personality types (agitated, anxious, and quiet) to be portrayed by SPs. We also briefed SPs regarding their emotional starting points for the cases (on a scale of 1–10) and included sample conditional statements to adjust behavioral intensity based on the performance of the FPF. SP didactic instruction was supplemented with SP role-play and feedback, and SPs were trained to complete our novel assessment tool to rate learners’ performance in these domains (FPF-SAT; Appendix J). Training sessions were led by authors Ellen Duncan and Selin Sagalowsky in conjunction with NYSIM's SP Program Educator. Our SPs were recruited with help from the SP Program Educator as well, but SPs participating in this training do not need any prerequisite knowledge prior to the training.

Both the FPF curriculum and the SP training curriculum began with a content advisory and provided resources regarding trauma-informed care. We recognize the sensitive nature of the curricula and endeavored to create an environment of psychological safety for all participants.

#### Equipment/environment

The 30-minute, standalone didactic and SP training required only PowerPoint capabilities and audiovisual (AV) equipment (i.e., a computer and a projector). The 90-minute expanded workshop required PowerPoint and AV capabilities, along with simulation equipment (if desired) to carry out the two role-play demonstrations performed by the educator(s). These demonstrations were designed to be low fidelity and adaptable to different settings based on available resources; basic needs included a table, a low-fidelity infant manikin (though even a simple infant-sized doll can be used with mimicking of procedures) and, if available, airway equipment (including an infant bag-valve-mask, endotracheal tube, and laryngoscope blade and handle), IV equipment (with sharps removed for safety), two 3–5cc syringes to simulate medications, such as antibiotics, and a small blanket to use as a shoulder roll.

Our implementation of the didactic curriculum was accompanied by a longitudinal, in situ simulation curriculum for a subset of learners. However, we recognize that this learning modality may not be available at all institutions and have thus developed a multimodal curriculum that does not rely on this simulation-based education component. For institutions that do have the resources for recurring in situ simulations, we recommend that either an SP or member of the team portray the family member role. Because the focus of this report is to discuss the FPF curriculum and how it may be generalized across practice settings, the specific needs for the in situ curriculum will not be elaborated upon.

#### Personnel

The 30-minute didactic and SP training curriculum may both be taught by a single educator. The expanded workshop required at least five participants for the role-play demonstration: team leader, airway provider, primary nurse, parent, and FPF. Any of these roles may be played by the primary educator.

The in situ simulation was more labor intensive and required interprofessional participation. Programs with an existing in situ curriculum may integrate the FPF curriculum into their existing simulation by employing an SP or team member to portray the family member and ensuring that there is a team member who can fill the FPF role.

### Implementation

#### Curriculum delivery

We trained providers from different professions in the FPF role using the brief didactic, including institutionally (division of pediatric emergency medicine, emergency medicine residency training program, pediatric residency training program, and neonatal intensive care fellowship training program) and nationally at the 2023 Society for Academic Emergency Medicine conference. The didactic was presented via a computer and projector, as detailed above. We also conducted the longer workshop institutionally for the third-year medical student class as part of the existing Integrated Clinical Skills course curriculum and nationally at the 2023 Pediatric Academic Societies conference. For the workshop, we utilized a computer and projector to present the curriculum. We then ran the first role-play demonstration (FPF Role-Play Script Without FPF, Appendix C) using the equipment listed above and subsequently divided learners into small groups (four to five members per group) to discuss and complete the accompanying participant and instructor worksheets (Appendices E and F). The first scenario (without the FPF) is intended to facilitate active engagement such that learners could identify the performance gap and brainstorm when and how the FPF could provide support to the family member during the worksheet portion of the workshop. After the large-group report-out, we ran the second role-play demonstration (Appendix D), followed by small-group discussion and large-group report-out. This allowed learners to contrast the two scenarios and witness the ideas they generated during the worksheet portion in action during the second resuscitation scenario.

Didactic material from this workshop (Appendix B) was uploaded to our institution's educational website and sent to our physician, nursing, chaplaincy, and social work colleagues via hospital-wide listservs for asynchronous viewing.

#### SP recruitment and training

To conduct the simulation portion of this curriculum, we trained four SPs in performing the FPF role, using the curriculum described below and provided in Appendices H and I. The SP training for the FPF role was approximately 1 hour in length, comprising PowerPoint didactics and simulated role-play. We recruited SPs with the help of the NYSIM SP Program Educator from a dedicated group of SPs who worked with our institution's simulation center. Due to limitations associated with the COVID-19 pandemic, we conducted our SP training virtually for a group of four SPs; however, training may be conducted in person if feasible and/or desired.

In our SP training, we first presented the SP Training PowerPoint (Appendix H), which summarized the purpose of the curriculum and the three main personality types. SPs then read through the SP Training Script (Appendix I) and took turns role-playing those personality types. We also introduced the SPs to the FPF-SAT and together practiced completing the assessment tool to ensure that SPs felt comfortable with the checklist. This assessment tool was developed using existing assessment tools in OSCE work that were employed by our simulation center and with which our SPs had great familiarity. In this regard, we were fortunate that our SPs were very comfortable in the role of assessing learners. However, the SP training curriculum was designed to introduce SPs to the tool so that even those who have not used such a rating before are comfortable doing so.

We conducted only one training session per group and provided detailed SP notes prior to each in situ simulation. All SP training sessions included a content advisory about the sensitive nature of these cases, the importance of psychological safety, and reiteration of the voluntary nature of SP participation. We also included a slide on the importance of deroling to encourage SPs to separate themselves from the person they portrayed in the simulation.

#### In situ simulation

Interprofessional members of our division of pediatric emergency medicine experienced the simulation-based component of FPF training as part of a longitudinal curriculum. In situ simulation sessions were conducted monthly in our pediatric emergency department and were led by a PEM faculty/fellow dyad. Simulation participants included PEM attending physicians and fellows; pediatric and emergency medicine residents; pediatric and general emergency medicine nurses; pharmacists and pharmacy residents; respiratory therapists; and, where applicable, consultant physicians (including teams from the neonatal ICU, neurosurgery, and obstetrics and gynecology). Each simulation followed a standardized format, with a 5-minute prebrief, 10-minute simulation, and 40-minute debrief. Each prebrief began with a statement about the importance of psychological safety in this learning environment.

Starting in September 2022, after the initial delivery of our FPF didactics and workshops, we began integrating SPs into our simulations to portray the family member. In each simulation, the resuscitation team leader was responsible for assigning an FPF to communicate with and provide support to the patient and/or family. In the debrief, the facilitator discussed not only medical and teamwork domains but also patient- and family-centered communication.

### Debriefing

Portions of the curriculum that employed simulation-based instruction were debriefed. In the interactive workshop, both the small-group worksheet discussion and large-group report-out served as a mechanism for workshop participants to engage in focused facilitation with group leaders to reflect on the witnessed role-play demonstration. Our in situ simulation program comprised a 10-minute simulation, followed by a 40-minute scripted debriefing that utilized the PEARLS debriefing format^13^ and used a combination of direct feedback, Plus/Delta, and focused facilitation methodologies.

### Assessment

We conducted a retrospective pre/post assessment using the Kirkpatrick New World model of program evaluation^24^ to query self-reported reactions and learning to evaluate the didactic curriculum, using 4-point Likert-like scales to dichotomize results (Appendix G). The retrospective pre/post design allowed for data collection from a broad audience, mitigated response-shift bias, and had been demonstrated to be effective and comparable to traditional pre/post self-assessments in health professions education. The same survey tool was used, irrespective of what version of the curriculum learners received (i.e., 30-minute didactic, 90-minute workshop, or asynchronous curriculum), with an option for learners who completed the experiential workshop to provide additional qualitative feedback. Due to the heterogeneous settings in which this curriculum was delivered, we did not record the total number of people who attended all sessions; however, an estimated <5% of participants declined to complete the evaluation. We did not separately assess learner evaluation of the longitudinal in situ simulation curriculum.

The FPF-SAT (Appendix J) was developed by the authors to provide feedback to the medical team on key behaviors identified in the FPF curriculum. After each simulation, the SP completed the FPF-SAT to evaluate FPF. The SP used their completed FPF-SAT to guide verbal feedback during the debrief, and the completed tool was also provided to the FPF and medical team as written formative feedback. These data, along with qualitative feedback, were incorporated into revisions of the curriculum and assessment tool.

### Data Collection and Analysis

Data were collected via the Qualtrics platform. Posttraining survey results for the FPF curriculum were dichotomized to compare agreement (*agree*/*strongly agree*) with disagreement (*disagree*/*strongly disagree*), using chi-squared testing (IBM SPSS Statistics version 29).

## Results

An estimated 160 learners completed the curriculum, of which 153 learners (Table 1) responded to the curriculum evaluation. Of these respondents, 26% experienced the brief didactic session; 67% experienced the expanded workshop; and 7% experienced the longitudinal simulation curriculum. None of the respondents experienced the asynchronous curriculum only. Results of the evaluation are shown in Table 2, with strong agreement ranging from 68% to 83% for each question. A demonstrative sample of qualitative feedback is provided in Table 3.

**Table 1.**
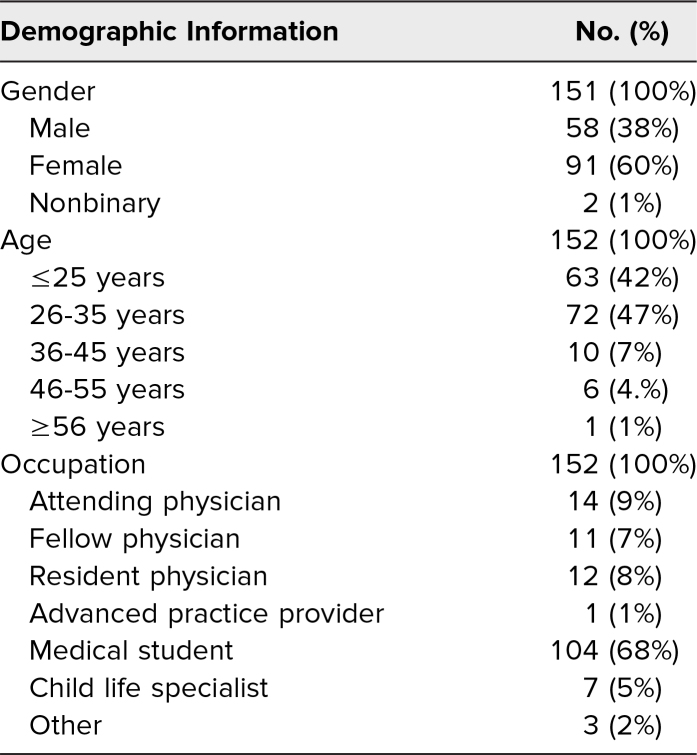
Demographic Characteristics of Learners Participating in Family Presence Facilitator Curriculum

**Table 2.**
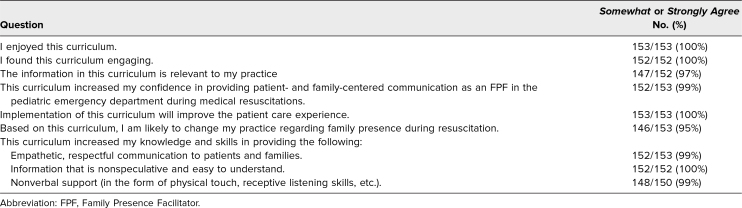
Survey Results From Learners Who Participated in the FPF Curriculum (Brief Didactic, Workshop, and/or Asynchronous Components)

**Table 3.**
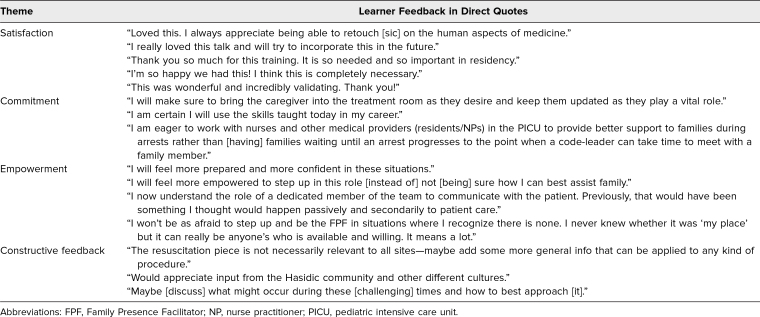
Qualitative Comments From Learners Who Participated in the FPF Curriculum (Brief Didactic, Workshop, and/or Asynchronous Components)

Overall, posttraining surveys indicated that this curriculum was enjoyable, engaging, and relevant, with 95% to 100% of respondents reporting agreement (*agree*/*strongly agree*) with curriculum evaluation outcomes (*p* < .001 across all domains). Although patient-oriented outcomes are the gold standard of medical education scholarship, our curriculum used proxy measures because pediatric resuscitations are rare and emotionally sensitive events. Notably, only 33/152 (22% vs. 78%, *p* < .001) of participants had received similar prior training. The workshop component of the training was particularly well received (100% of respondents reported it to be useful), with one participant noting, “The hands-on component was the most useful portion of the whole experience.” Given the highly skewed nature of our responses, we did not separate self-reported results by learning modality.

In contrast to the majority of respondents, seven of 153 (5%) reported they would not change their practice based on this curriculum. Of these respondents, four of seven (57%) were attending physicians, two of seven (29%) were medical students, and one of seven (14%) were advanced practice providers. Of respondents, five of 152 (3%) reported that the curriculum was not relevant to their practice; these respondents comprised four of the five (80%) medical students and, interestingly, one of the five (20%) were child life specialists.

Nevertheless, all these respondents reported the curriculum to be enjoyable, engaging, and resulting in improved knowledge and skills. One of these learners commented: “I will be more aware of designating someone to fill that role, I think it is important.” Constructive feedback suggested expanding the curriculum beyond resuscitation; including perspectives from a broader range of religions, ethnicities, and cultures; and highlighting the challenges of the FPF role.

Results from the FPF-SAT, as rated by SPs in our in situ simulations (Table 4), showed that, on average, FPFs performed well (>50% were rated *well done*) on nine of 15 behaviors. Feedback on tailoring of information quantity was more variable: although 53% of SPs felt that this was well done, 33% reported that information quantity was not tailored to their preferences and needs. Qualitative feedback highlighted the importance of providing culturally competent care with appropriate language interpretation services (“There was an immediate disconnect the moment they found out the parent only spoke Spanish. This caused a lot of distress throughout the encounter, until the translator was ‘brought’”) as well as nonverbal communication (“The lack of eye contact or knowledge of myself made me feel extremely desperate and clueless, even before they realized I didn't speak English. I don't remember more than 1 team member looking into my eyes to try to understand me and help me.”) These data from the FPF-SAT represented a small number due to the resource-intensive nature of simulation, especially that with an SP component; however, we found the results were valuable with respect to general feedback as well as specific, domain-centered feedback. The FPF-SAT rated participant performance in the FPF role, in contrast to the posttraining survey, which evaluated learner self-reported reactions and knowledge acquisition. Because both tools were anonymous, we were not able to perform a retrospective analysis to evaluate whether self-reported learning outcomes correlated with objective performance in the FPF role.

**Table 4.**
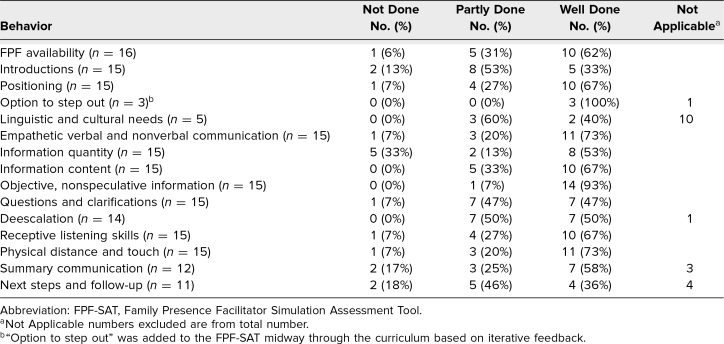
Results From Simulated Participants on the FPF-SAT

## Discussion

Facilitating family presence during pediatric resuscitations has myriad benefits and is an essential skill for interprofessional teams that provide care to pediatric patients. A prior study found that implementation of practice guidelines and training increased provider comfort with family presence during both invasive procedures and resuscitations.^11^ However, little research has been conducted to determine best practices for facilitating family presence during acute resuscitations, a crucial component of patient- and family-centered care that requires sensitive, dedicated training.

In this ESR, we described the development, implementation, and evaluation of a curriculum designed to instruct all members of the health care team on how to provide this care. Through a literature review, appraisal of existing resources, and focus groups, we identified four main categories of behavior: respect and value; information-sharing; nonverbal communication; and summary and follow-up. Our curriculum provides essential training in applying these behaviors to provide emotional support to patients and families, in various formats adaptable to the needs of learners and educators (i.e., brief didactic; expanded workshop; asynchronous curriculum; and longitudinal simulation-based experiential learning).

Our results indicate that the curriculum is enjoyable, engaging, and relevant. Dissenting results (i.e., those who did not find the curriculum relevant or think it would change their practice) may be explained in part by the narrower scope of pediatric emergency department-based resuscitations on which we focused, and on participants having had prior similar training in curricular components. These results were also balanced with learners concurrently endorsing the curriculum to be enjoyable and engaging: 95% *agreed* that they would change their practice because of the curriculum, and 100% *agreed* that doing so would improve the patient care experience. Feedback from SPs regarding FPF performance highlights the importance of providing culturally competent care with strong receptive listening skills.

The PowerPoint materials are a core part of this curriculum and are intended to be consumed as prelearning, either in didactic or self-directed learning format prior to the simulation component. The first two learning objectives, namely articulating the purpose and benefits of the FPF role and listing the four components of the role and key behaviors for each, are level one objectives for knowledge acquisition on Krathwohl's revised Bloom's Taxonomy^25^; however, we strongly believe that when simulation-based health professions education includes novel, high-stakes, and/or emotionally charged concepts, conducting prelearning is necessary prior to entering the concrete experiences stage of the experiential learning cycle. Learning objectives three and four reflect the simulated participant and simulation-based components and necessarily build on the didactic materials.

This curriculum was initially designed for use in emergency department-based pediatric medical resuscitations, and we acknowledge that there are likely differences in traumatic resuscitations, those taking place outside of the emergency department, and those involving adult patients; we are currently adapting the curriculum for use in these areas. Unfortunately, we did not collect data regarding residency type (emergency medicine vs. pediatrics). Additionally, this study was conducted at an academic, quaternary care institution in which staffing models generally permit additional team members to provide family support; however, this may not be true for all settings, especially smaller community-based emergency departments. To address this, we have included guidance in the curriculum regarding how to initiate communication with family members when there is no one to fill the FPF role. Our focus groups comprised youth and family advisory council members from only one institution in the northeastern United States, and their input may not be generalizable to all populations. Other limitations include the use of self-reported, immediate outcomes. We did not assess objective or patient care-focused outcomes, nor did we assess retention of learned skills over time. Because our in situ simulations were conducted monthly and with only one SP, our overall number of responses for the FPF-SAT was small, and we did not perform psychometric assessment of tool performance. Lastly, the workshops and simulation-based components of the curriculum are labor and resource intensive; however, our inclusion of briefer and asynchronous curriculum materials hopefully improves generalizability.

The development of this curriculum benefits from the partnership between clinicians and families, which may have myriad additional advantages. Obtaining the perspective of patients and families in developing educational curricula (particularly simulation-based curricula) may also: improve health outcomes and patient safety; advance key communication skills by learners; mitigate implicit biases and promote health equity; optimize the portrayal of patients and families in curricula and by SPs; and enhance the psychological fidelity of the learning exercise.^26–30^

The key behaviors described in this curriculum are not specific to pediatrics, nor are they specific to emergency department-based medical resuscitations. We believe that family presence during resuscitations should be supported across a wide range of patient ages, medical presentations, and settings. Next steps for research and education include identifying key barriers and facilitators to supporting family presence more widely, linking evidence-based practice to the patient experience and objective health outcomes, and developing best practices for communication and management around patient death when it is experienced by the family and health care teams together. We hope this curriculum will serve as a foundation for clinician-educators in various settings to train interprofessional teams in the FPF role, thereby improving patient- and family-centered care across the spectrum.

## Appendices


FPF Curriculum.pptxFPF Curriculum Recording.mp4Role-Play Script Without FPF.docxRole-Play Script With FPF.docxFPF Participant Worksheet.docxFPF Instructor Worksheet.docxFPF Survey.docxSP Training.pptxSimulated Participant Training Case.docxFPF-SAT.docx
*All appendices are peer reviewed as integral parts of the Original Publication.*

## References

[1] Boudreaux ED, Francis JL, Loyacano T. Family presence during invasive procedures and resuscitations in the emergency department: a critical review and suggestions for future research. Ann Emerg Med. 2002;40(2):193–205. 10.1067/mem.2002.124899 12140499

[2] Joint Commission. *Advancing Effective Communication, Cultural Competence, and Patient- and Family-Centered Care: A Roadmap for Hospitals*. Joint Commission; 2010.

[3] De Stefano C, Normand D, Jabre P, et al. Family presence during resuscitation: a qualitative analysis from a national multicenter randomized clinical trial. PLoS One. 2016;11(6):e0156100. 10.1371/journal.pone.0156100 27253993 PMC4890739

[4] Meyers TA, Eichhorn DJ, Guzzetta CE. Do families want to be present during CPR? A retrospective survey. J Emerg Nurs. 1998;24(5):400–405. 10.1016/S0099-1767(98)70005-4 9814254

[5] ACGME Program Requirements for Graduate Medical Education in Pediatric Emergency. Accreditation Council for Graduate Medical Education; 2023. Accessed September 6, 2024. https://www.acgme.org/globalassets/pfassets/programrequirements/114_pediatricemergencymedicine_2023.pdf

[6] Ratnapalan S, Hilliard RI. Needs assessment in postgraduate medical education: a review. Med Educ Online. 2002;7(1):4542. 10.3402/meo.v7i.4542 28253764

[7] Dudley NC, Hansen KW, Furnival RA, Donaldson AE, Van Wagenen KL, Scaife ER. The effect of family presence on the efficiency of pediatric trauma resuscitations. Ann Emerg Med. 2009;53(6):777–784.e3. 10.1016/j.annemergmed.2008.10.002 19013688

[8] O'Connell KJ, Farah MM, Spandorfer P, Zorc JJ. Family presence during pediatric trauma team activation: an assessment of a structured program. Pediatrics. 2007;120(3):e565–e574. 10.1542/peds.2006-2914 17766498

[9] Deacon A, O'Neill TA, Gilfoyle E. A scoping review of the impact of family presence on pediatric resuscitation team members. Pediatr Crit Care Med. 2020;21(12):e1140–e1147. 10.1097/PCC.0000000000002471 32740185

[10] Dainty KN, Atkins DL, Breckwoldt J, et al; International Liaison Committee on Resuscitation's Pediatric Neonatal Life Support Task Force; Education, Implementation and Teams Task Force. Family presence during resuscitation in paediatric and neonatal cardiac arrest: a systematic review. Resuscitation. 2021;162:20–34. 10.1016/j.resuscitation.2021.01.017 33577966

[11] Curley MAQ, Meyer EC, Scoppettuolo LA, et al. Parent presence during invasive procedures and resuscitation: evaluating a clinical practice change. Am J Respir Crit Care Med. 2012;186(11):1133–1139. 10.1164/rccm.201205-0915OC 22997205

[12] Carney PA, Crites GE, Miller KH, et al. Building and executing a research agenda toward conducting implementation science in medical education. Med Educ Online. 2016;21(1):32405. 10.3402/meo.v21.32405 27565131 PMC5002033

[13] Bajaj K, Meguerdichian M, Thoma B, Huang S, Eppich W, Cheng A. The PEARLS healthcare debriefing tool. Acad Med. 2018;93(2):336. 10.1097/ACM.0000000000002035 29381495

[14] Ghazali DA, Ragot S, Breque C, et al. Randomized controlled trial of multidisciplinary team stress and performance in immersive simulation for management of infant in shock: study protocol. Scand J Trauma Resusc Emerg Med. 2016;24:36. 10.1186/s13049-016-0229-0 27012938 PMC4807574

[15] DeMaria S Jr, Bryson EO, Mooney TJ, et al. Adding emotional stressors to training in simulated cardiopulmonary arrest enhances participant performance. Med Educ. 2010;44(10):1006–1015. 10.1111/j.1365-2923.2010.03775.x 20880370

[16] Peterson EB, Calhoun AW, Rider EA. The reliability of a modified Kalamazoo Consensus Statement Checklist for assessing the communication skills of multidisciplinary clinicians in the simulated environment. Patient Educ Couns. 2014;96(3):411–418. 10.1016/j.pec.2014.07.013 25103180

[17] Chiu CJ. Development and Validation of Performance Assessment Tools for Interprofessional Communication and Teamwork (PACT). Dissertation. University of Washington; 2014.

[18] TeamSTEPPS Team Performance Observation Tool. Agency for Healthcare Research and Quality. Accessed July 19, 2024. https://www.ahrq.gov/teamstepps/instructor/reference/tmpot.html

[19] Malec JF, Torsher LC, Dunn WF, et al. The Mayo High Performance Teamwork Scale: reliability and validity for evaluating key crew resource management skills. Simul Healthc. 2007;2(1):4–10. 10.1097/SIH.0b013e31802b68ee 19088602

[20] Sala Patient and Family Faculty Program Patient and Family Centered Scorecard. Sala Patient & Family Faculty Program. Hassenfeld Children's Hospital at NYU Langone Health. Accessed March 26, 2021. https://nyulangone.org/locations/hassenfeld-childrens-hospital/sala-child-family-support/family-partnership-programs/sala-patient-family-faculty-program

[21] Sala Institute for Child & Family Centered Care. Hassenfeld Children's Hospital at NYU Langone. Accessed March 26, 2021. https://nyulangone.org/locations/hassenfeld-childrens-hospital/about-hassenfeld-childrens-hospital/sala-institute-for-child-family-centered-care

[22] Thomas PA, Kern DE, Hughes MT, Chen BY, eds. Curriculum Development for Medical Education: A Six-Step Approach. Johns Hopkins University Press; 2016.

[23] Agnant J, Napoli K. Family Presence Facilitator: support during trauma and medical resuscitation training and resources. PowerPoint presented at: Bellevue Hospital Center Interprofessional Weekly Trauma Conference; October 2019; New York, NY.

[24] Kirkpatrick DL, Kirkpatrick JD. Evaluating Training Programs: The Four Levels. 3rd ed. Berrett-Koehler; 2006.

[25] Krathwohl DR. A revision of Bloom's Taxonomy: an overview. Theory Pract. 2002;41(4):212–218. 10.1207/s15430421tip4104_2

[26] DiGioia AM III, Greenhouse PK. Creating value with the patient- and family-centered care methodology and practice: what trainees need to know, why, and strategies for medical education. AMA J Ethics. 2016;18(1):33–39. 10.1001/journalofethics.2016.18.1.medu2-1601 26854634

[27] Millenson ML, Shapiro E, Greenhouse PK, DiGioia AM III. Patient- and family-centered care: a systematic approach to better ethics and care. AMA J Ethics. 2016;18(1):49–55. 10.1001/journalofethics.2016.18.1.stas1-1601 26854636

[28] Diskin C, Robinson K, Agrawal R, Masterson D, Coleman C, Cohen E. Family partnership in continuing medical education: a collaborative experience. Pediatrics. 2023;151(5):e2022060280. 10.1542/peds.2022-060280 37013694

[29] Rees P, Wimberg J, Walsh KE. Patient and family partnership for safer health care. Pediatrics. 2018;142(3):e20172847. 10.1542/peds.2017-2847 30087198

[30] Kneebone R, Nestel D, Wetzel C, et al. The human face of simulation: patient-focused simulation training. Acad Med. 2006;81(10):919–924. 10.1097/01.ACM.0000238323.73623.c2 16985358

