# Supplementary material for: Development and Implementation of a Family Presence Facilitator Curriculum for Interprofessional Use in Pediatric Medical Resuscitations
Source: MedEdPORTAL. 2024 Oct 8;20:11445. doi: 10.15766/mep_2374-8265.11445 (PMC11458738; doi:10.15766/mep_2374-8265.11445)

## Slide 1
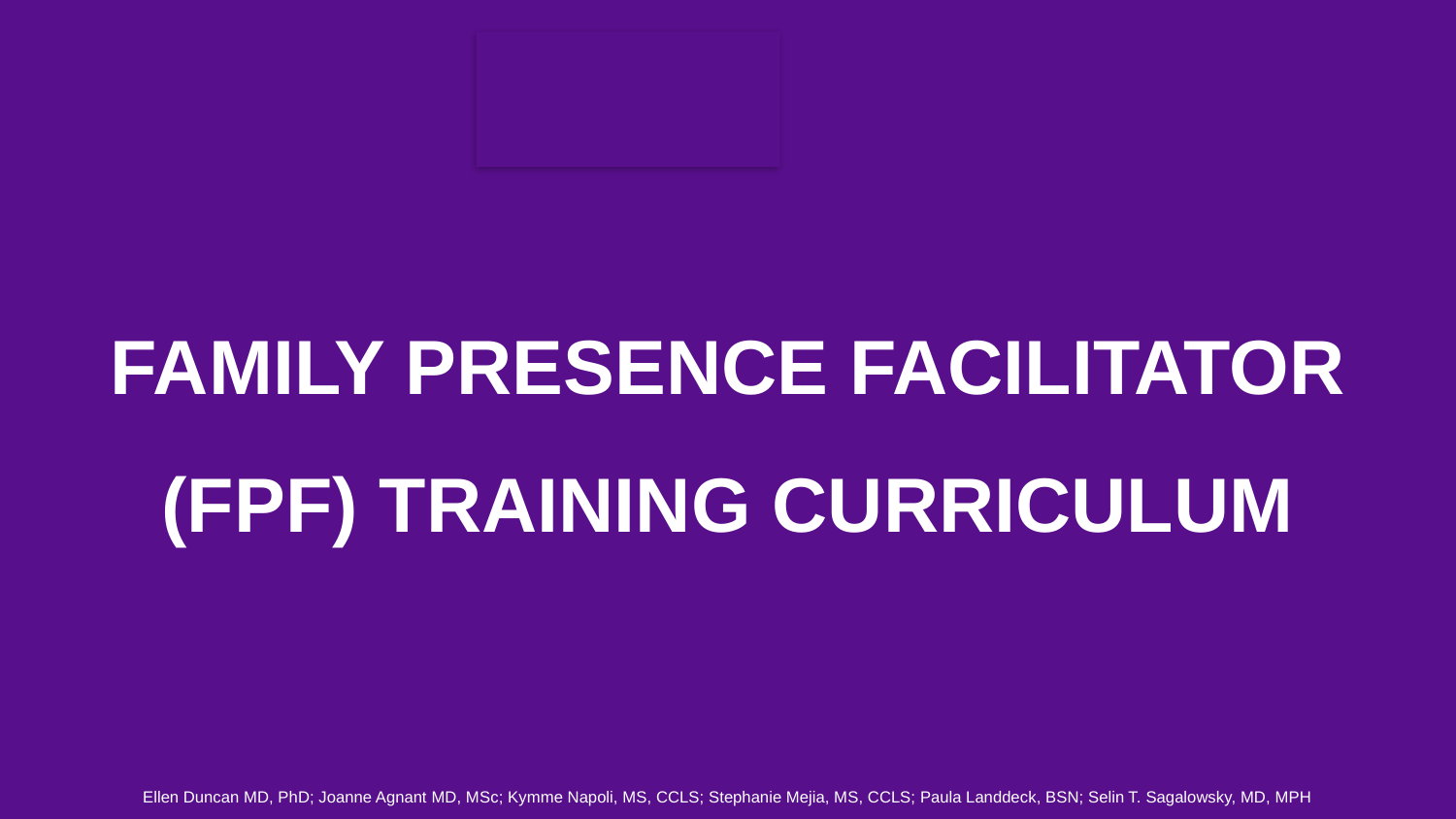

# Family presence facilitator (FPF) Training curriculum
Ellen Duncan MD, PhD; Joanne Agnant MD, MSc; Kymme Napoli, MS, CCLS; Stephanie Mejia, MS, CCLS; Paula Landdeck, BSN; Selin T. Sagalowsky, MD, MPH

## Slide 2
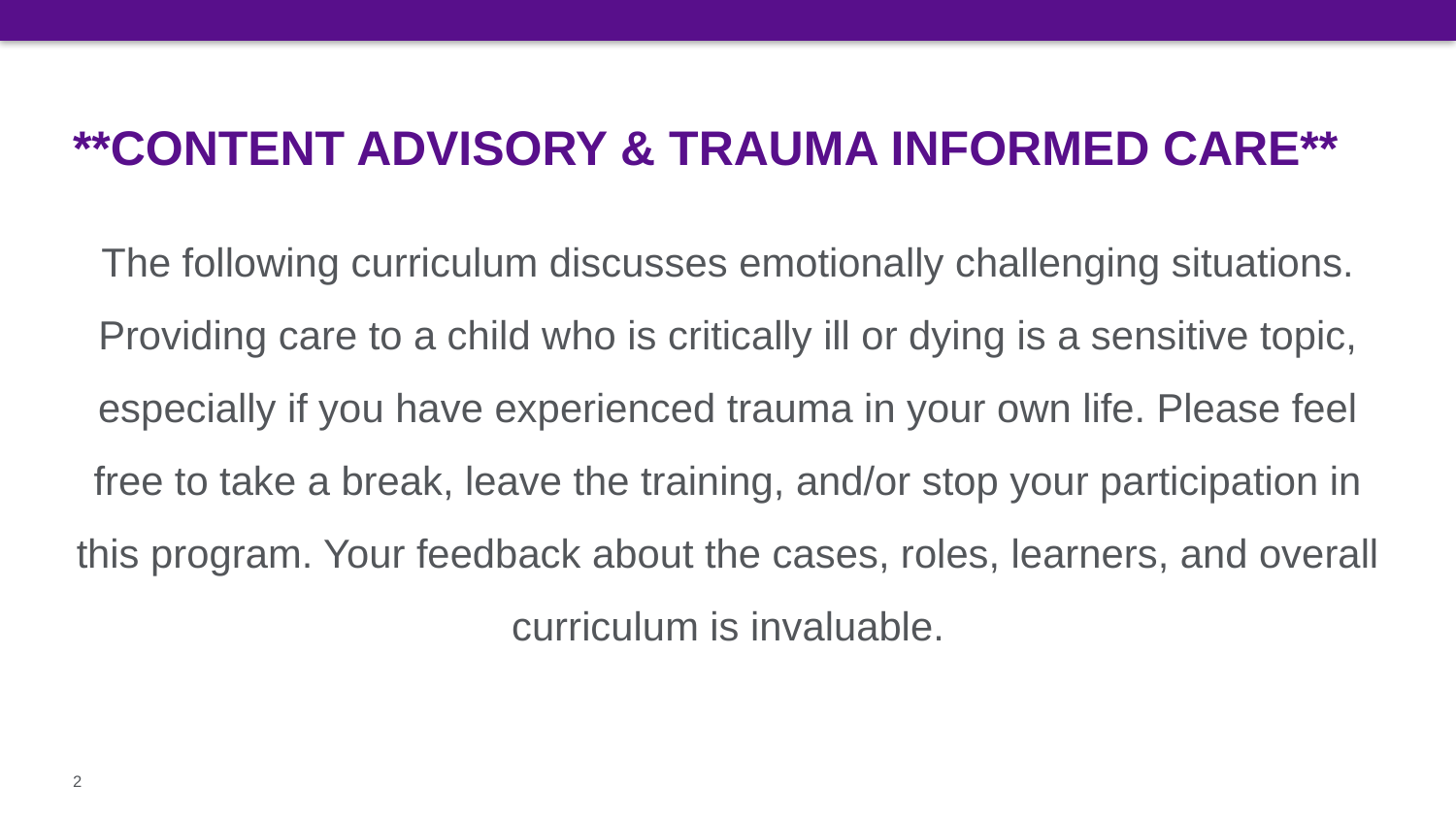

# **CONTENT ADVISORY & TRAUMA INFORMED CARE**
The following curriculum discusses emotionally challenging situations. Providing care to a child who is critically ill or dying is a sensitive topic, especially if you have experienced trauma in your own life. Please feel free to take a break, leave the training, and/or stop your participation in this program. Your feedback about the cases, roles, learners, and overall curriculum is invaluable.
2

## Slide 3
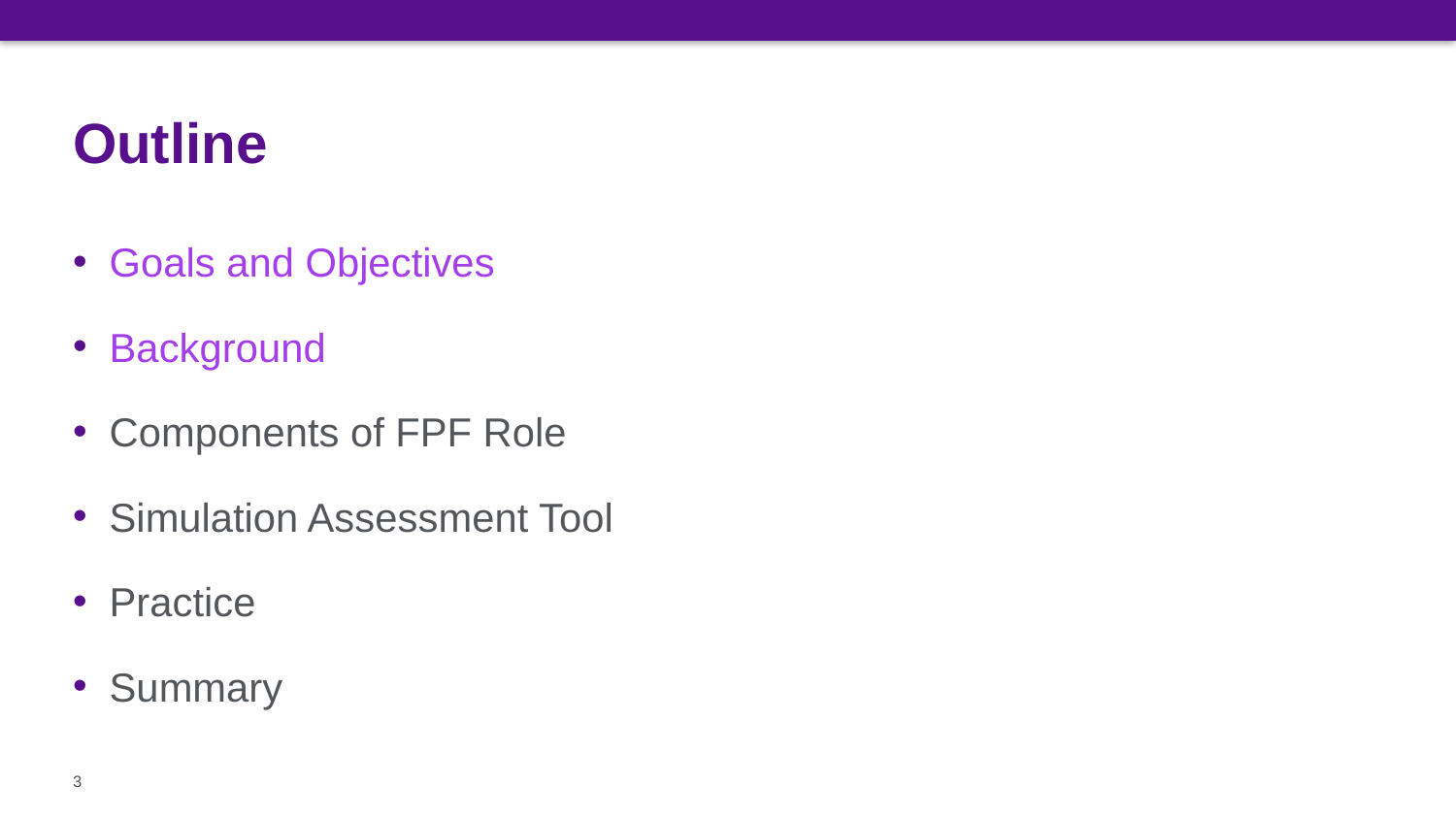

# Outline
Goals and Objectives
Background
Components of FPF Role
Simulation Assessment Tool
Practice
Summary
3

## Slide 4
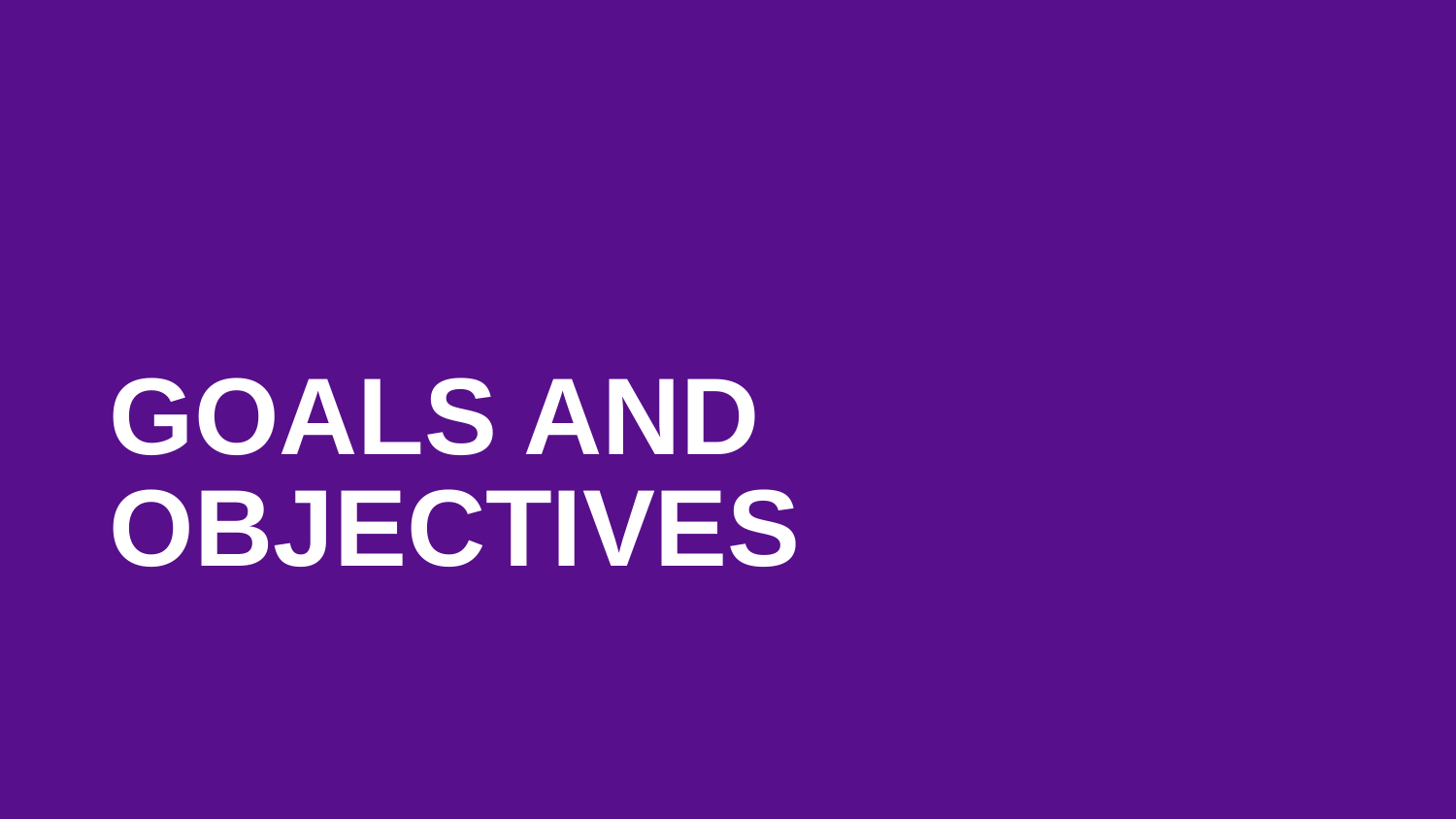

# GOALS and OBJECTIVES

## Slide 5
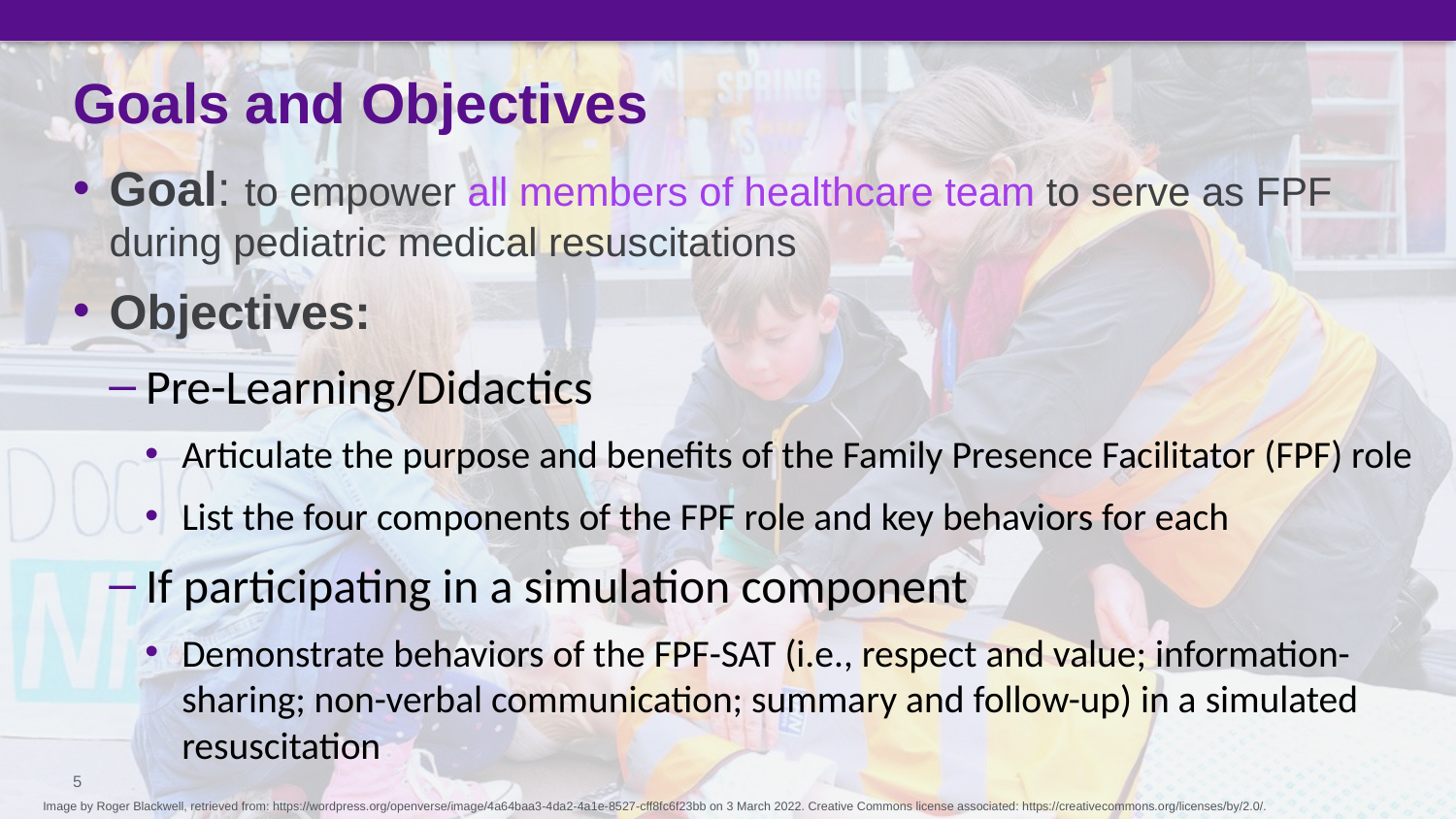

# Goals and Objectives
Goal: to empower all members of healthcare team to serve as FPF during pediatric medical resuscitations
Objectives:
Pre-Learning/Didactics
Articulate the purpose and benefits of the Family Presence Facilitator (FPF) role
List the four components of the FPF role and key behaviors for each
If participating in a simulation component
Demonstrate behaviors of the FPF-SAT (i.e., respect and value; information-sharing; non-verbal communication; summary and follow-up) in a simulated resuscitation
5
Image by Roger Blackwell, retrieved from: https://wordpress.org/openverse/image/4a64baa3-4da2-4a1e-8527-cff8fc6f23bb on 3 March 2022. Creative Commons license associated: https://creativecommons.org/licenses/by/2.0/.

## Slide 6
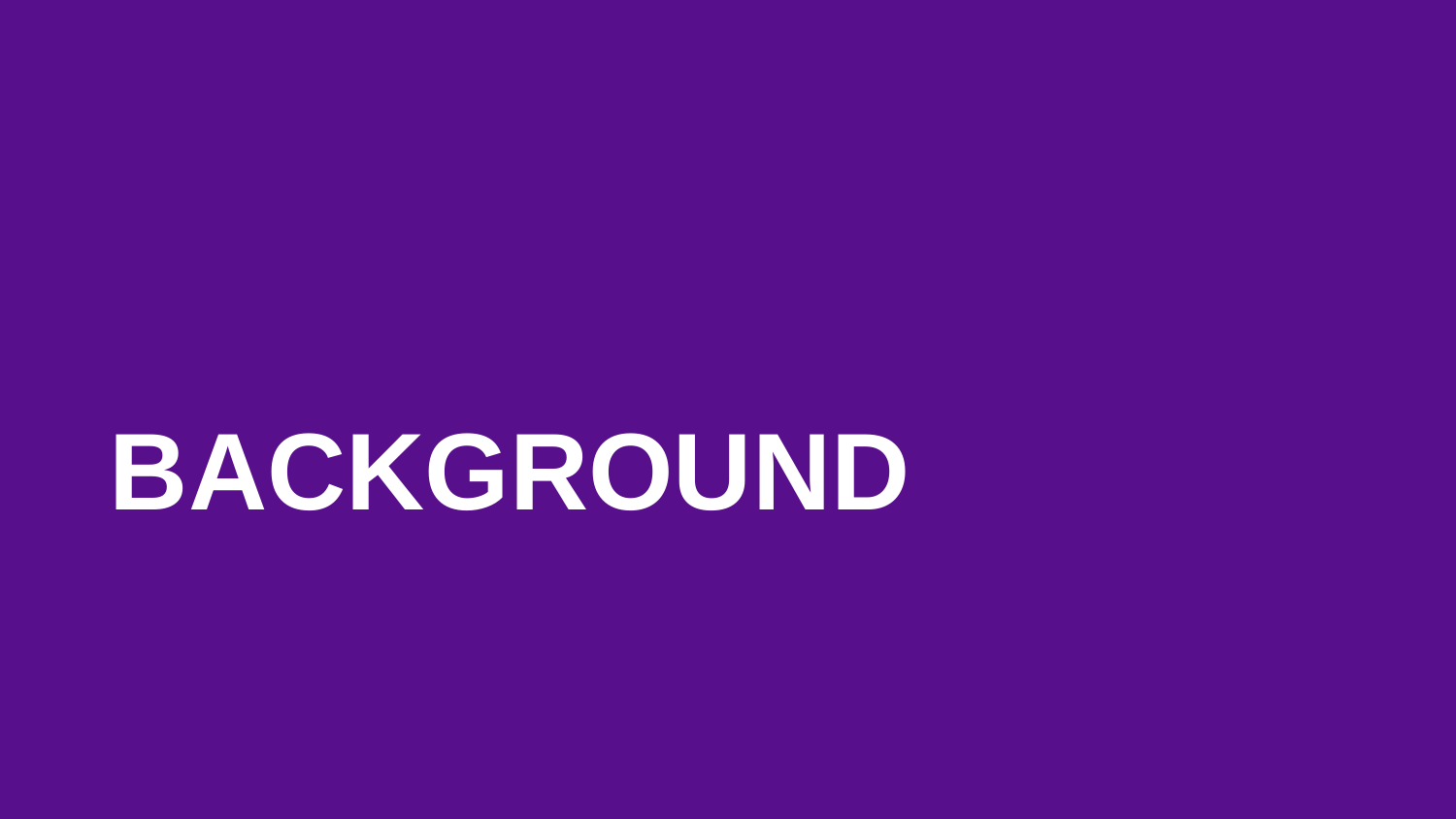

# Background

## Slide 7
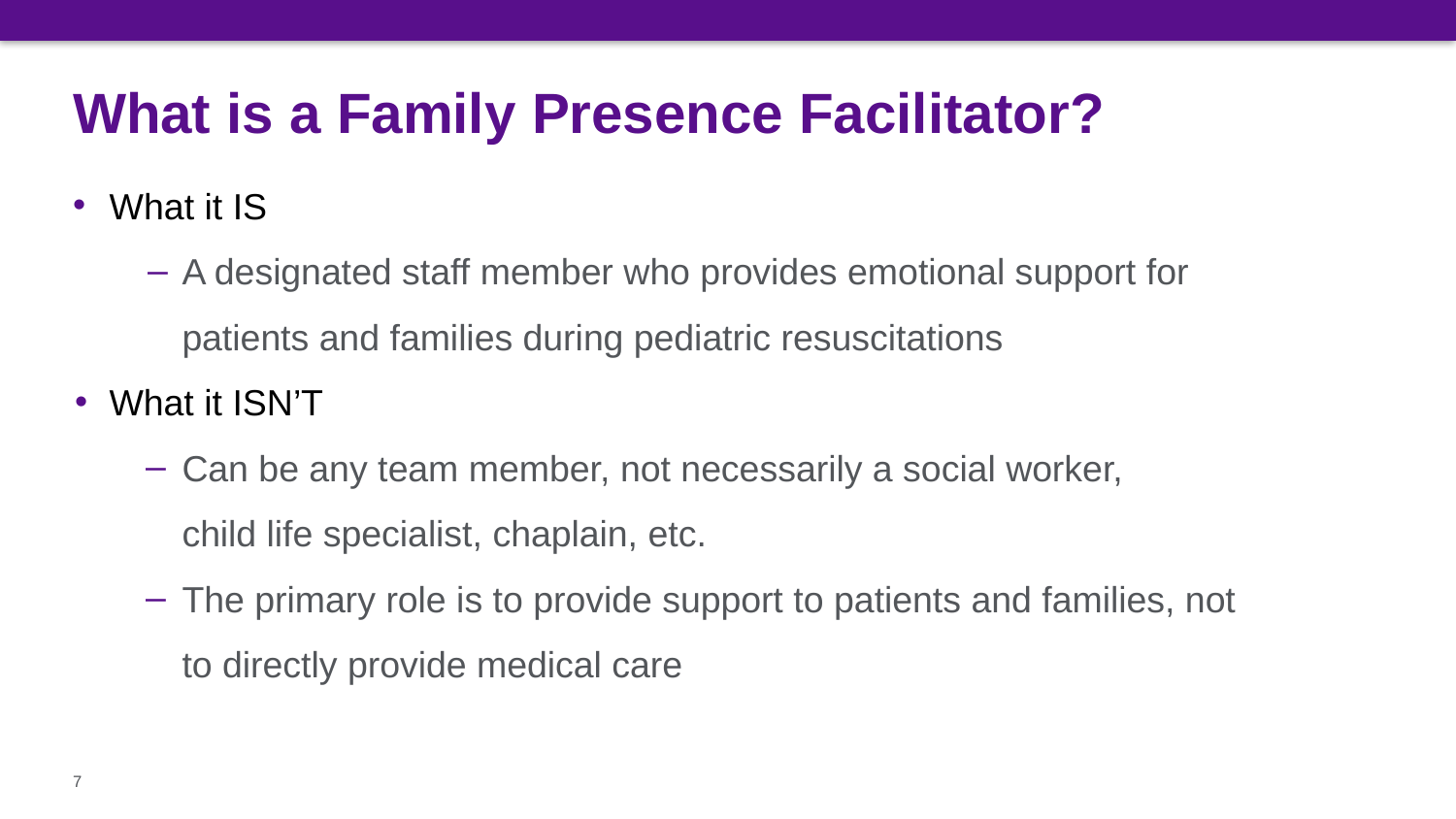

# What is a Family Presence Facilitator?
What it IS
A designated staff member who provides emotional support for patients and families during pediatric resuscitations
What it ISN’T
Can be any team member, not necessarily a social worker, child life specialist, chaplain, etc.
The primary role is to provide support to patients and families, not to directly provide medical care
7

## Slide 8
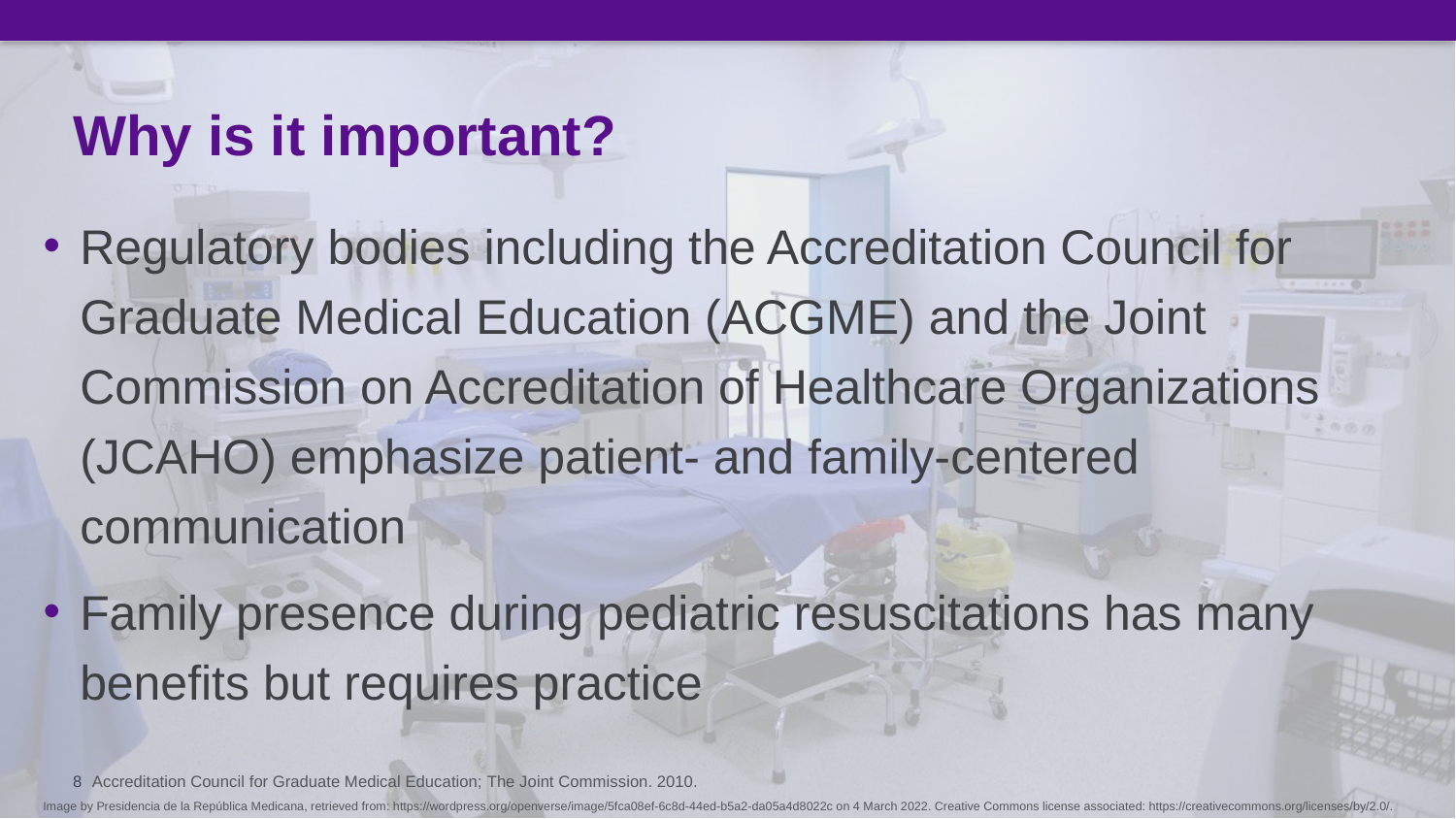

# Why is it important?
Regulatory bodies including the Accreditation Council for Graduate Medical Education (ACGME) and the Joint Commission on Accreditation of Healthcare Organizations (JCAHO) emphasize patient- and family-centered communication
Family presence during pediatric resuscitations has many benefits but requires practice
 Accreditation Council for Graduate Medical Education; The Joint Commission. 2010.
8
Image by Presidencia de la República Medicana, retrieved from: https://wordpress.org/openverse/image/5fca08ef-6c8d-44ed-b5a2-da05a4d8022c on 4 March 2022. Creative Commons license associated: https://creativecommons.org/licenses/by/2.0/.

## Slide 9
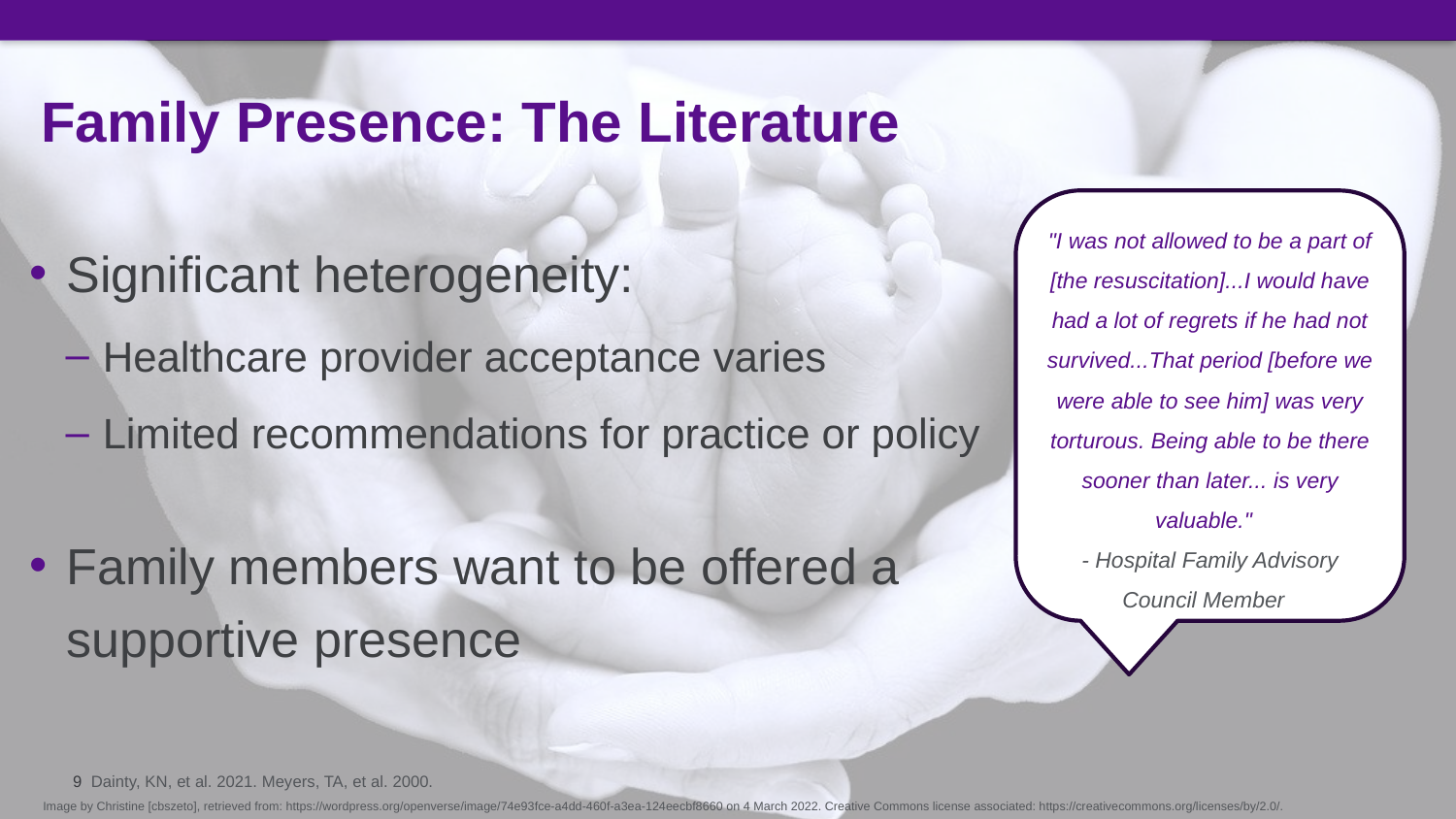

# Family Presence: The Literature
"I was not allowed to be a part of [the resuscitation]...I would have had a lot of regrets if he had not survived...That period [before we were able to see him] was very torturous. Being able to be there sooner than later... is very valuable."
- Hospital Family Advisory Council Member
Significant heterogeneity:
Healthcare provider acceptance varies
Limited recommendations for practice or policy
Family members want to be offered a supportive presence
Dainty, KN, et al. 2021. Meyers, TA, et al. 2000.
9
Image by Christine [cbszeto], retrieved from: https://wordpress.org/openverse/image/74e93fce-a4dd-460f-a3ea-124eecbf8660 on 4 March 2022. Creative Commons license associated: https://creativecommons.org/licenses/by/2.0/.

## Slide 10
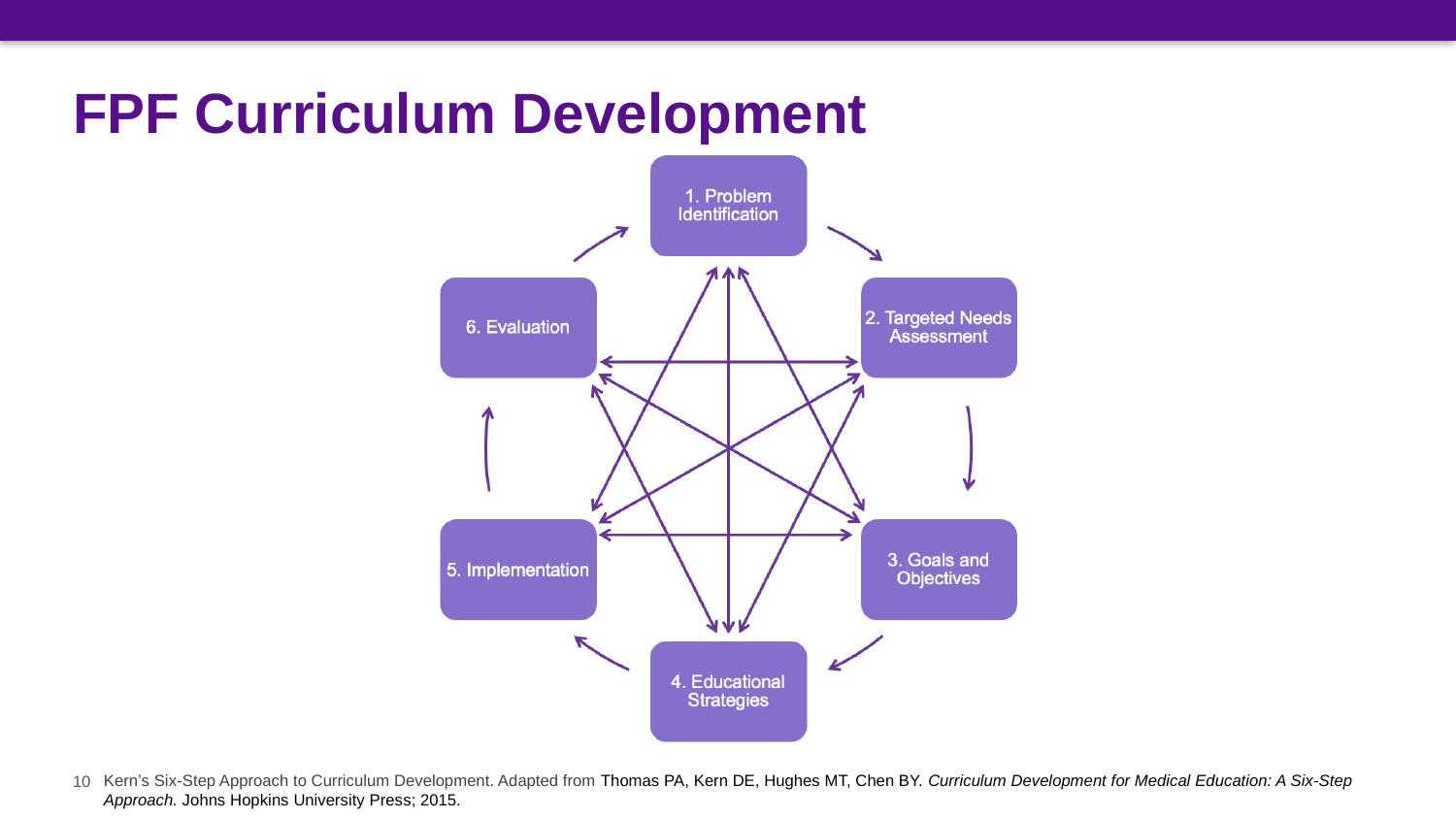

# FPF Curriculum Development
Kern’s Six-Step Approach to Curriculum Development. Adapted from Thomas PA, Kern DE, Hughes MT, Chen BY. Curriculum Development for Medical Education: A Six-Step Approach. Johns Hopkins University Press; 2015.
10

## Slide 11
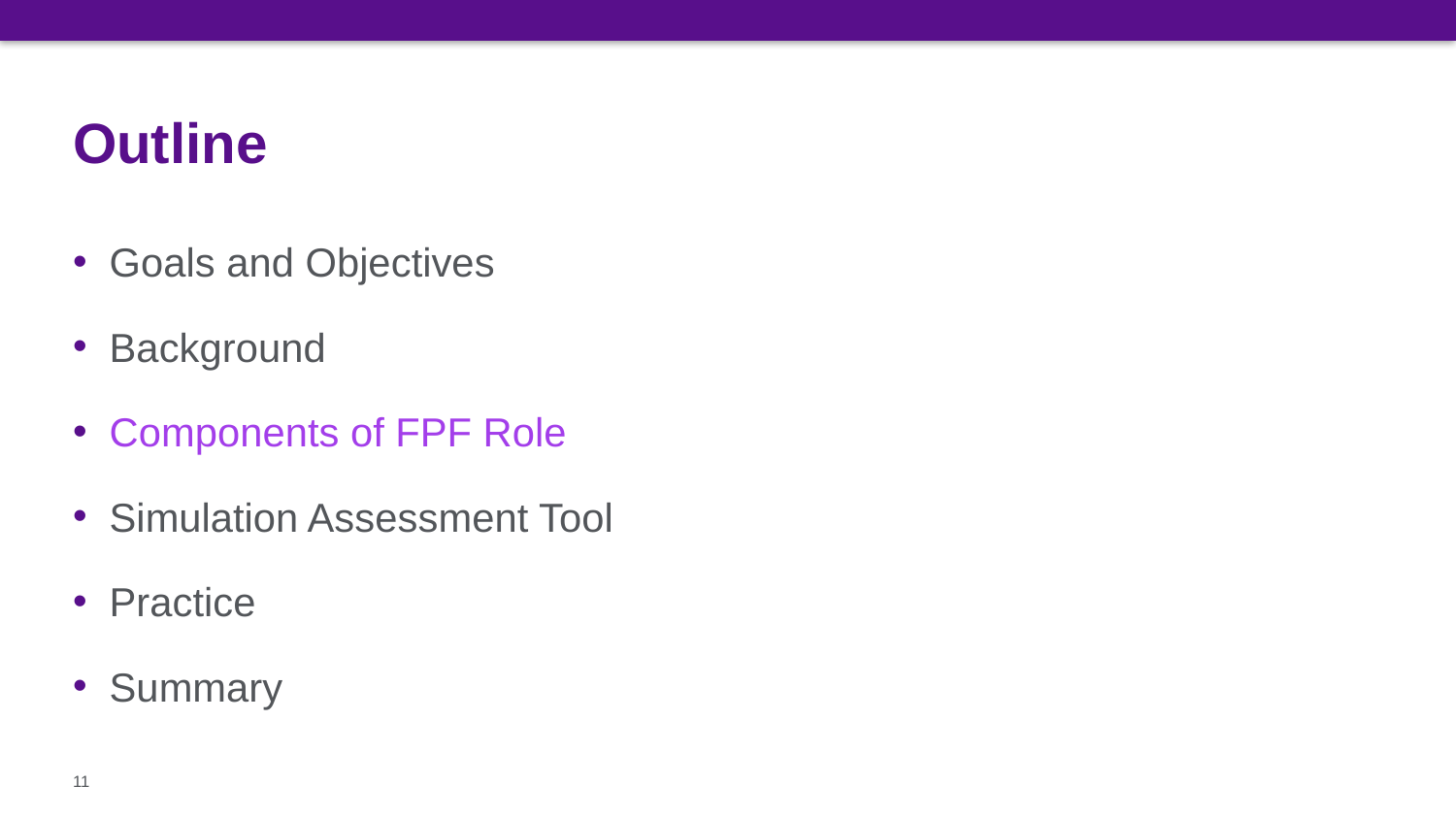

# Outline
Goals and Objectives
Background
Components of FPF Role
Simulation Assessment Tool
Practice
Summary
11

## Slide 12
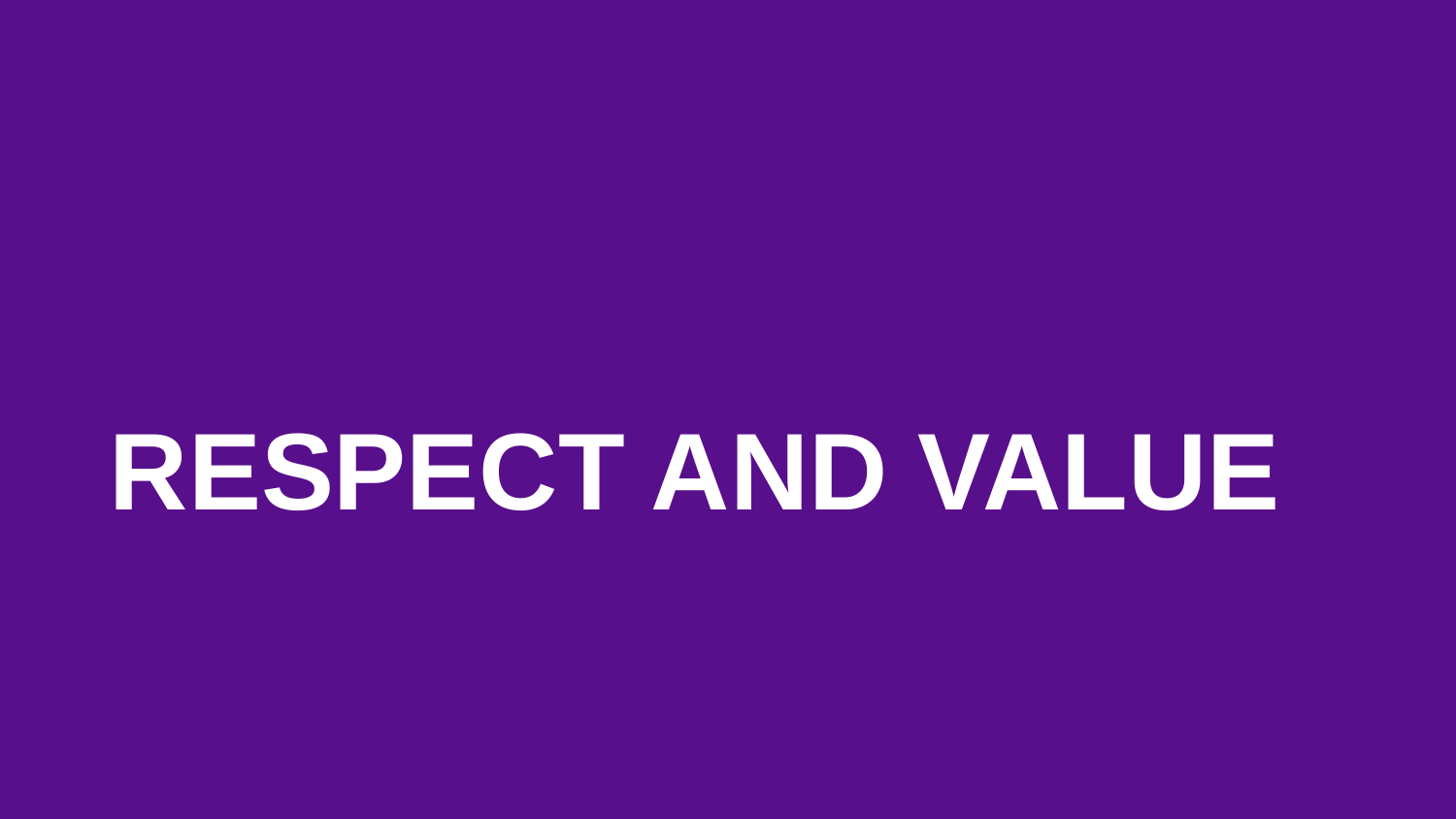

# Respect and value

## Slide 13
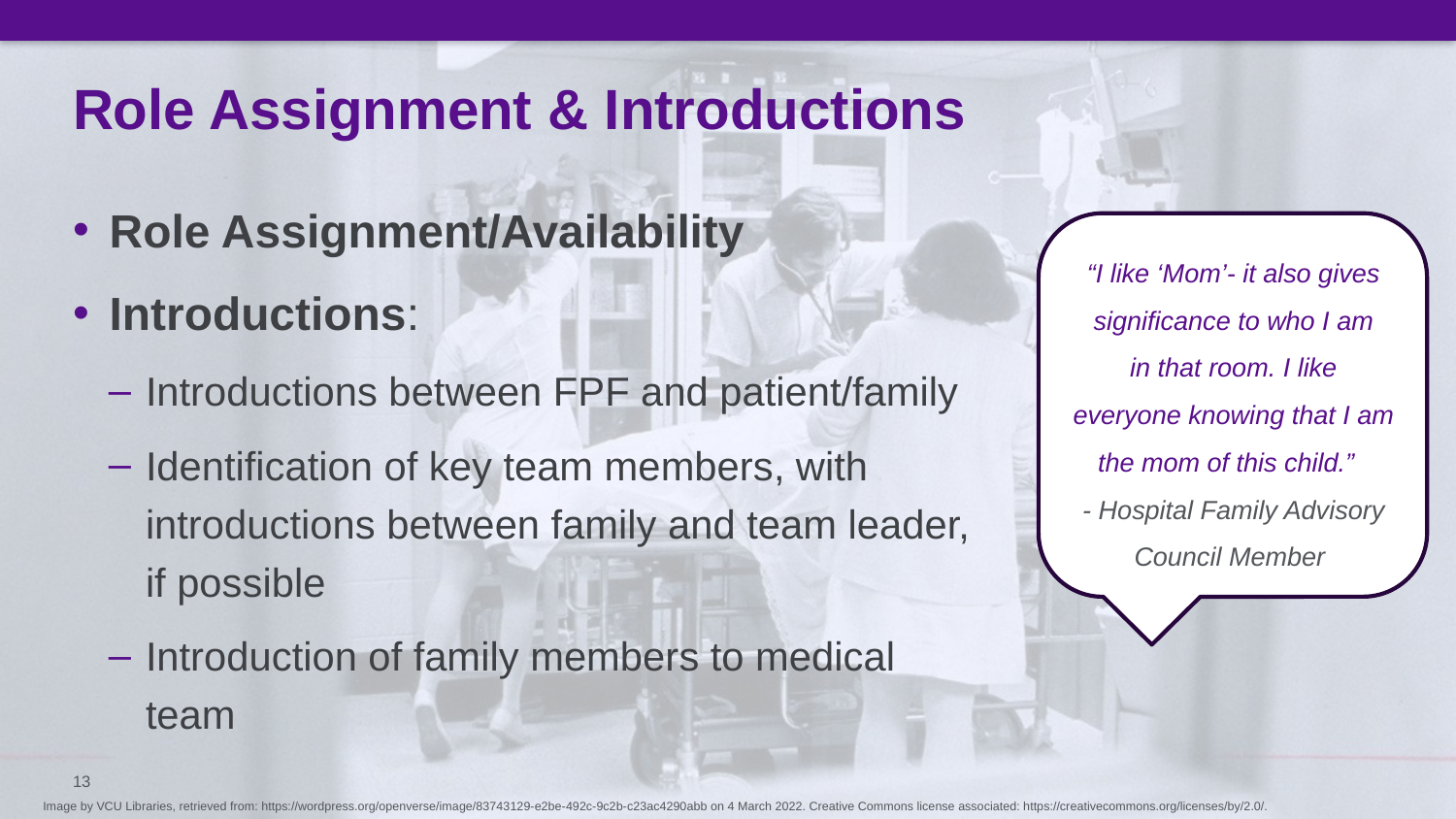

# Role Assignment & Introductions
Role Assignment/Availability
Introductions:
Introductions between FPF and patient/family
Identification of key team members, with introductions between family and team leader, if possible
Introduction of family members to medical team
“I like ‘Mom’- it also gives significance to who I am in that room. I like everyone knowing that I am the mom of this child.”
- Hospital Family Advisory Council Member
13
Image by VCU Libraries, retrieved from: https://wordpress.org/openverse/image/83743129-e2be-492c-9c2b-c23ac4290abb on 4 March 2022. Creative Commons license associated: https://creativecommons.org/licenses/by/2.0/.

## Slide 14
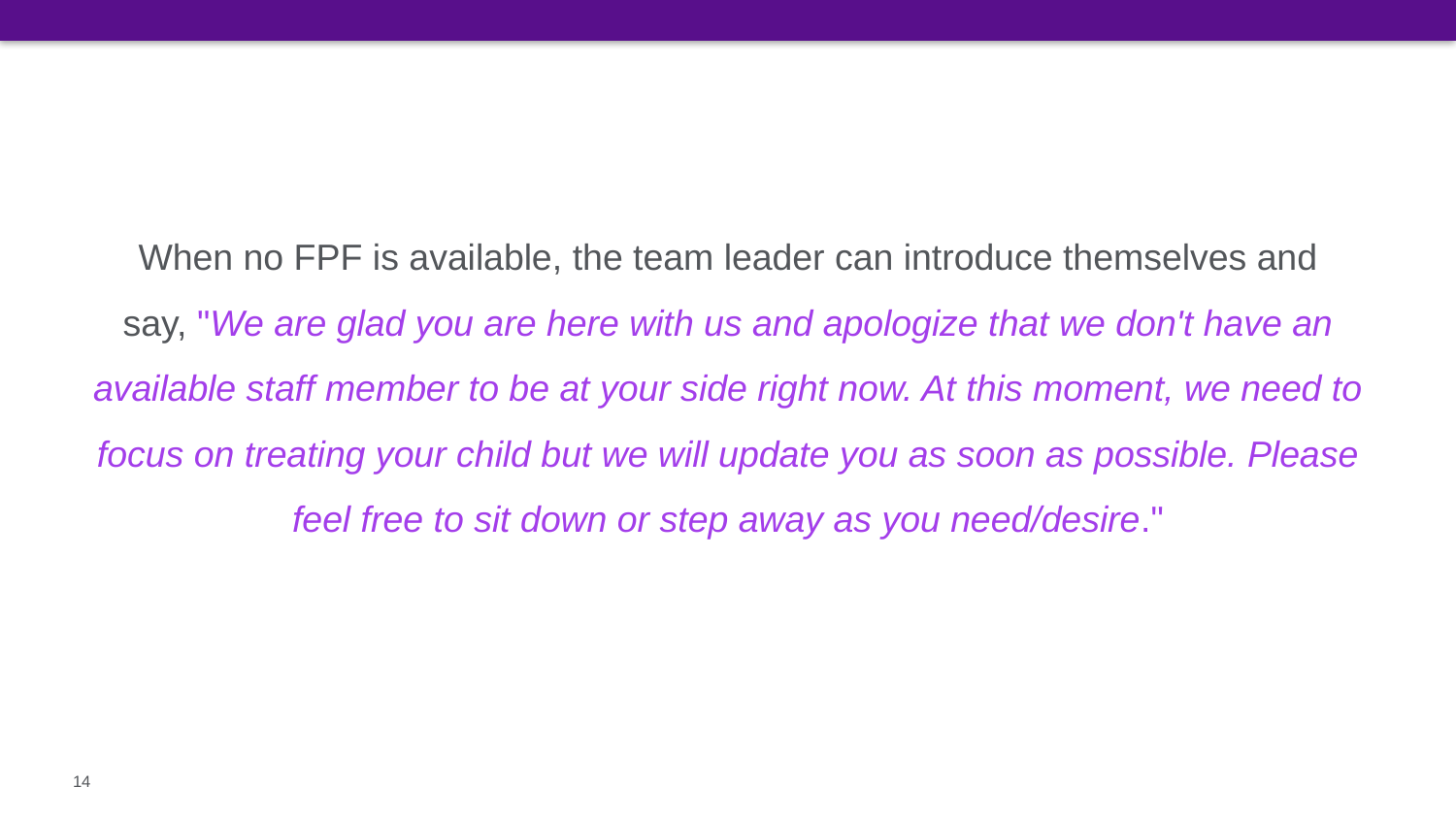

When no FPF is available, the team leader can introduce themselves and say, "We are glad you are here with us and apologize that we don't have an available staff member to be at your side right now. At this moment, we need to focus on treating your child but we will update you as soon as possible. Please feel free to sit down or step away as you need/desire."
14

## Slide 15
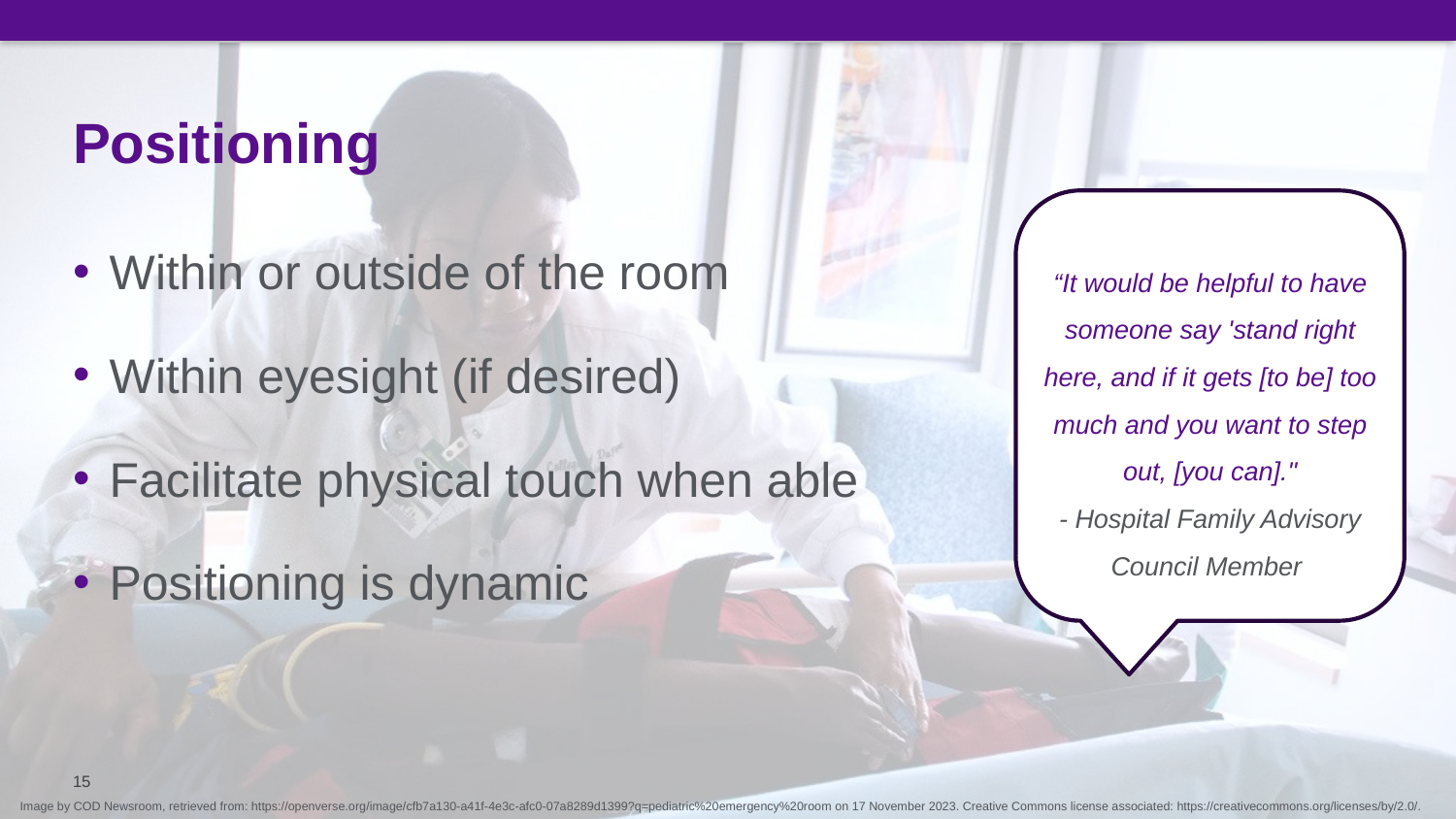

# Positioning
Within or outside of the room
Within eyesight (if desired)
Facilitate physical touch when able
Positioning is dynamic
“It would be helpful to have someone say 'stand right here, and if it gets [to be] too much and you want to step out, [you can]."
- Hospital Family Advisory Council Member
15
Image by COD Newsroom, retrieved from: https://openverse.org/image/cfb7a130-a41f-4e3c-afc0-07a8289d1399?q=pediatric%20emergency%20room on 17 November 2023. Creative Commons license associated: https://creativecommons.org/licenses/by/2.0/.

## Slide 16
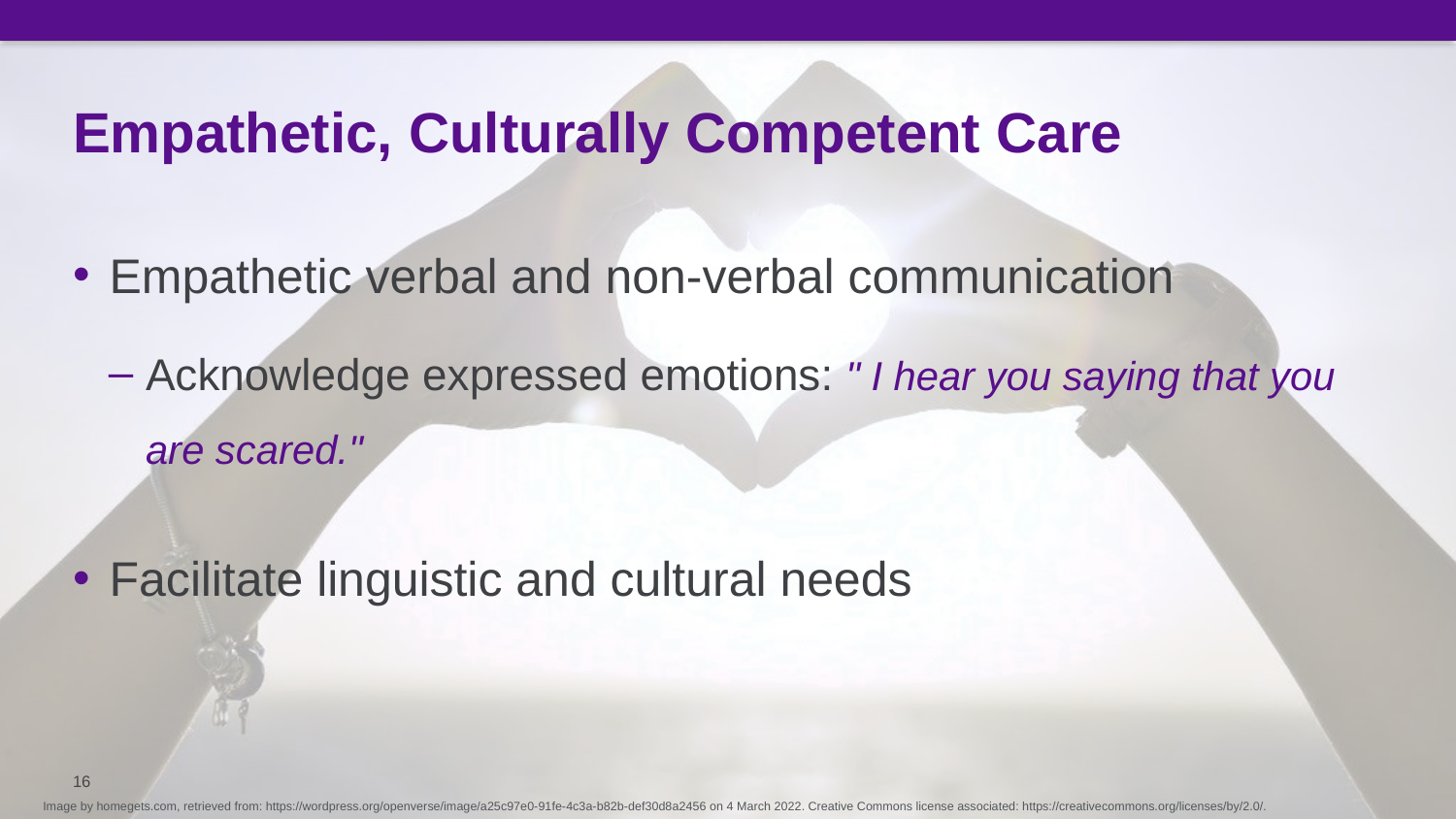

# Empathetic, Culturally Competent Care
Empathetic verbal and non-verbal communication
Acknowledge expressed emotions: " I hear you saying that you are scared."
Facilitate linguistic and cultural needs
16
Image by homegets.com, retrieved from: https://wordpress.org/openverse/image/a25c97e0-91fe-4c3a-b82b-def30d8a2456 on 4 March 2022. Creative Commons license associated: https://creativecommons.org/licenses/by/2.0/.

## Slide 17
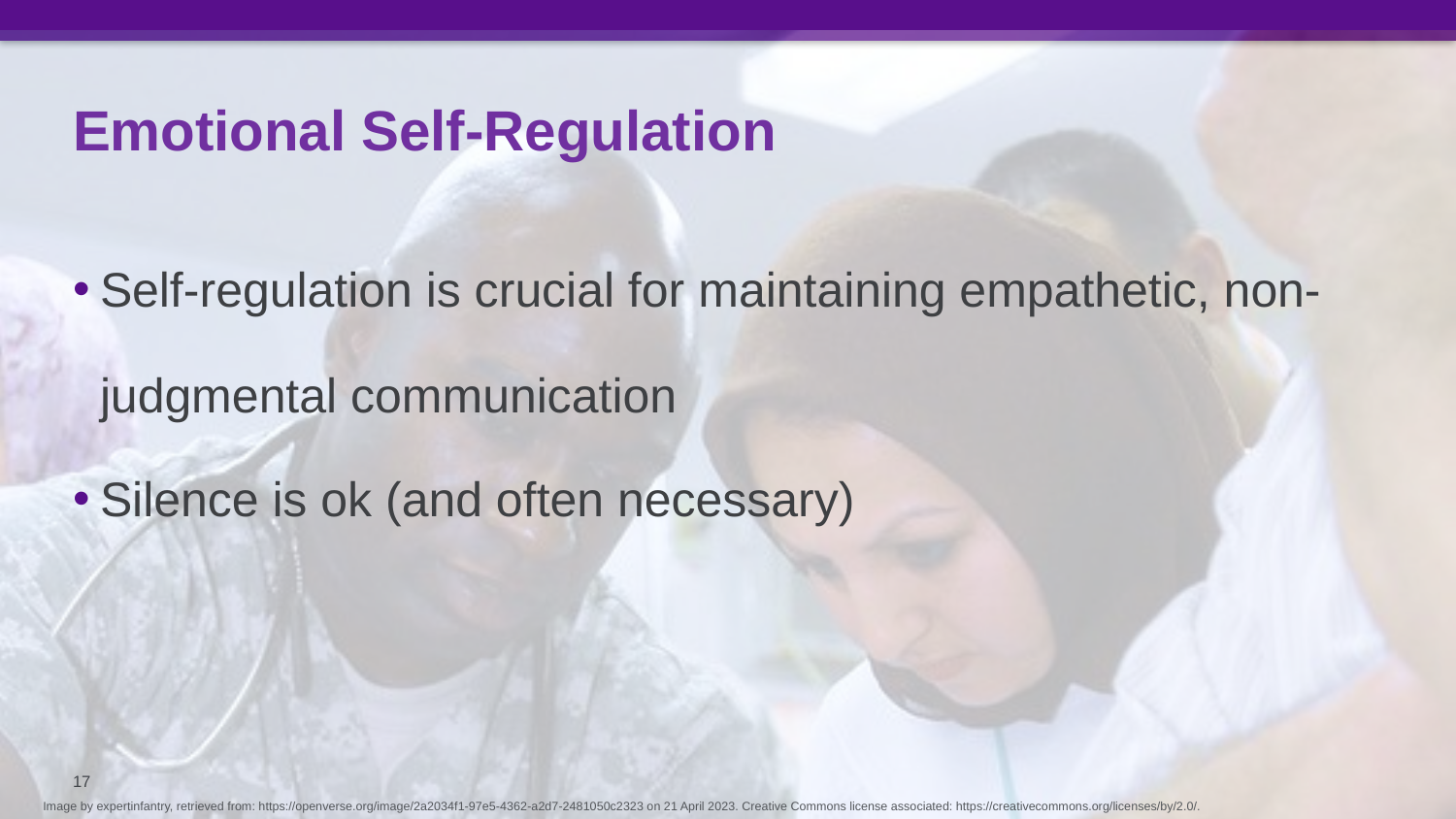

# Emotional Self-Regulation
Self-regulation is crucial for maintaining empathetic, non-judgmental communication
Silence is ok (and often necessary)
17
Image by expertinfantry, retrieved from: https://openverse.org/image/2a2034f1-97e5-4362-a2d7-2481050c2323 on 21 April 2023. Creative Commons license associated: https://creativecommons.org/licenses/by/2.0/.

## Slide 18
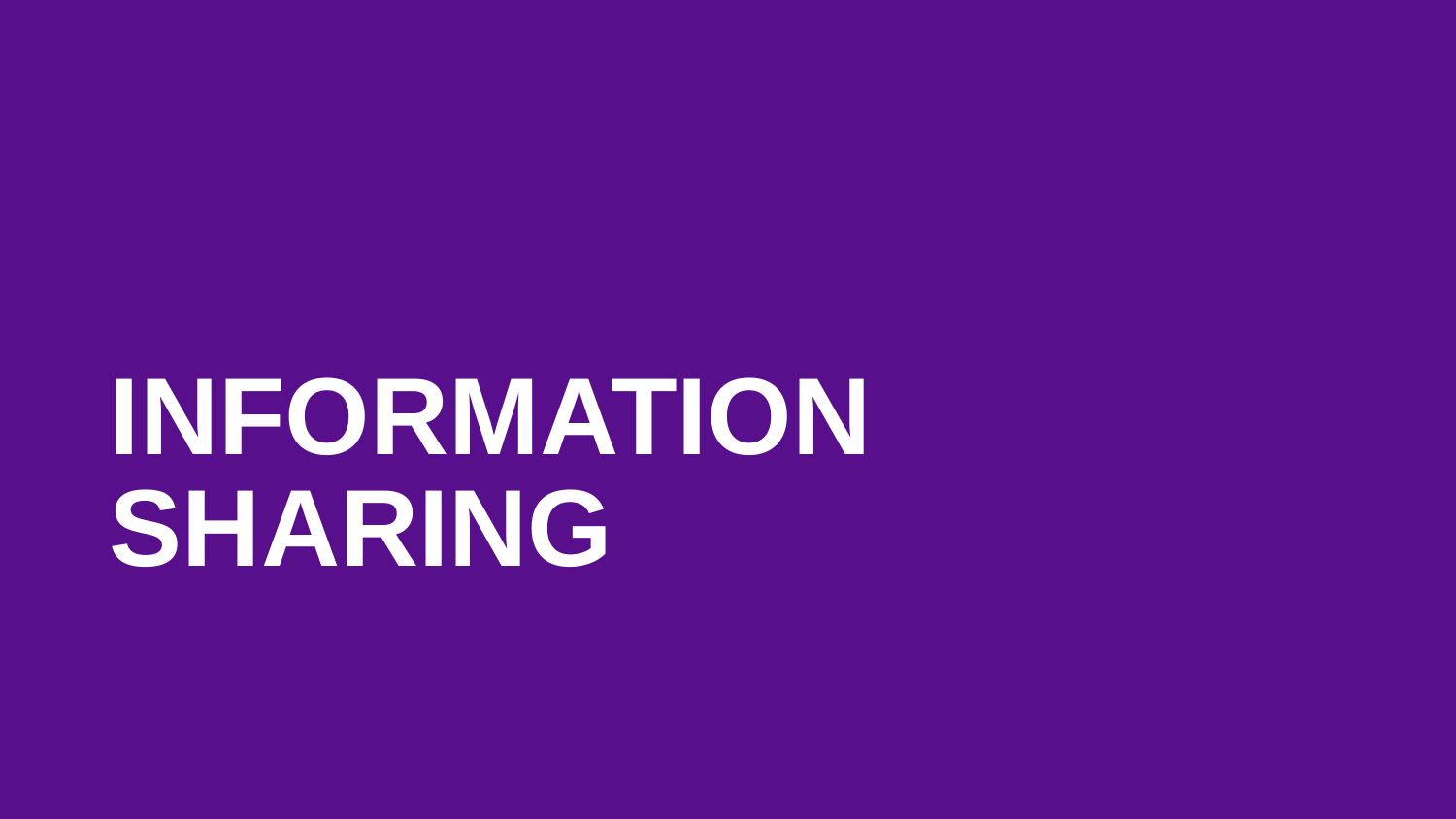

# Information sharing

## Slide 19
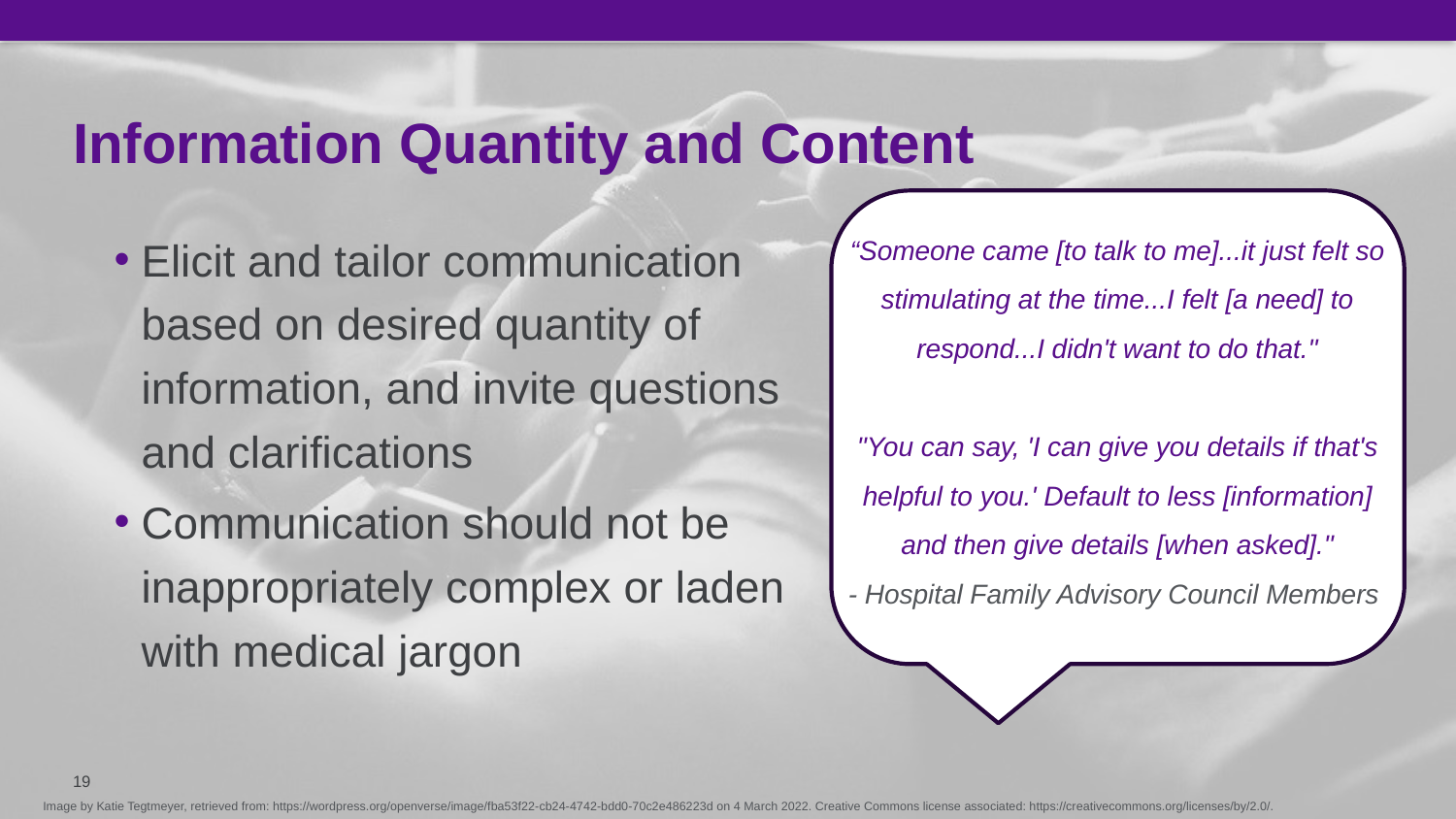

# Information Quantity and Content
“Someone came [to talk to me]...it just felt so stimulating at the time...I felt [a need] to respond...I didn't want to do that."
"You can say, 'I can give you details if that's helpful to you.' Default to less [information] and then give details [when asked]."
- Hospital Family Advisory Council Members
Elicit and tailor communication based on desired quantity of information, and invite questions and clarifications
Communication should not be inappropriately complex or laden with medical jargon
19
Image by Katie Tegtmeyer, retrieved from: https://wordpress.org/openverse/image/fba53f22-cb24-4742-bdd0-70c2e486223d on 4 March 2022. Creative Commons license associated: https://creativecommons.org/licenses/by/2.0/.

## Slide 20
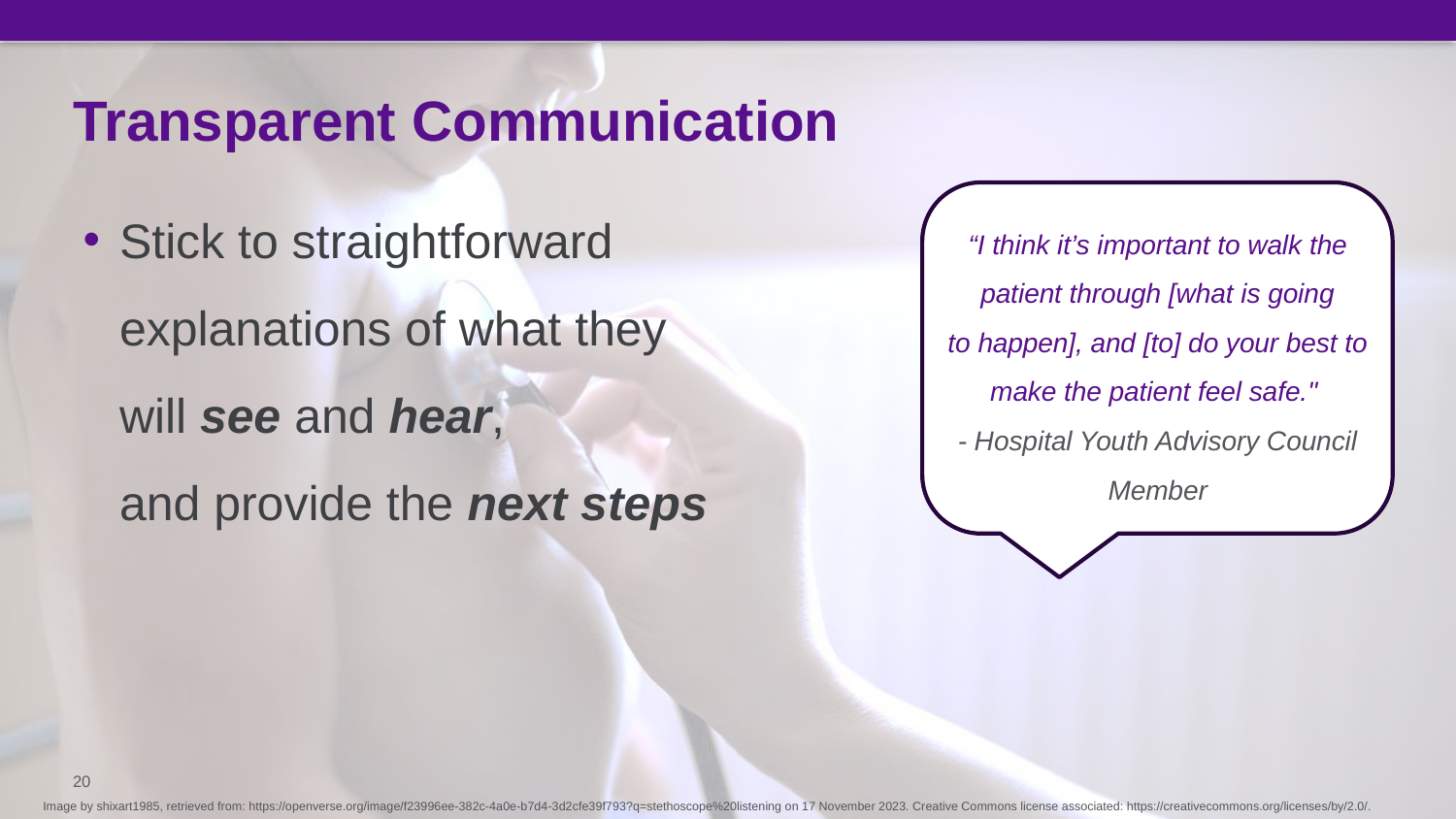

# Transparent Communication
Stick to straightforward explanations of what they will see and hear, and provide the next steps
“I think it’s important to walk the patient through [what is going to happen], and [to] do your best to make the patient feel safe."
- Hospital Youth Advisory Council Member
20
Image by shixart1985, retrieved from: https://openverse.org/image/f23996ee-382c-4a0e-b7d4-3d2cfe39f793?q=stethoscope%20listening on 17 November 2023. Creative Commons license associated: https://creativecommons.org/licenses/by/2.0/.

## Slide 21
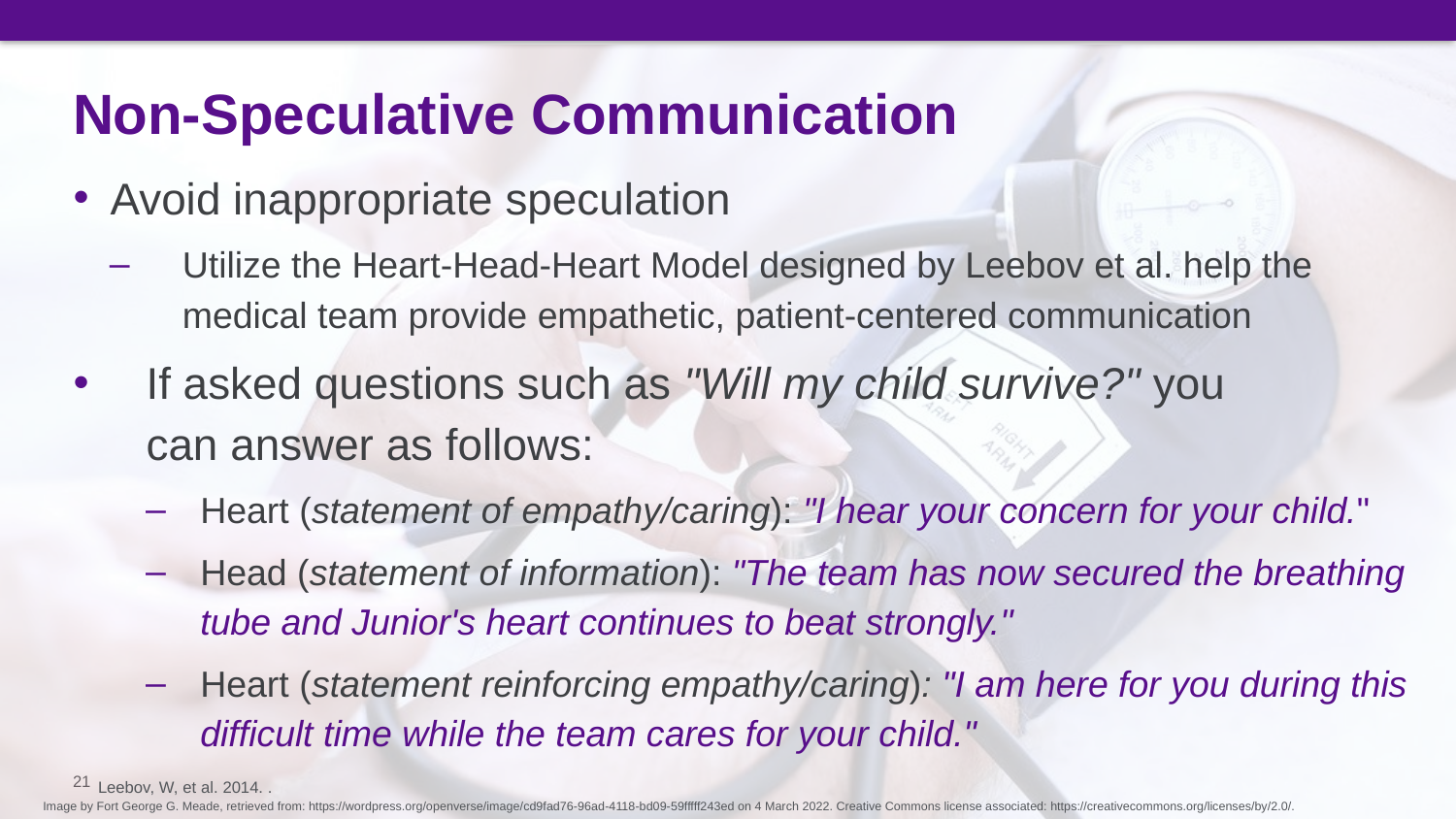

# Non-Speculative Communication
Avoid inappropriate speculation
Utilize the Heart-Head-Heart Model designed by Leebov et al. help the medical team provide empathetic, patient-centered communication
If asked questions such as "Will my child survive?" you can answer as follows:
Heart (statement of empathy/caring): "I hear your concern for your child."
Head (statement of information): "The team has now secured the breathing tube and Junior's heart continues to beat strongly."
Heart (statement reinforcing empathy/caring): "I am here for you during this difficult time while the team cares for your child."
Leebov, W, et al. 2014. .
21
Image by Fort George G. Meade, retrieved from: https://wordpress.org/openverse/image/cd9fad76-96ad-4118-bd09-59fffff243ed on 4 March 2022. Creative Commons license associated: https://creativecommons.org/licenses/by/2.0/.

## Slide 22
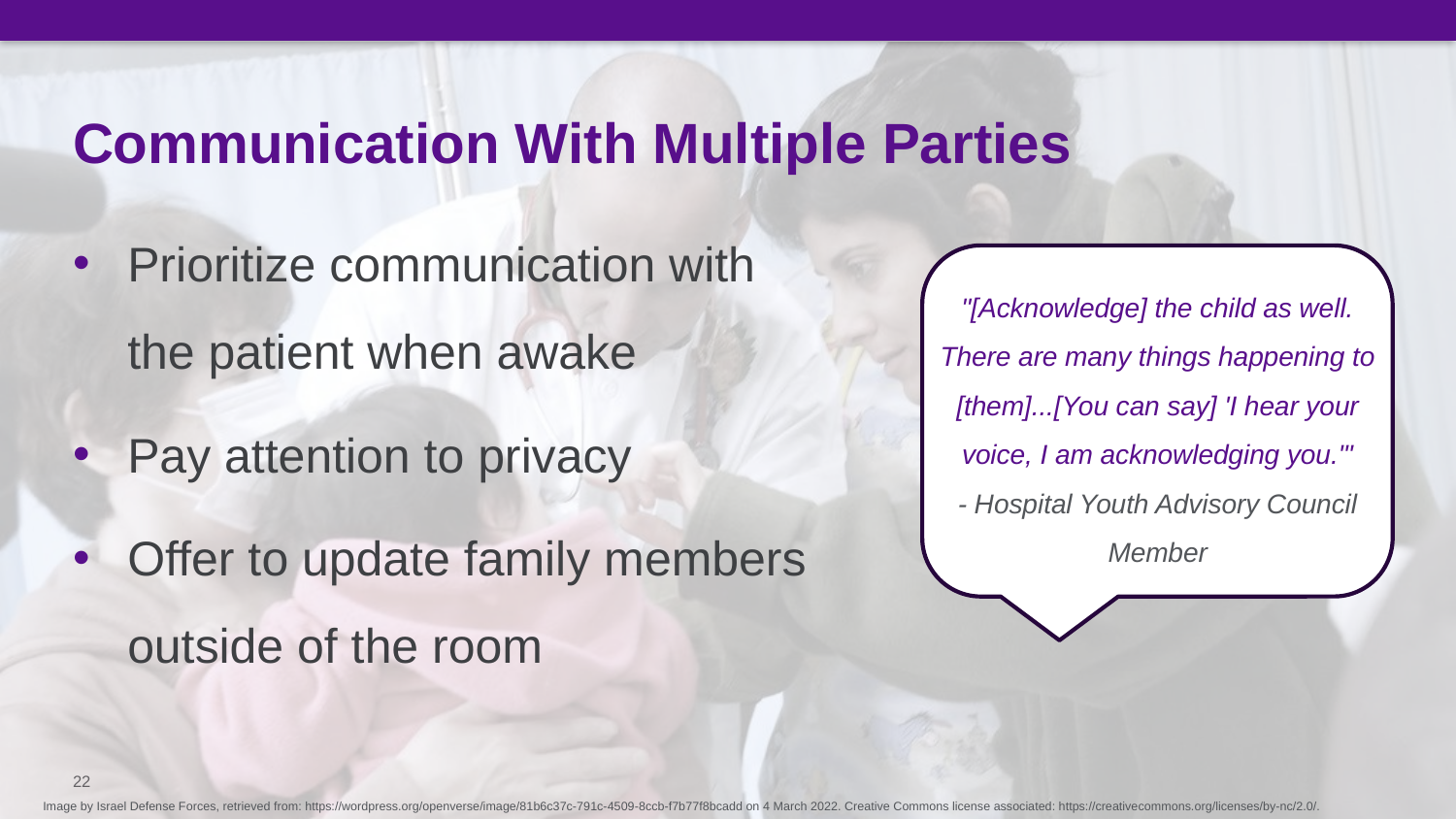

# Communication With Multiple Parties
Prioritize communication with the patient when awake
Pay attention to privacy
Offer to update family members outside of the room
"[Acknowledge] the child as well. There are many things happening to [them]...[You can say] 'I hear your voice, I am acknowledging you.'"
- Hospital Youth Advisory Council Member
22
Image by Israel Defense Forces, retrieved from: https://wordpress.org/openverse/image/81b6c37c-791c-4509-8ccb-f7b77f8bcadd on 4 March 2022. Creative Commons license associated: https://creativecommons.org/licenses/by-nc/2.0/.

## Slide 23
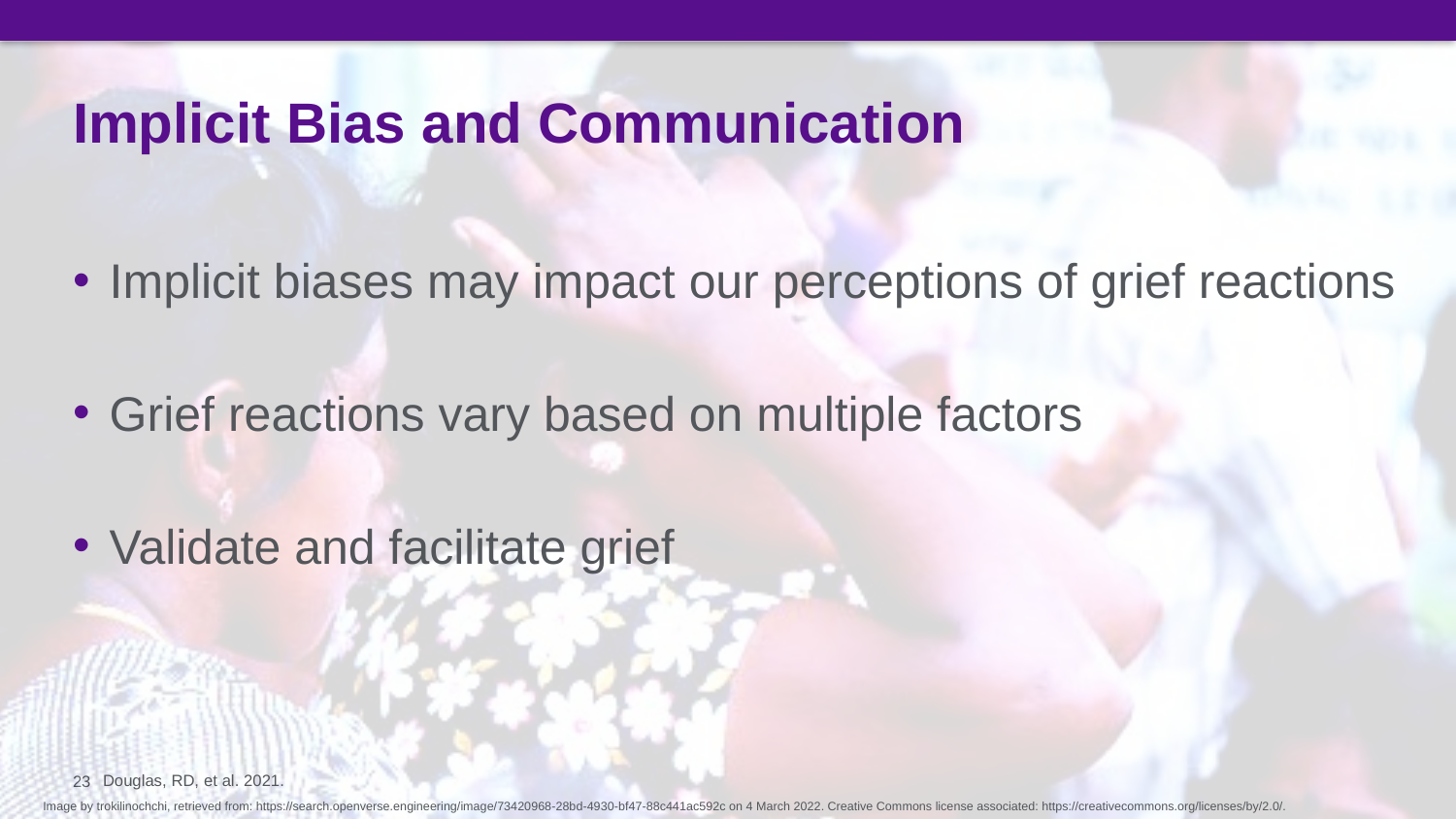

# Implicit Bias and Communication
Implicit biases may impact our perceptions of grief reactions
Grief reactions vary based on multiple factors
Validate and facilitate grief
Douglas, RD, et al. 2021.
23
Image by trokilinochchi, retrieved from: https://search.openverse.engineering/image/73420968-28bd-4930-bf47-88c441ac592c on 4 March 2022. Creative Commons license associated: https://creativecommons.org/licenses/by/2.0/.

## Slide 24
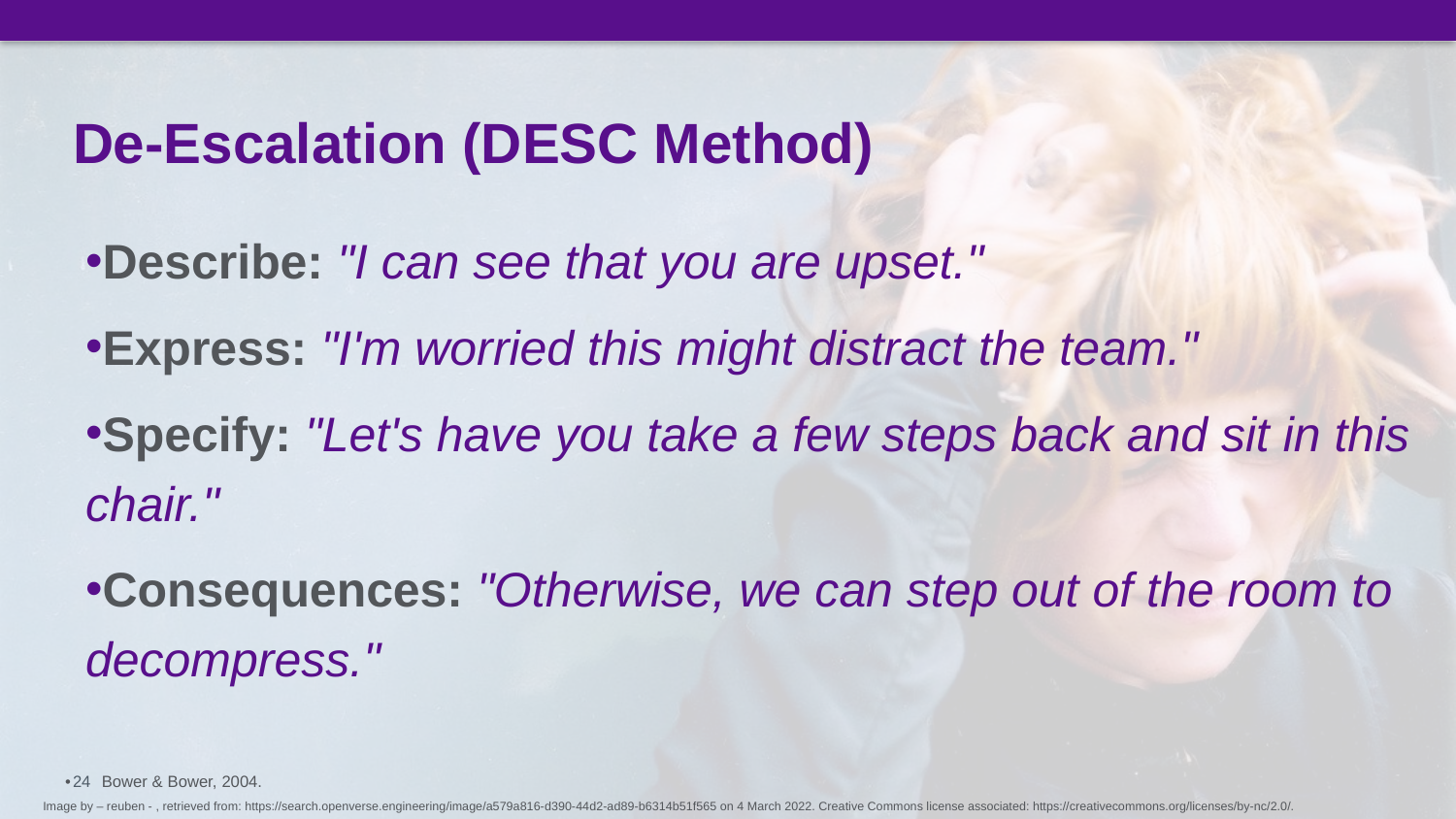

# De-Escalation (DESC Method)
Describe: "I can see that you are upset."
Express: "I'm worried this might distract the team."
Specify: "Let's have you take a few steps back and sit in this chair."
Consequences: "Otherwise, we can step out of the room to decompress."
24
Bower & Bower, 2004.
Image by – reuben - , retrieved from: https://search.openverse.engineering/image/a579a816-d390-44d2-ad89-b6314b51f565 on 4 March 2022. Creative Commons license associated: https://creativecommons.org/licenses/by-nc/2.0/.

## Slide 25
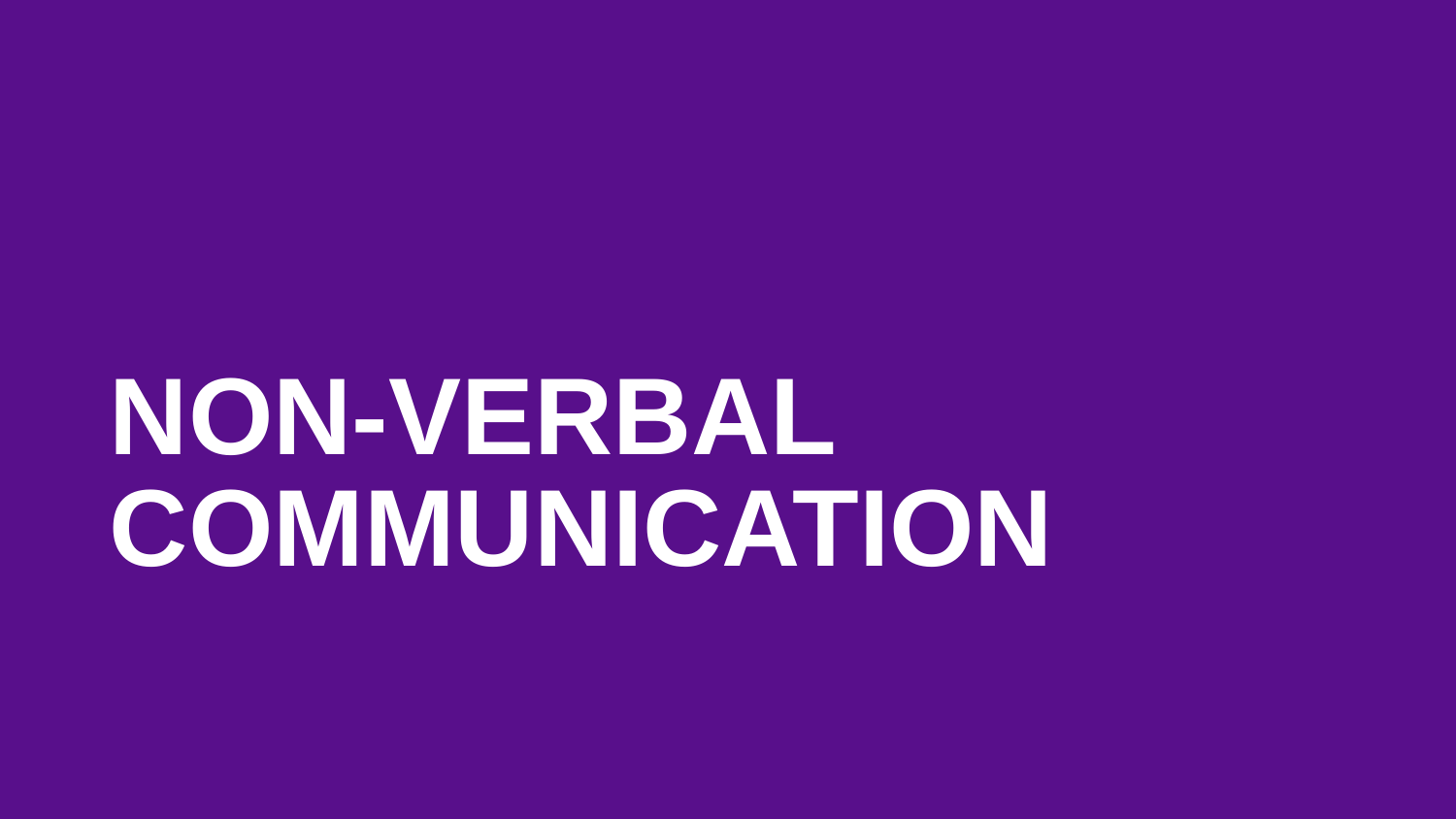

# Non-verbal communication

## Slide 26
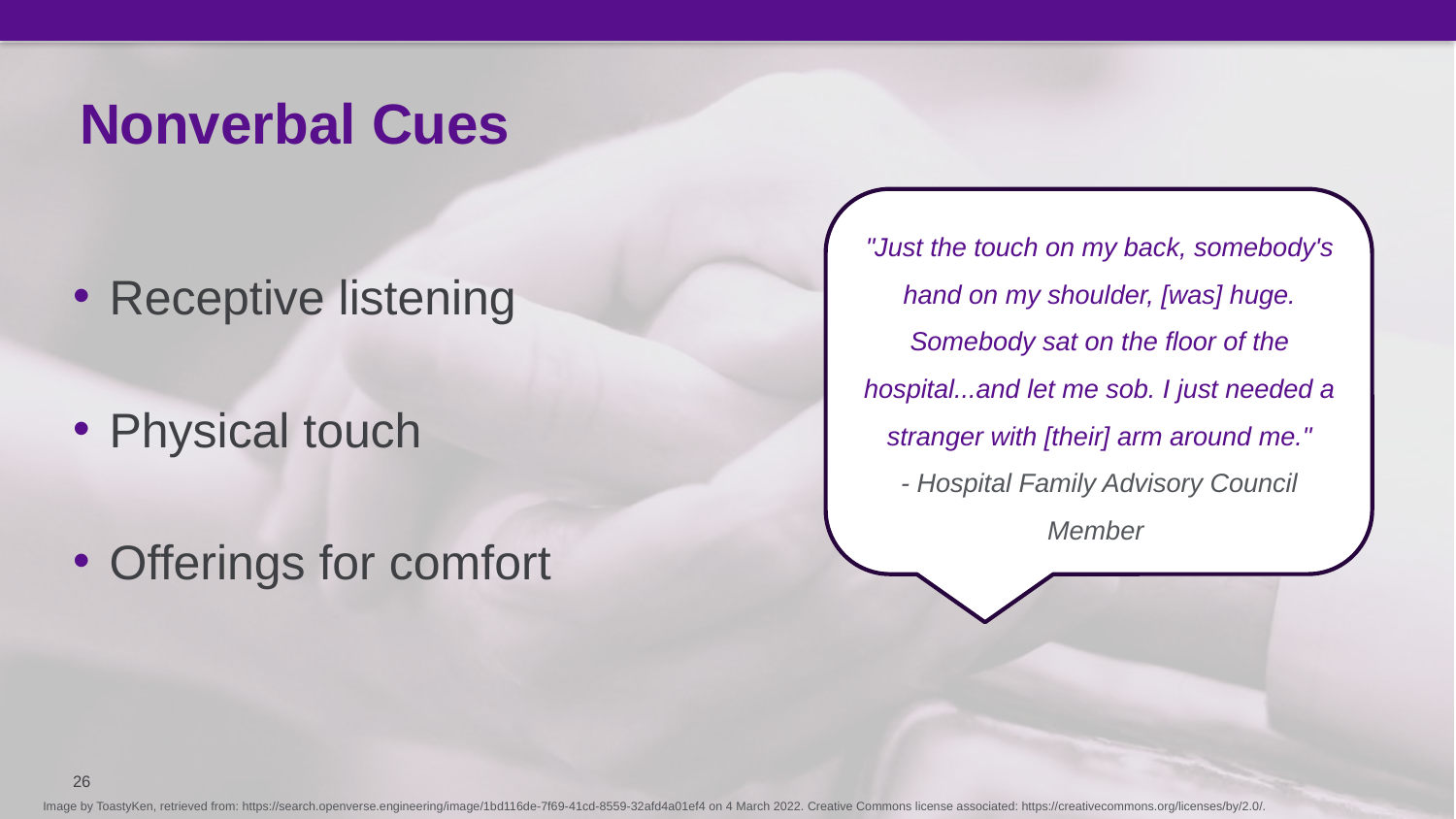

# Nonverbal Cues
"Just the touch on my back, somebody's hand on my shoulder, [was] huge. Somebody sat on the floor of the hospital...and let me sob. I just needed a stranger with [their] arm around me."
- Hospital Family Advisory Council Member
Receptive listening
Physical touch
Offerings for comfort
26
Image by ToastyKen, retrieved from: https://search.openverse.engineering/image/1bd116de-7f69-41cd-8559-32afd4a01ef4 on 4 March 2022. Creative Commons license associated: https://creativecommons.org/licenses/by/2.0/.

## Slide 27
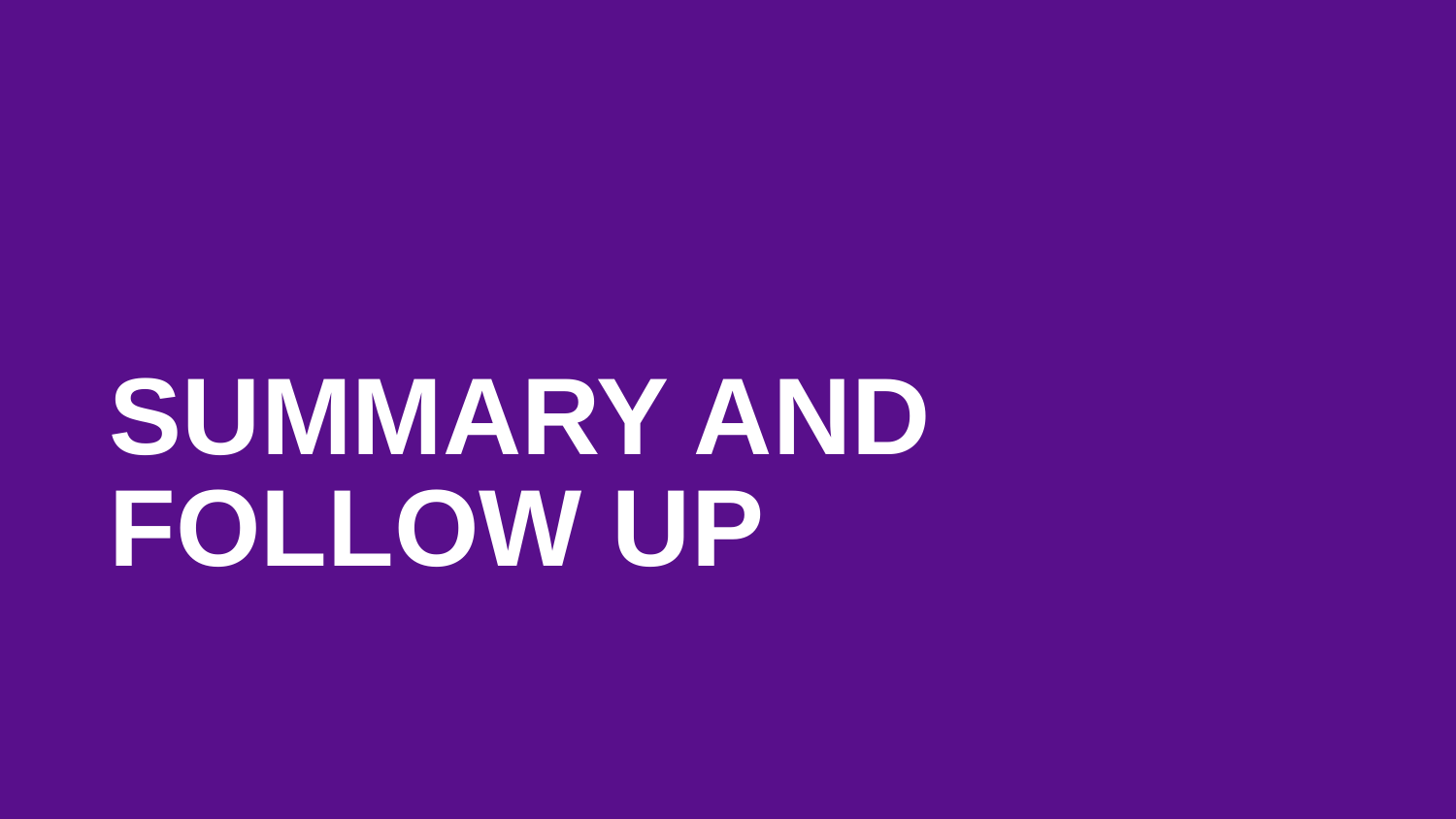

# Summary and Follow up

## Slide 28
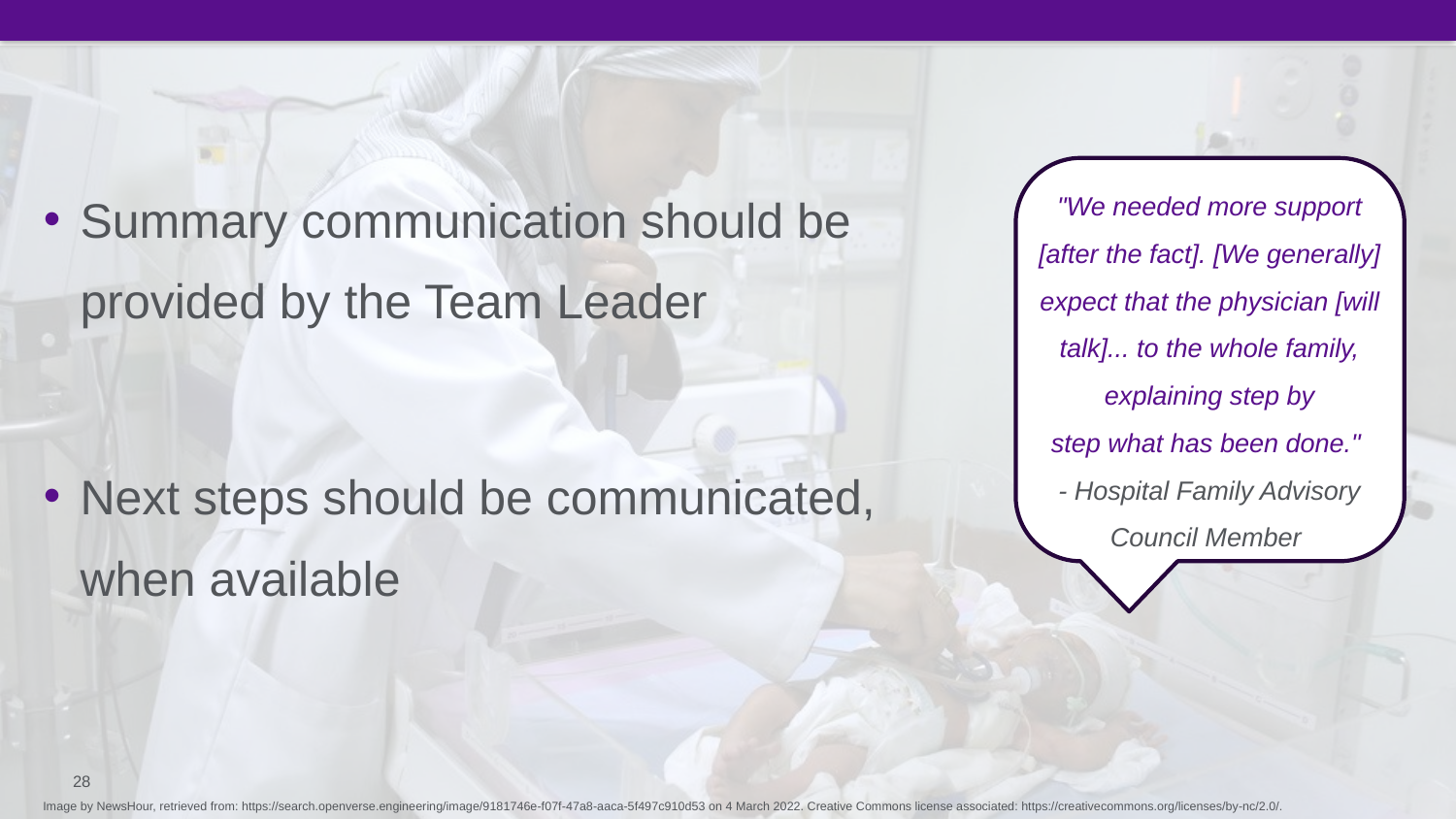

Summary communication should be provided by the Team Leader
Next steps should be communicated, when available
"We needed more support [after the fact]. [We generally] expect that the physician [will talk]... to the whole family, explaining step by step what has been done."
- Hospital Family Advisory Council Member
28
Image by NewsHour, retrieved from: https://search.openverse.engineering/image/9181746e-f07f-47a8-aaca-5f497c910d53 on 4 March 2022. Creative Commons license associated: https://creativecommons.org/licenses/by-nc/2.0/.

## Slide 29
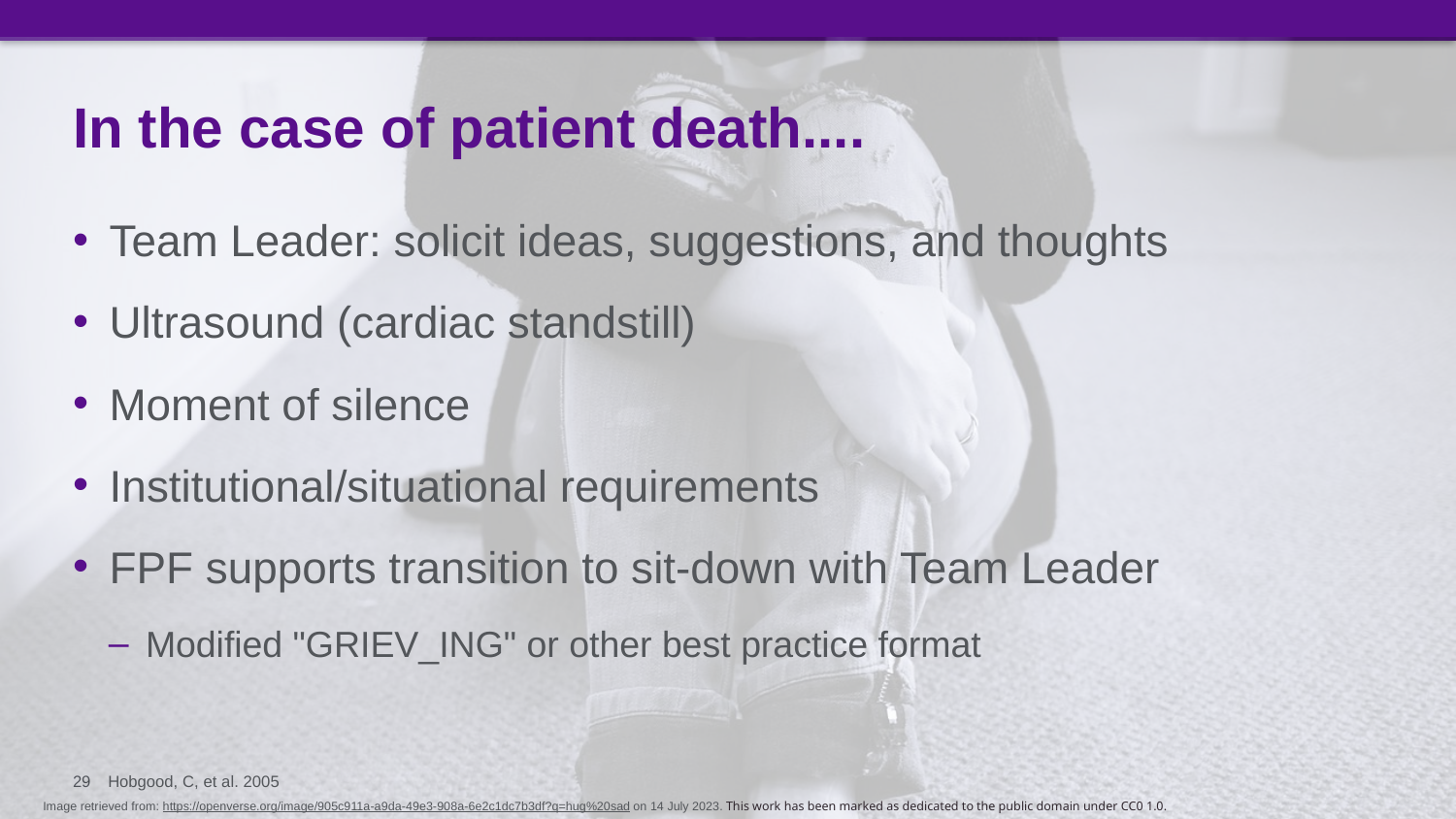

# In the case of patient death....
Team Leader: solicit ideas, suggestions, and thoughts
Ultrasound (cardiac standstill)
Moment of silence
Institutional/situational requirements
FPF supports transition to sit-down with Team Leader
Modified "GRIEV_ING" or other best practice format
29
Hobgood, C, et al. 2005
Image retrieved from: https://openverse.org/image/905c911a-a9da-49e3-908a-6e2c1dc7b3df?q=hug%20sad on 14 July 2023. This work has been marked as dedicated to the public domain under CC0 1.0.

## Slide 30
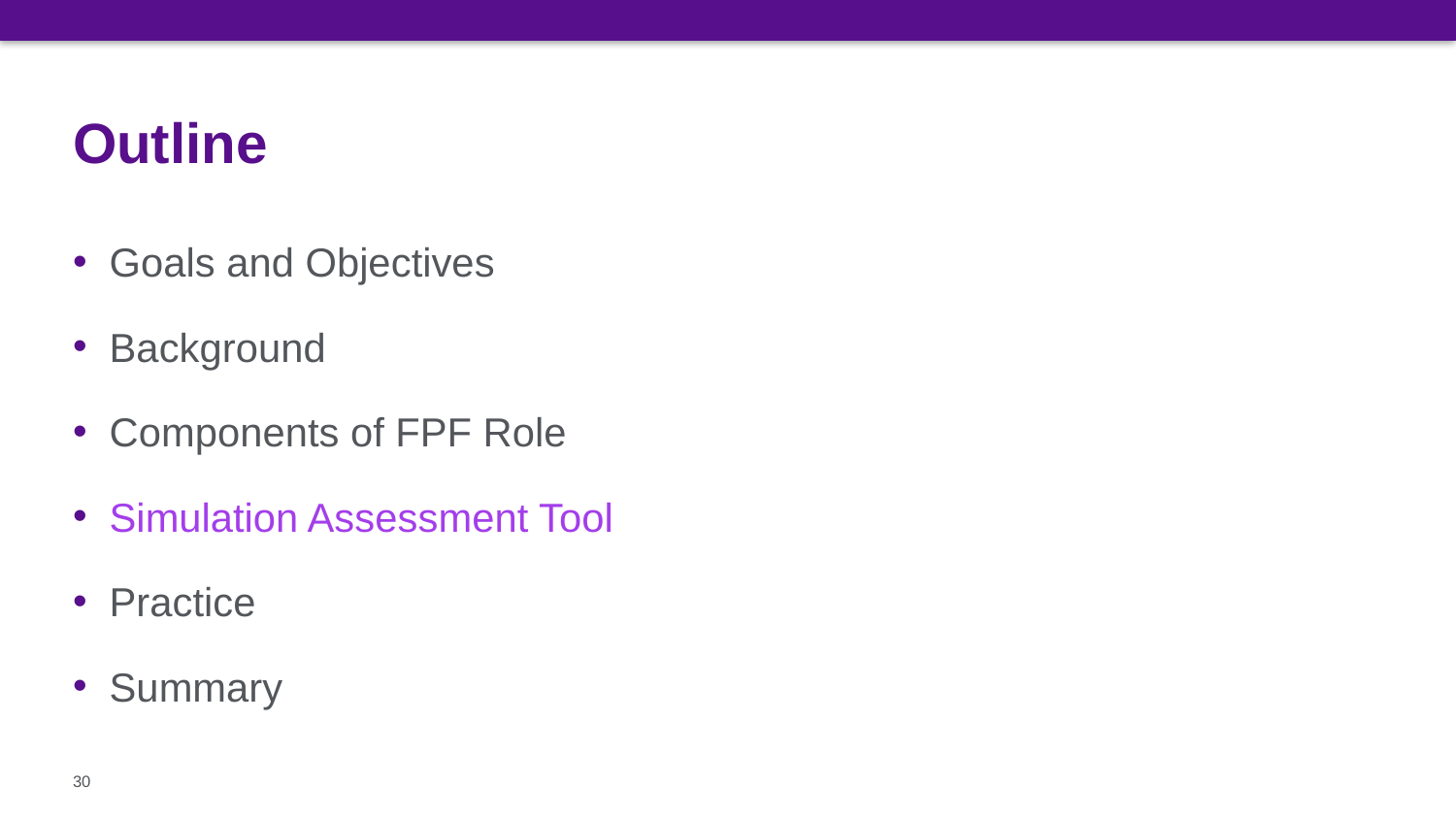

# Outline
Goals and Objectives
Background
Components of FPF Role
Simulation Assessment Tool
Practice
Summary
30

## Slide 31
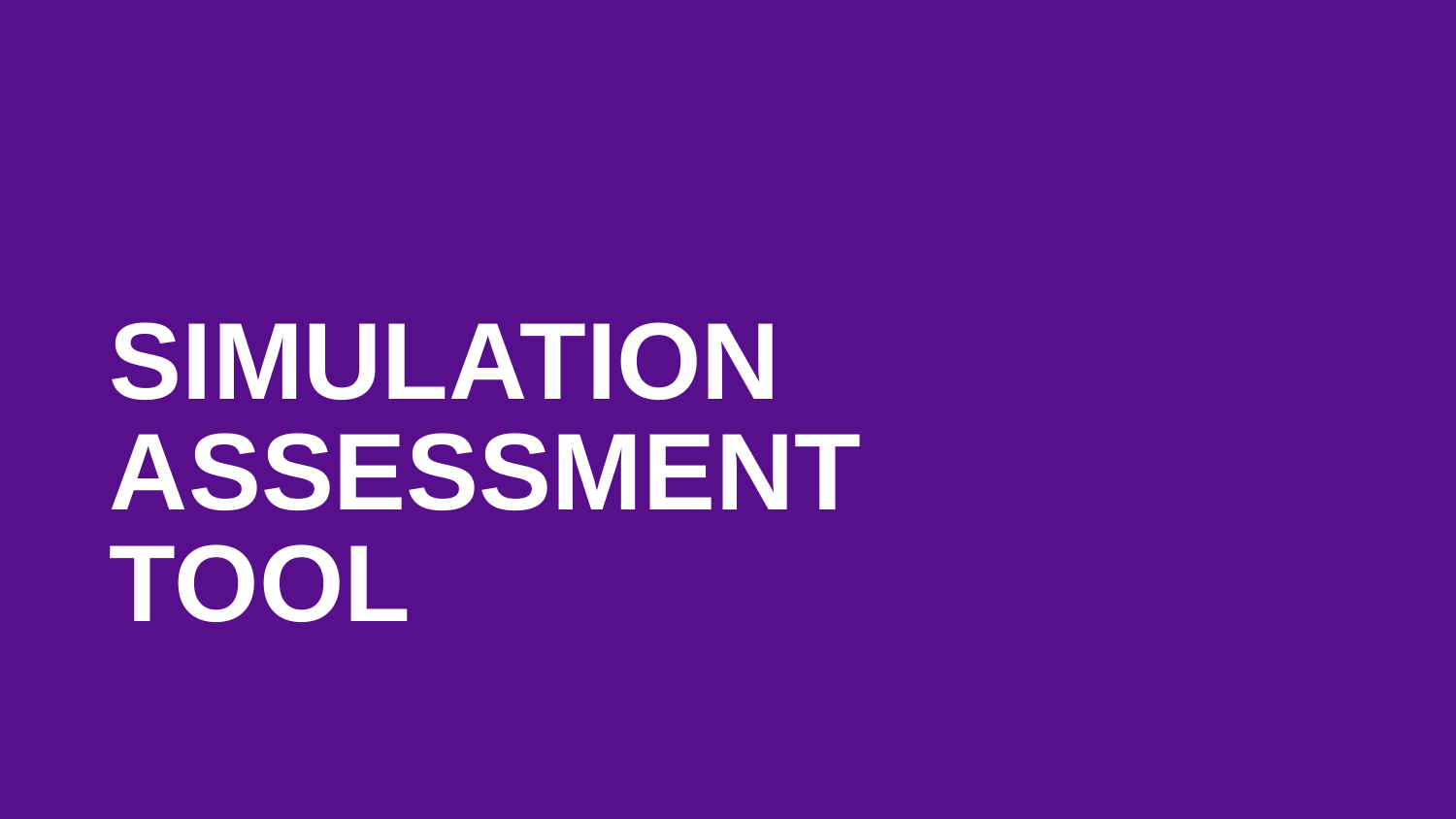

# Simulation assessment TOOL

## Slide 32
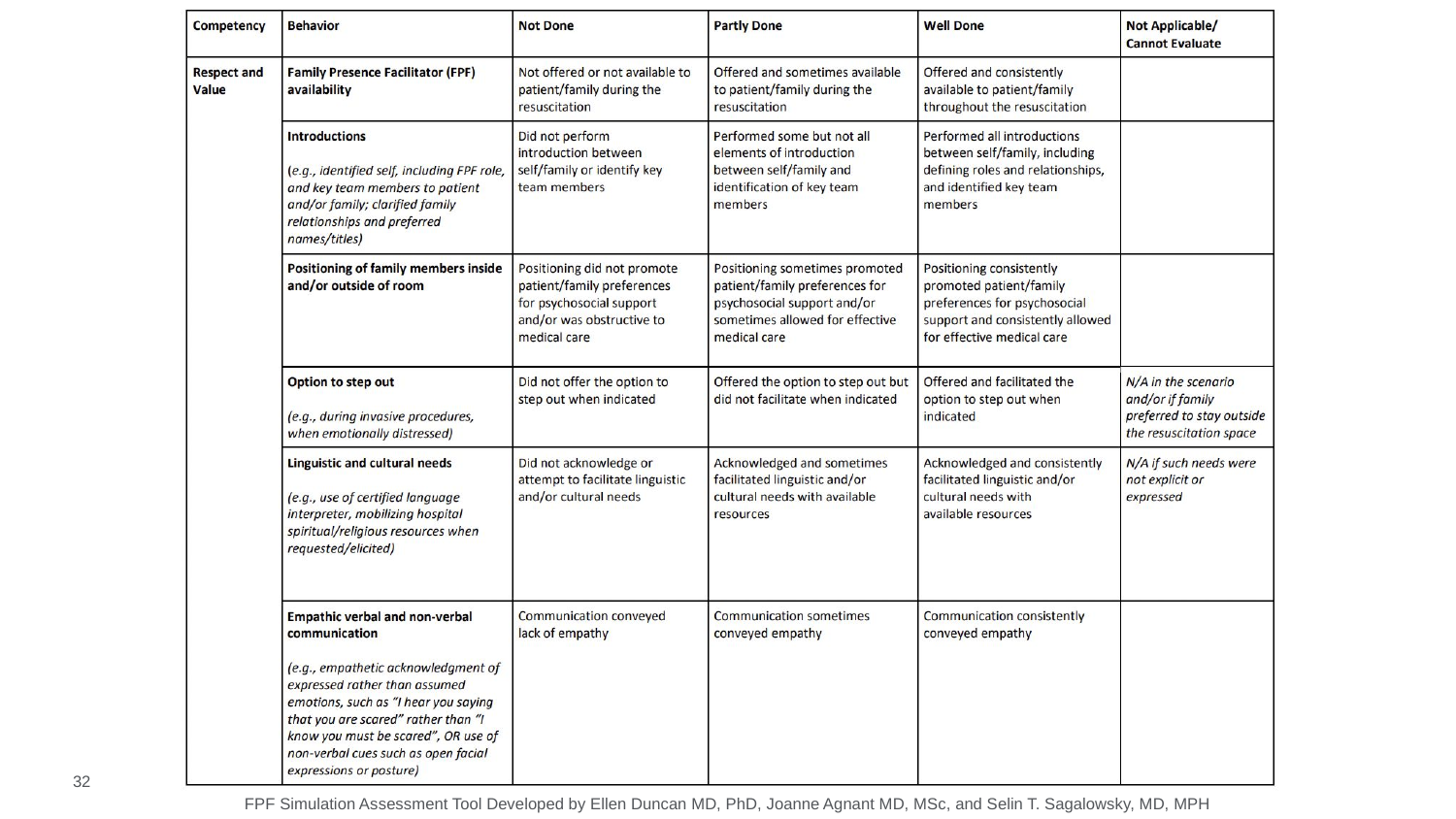

32
FPF Simulation Assessment Tool Developed by Ellen Duncan MD, PhD, Joanne Agnant MD, MSc, and Selin T. Sagalowsky, MD, MPH

## Slide 33
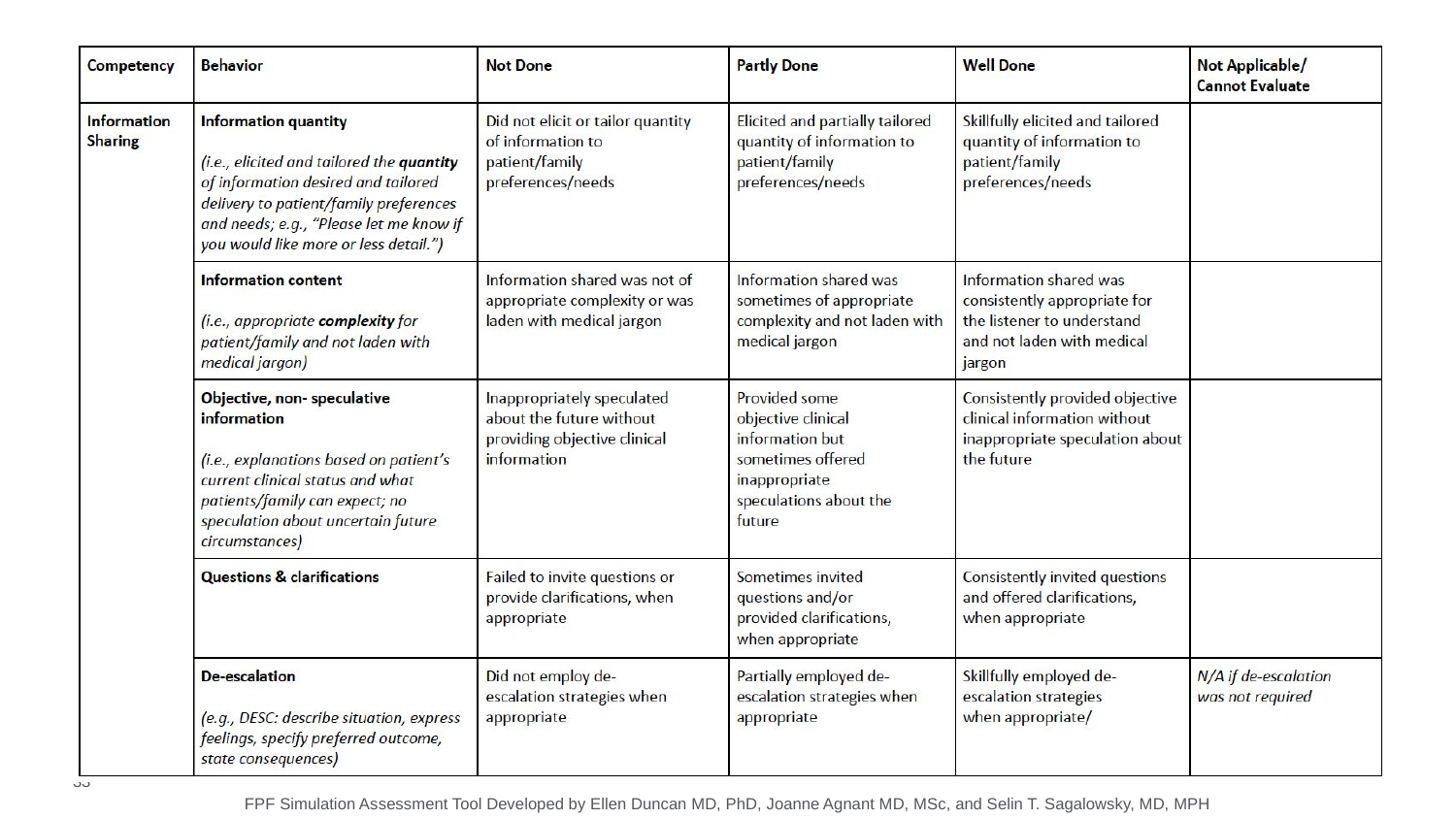

33
FPF Simulation Assessment Tool Developed by Ellen Duncan MD, PhD, Joanne Agnant MD, MSc, and Selin T. Sagalowsky, MD, MPH

## Slide 34
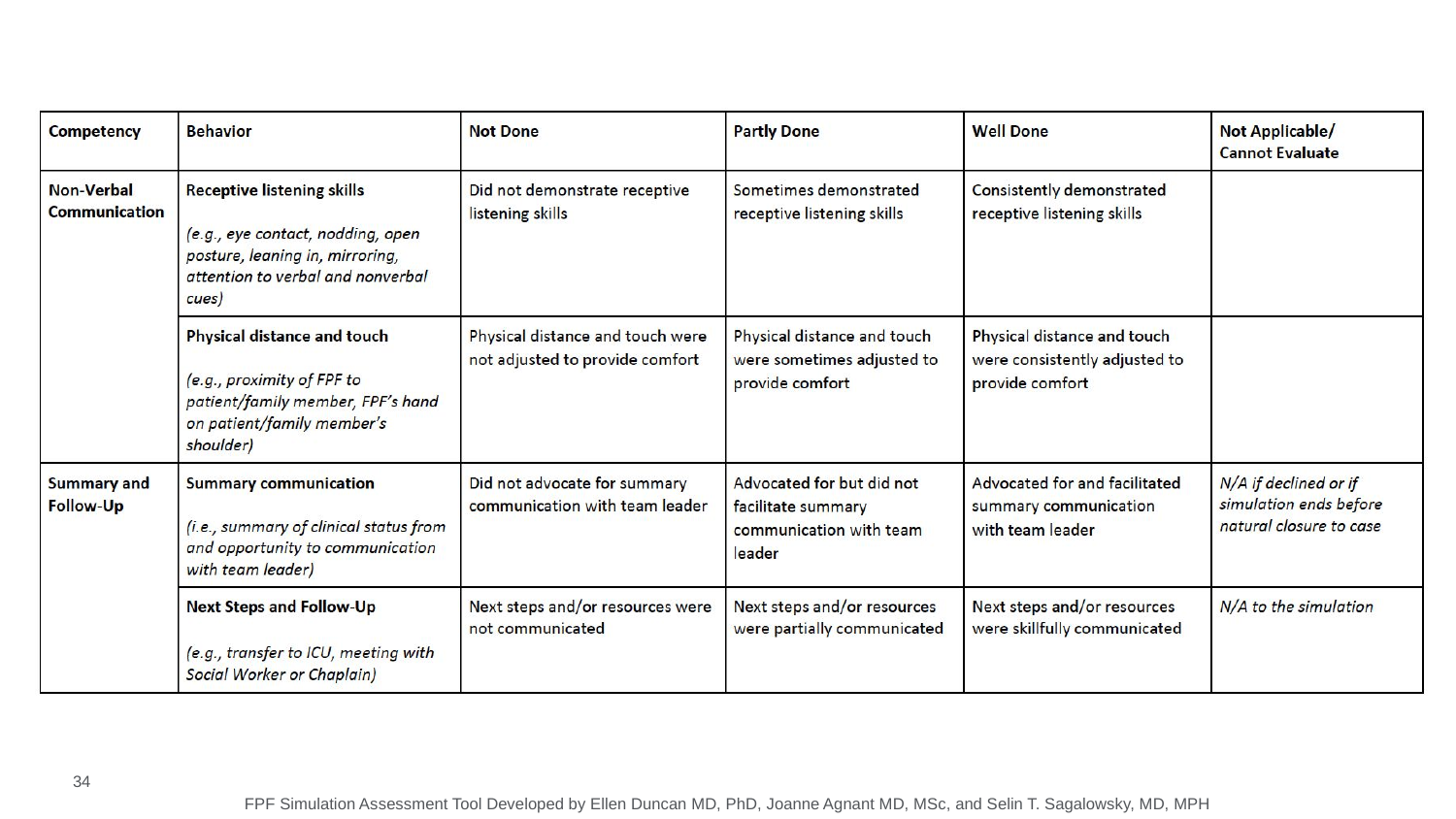

34
FPF Simulation Assessment Tool Developed by Ellen Duncan MD, PhD, Joanne Agnant MD, MSc, and Selin T. Sagalowsky, MD, MPH

## Slide 35
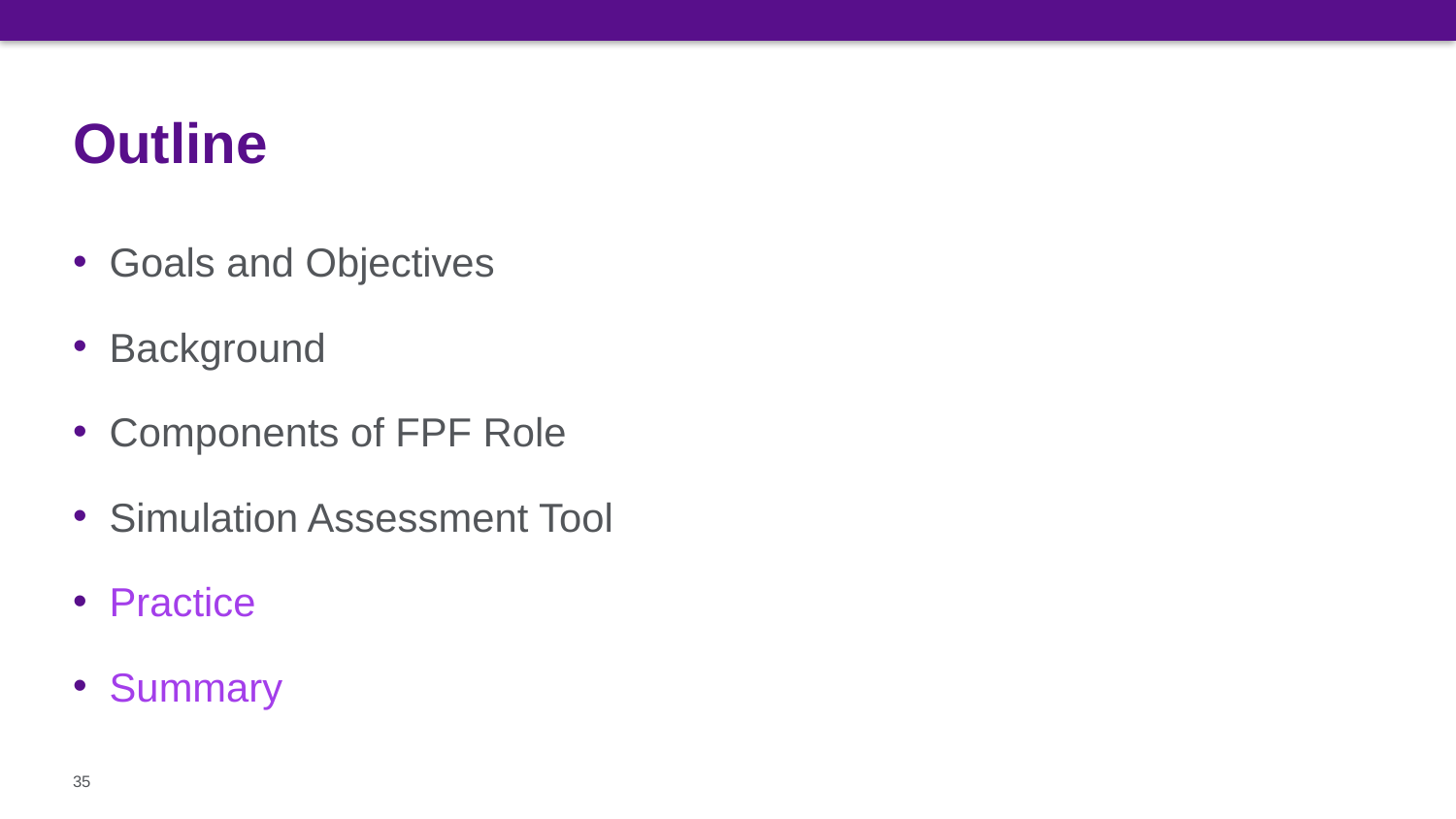

# Outline
Goals and Objectives
Background
Components of FPF Role
Simulation Assessment Tool
Practice
Summary
35

## Slide 36
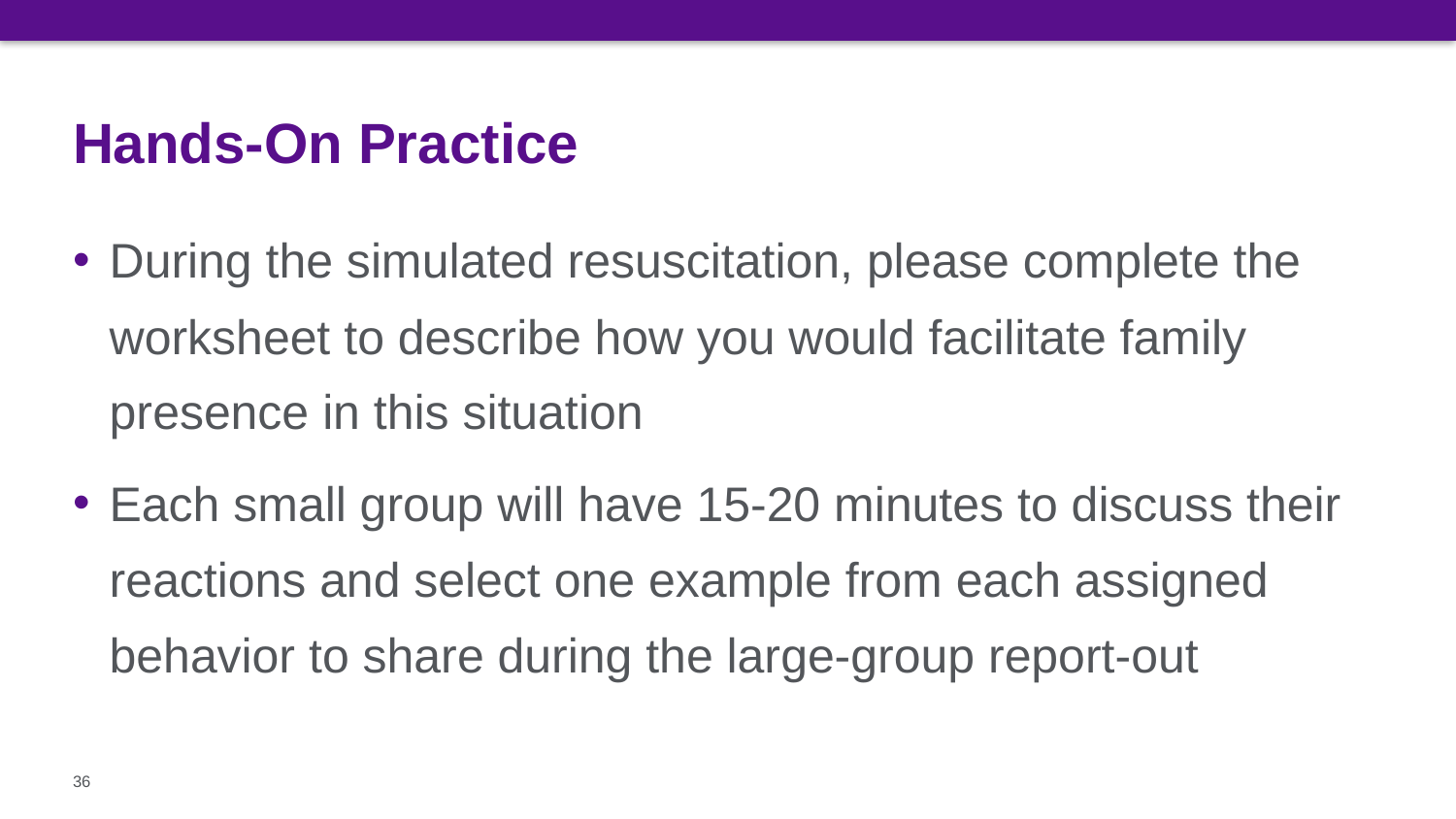

# Hands-On Practice
During the simulated resuscitation, please complete the worksheet to describe how you would facilitate family presence in this situation
Each small group will have 15-20 minutes to discuss their reactions and select one example from each assigned behavior to share during the large-group report-out
36

## Slide 37
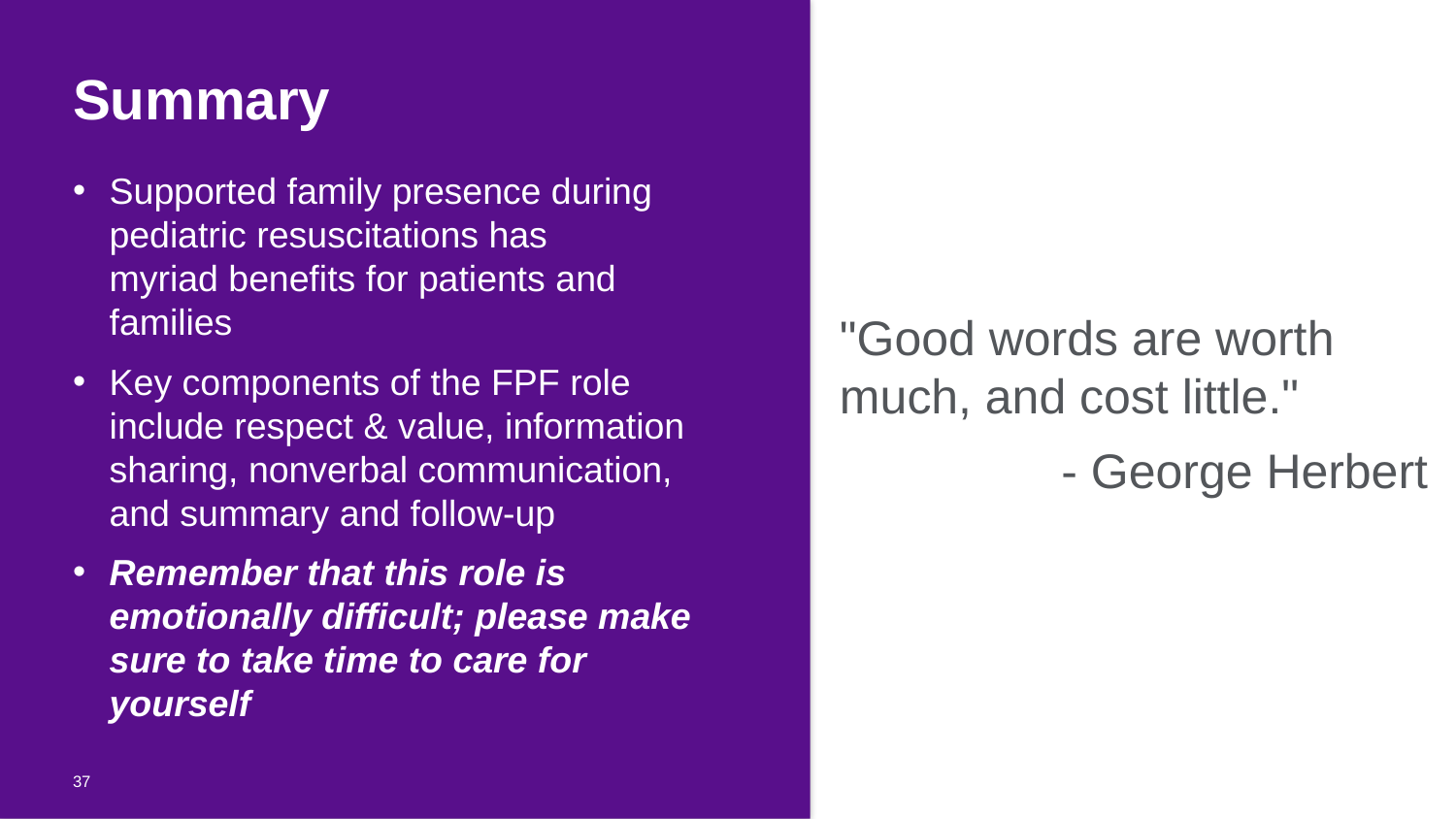

# Summary
Supported family presence during pediatric resuscitations has myriad benefits for patients and families
Key components of the FPF role include respect & value, information sharing, nonverbal communication, and summary and follow-up
Remember that this role is emotionally difficult; please make sure to take time to care for yourself
"Good words are worth much, and cost little."
- George Herbert
37

## Slide 38
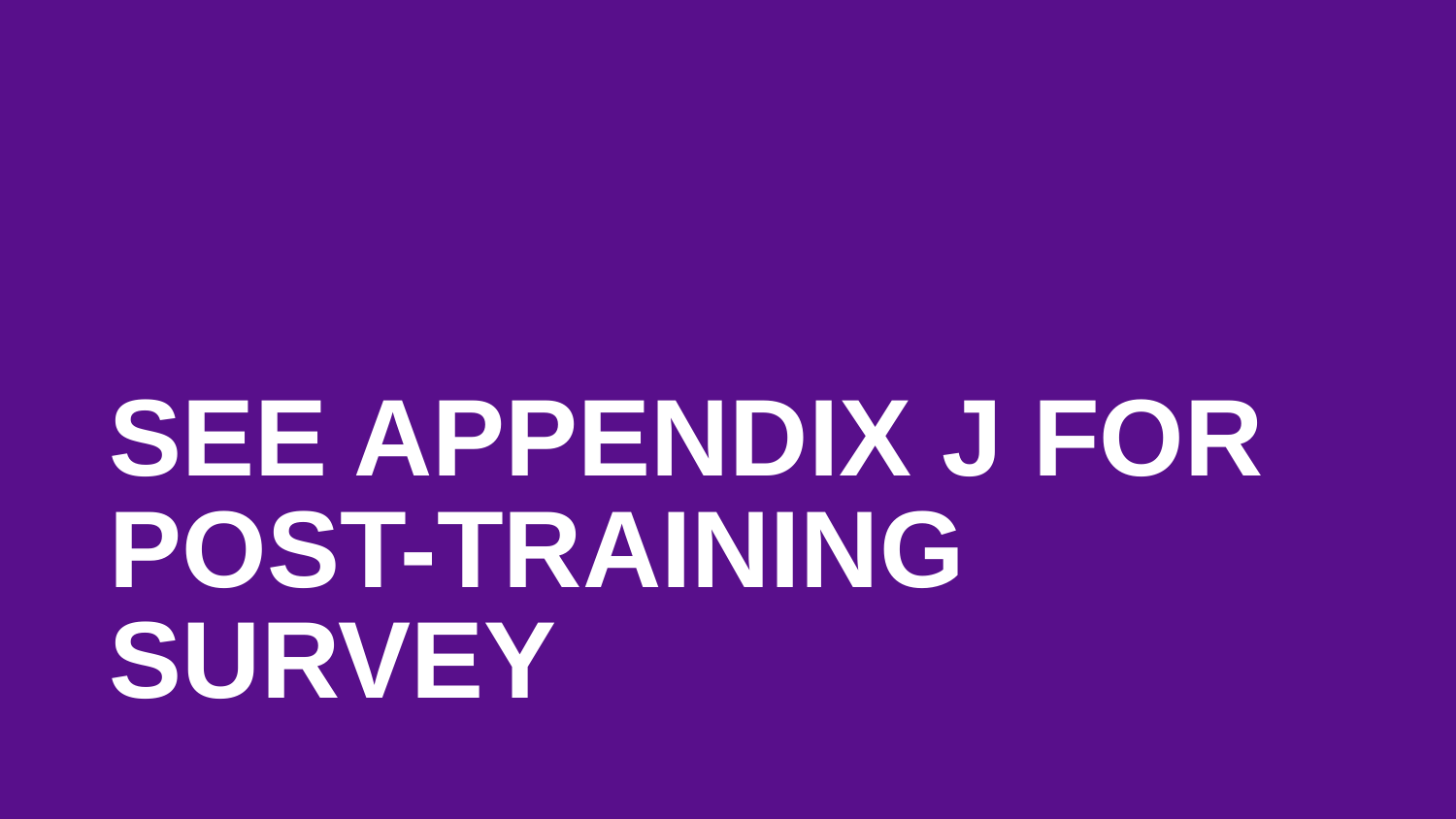

# See appendix J for post-training survey

## Slide 39
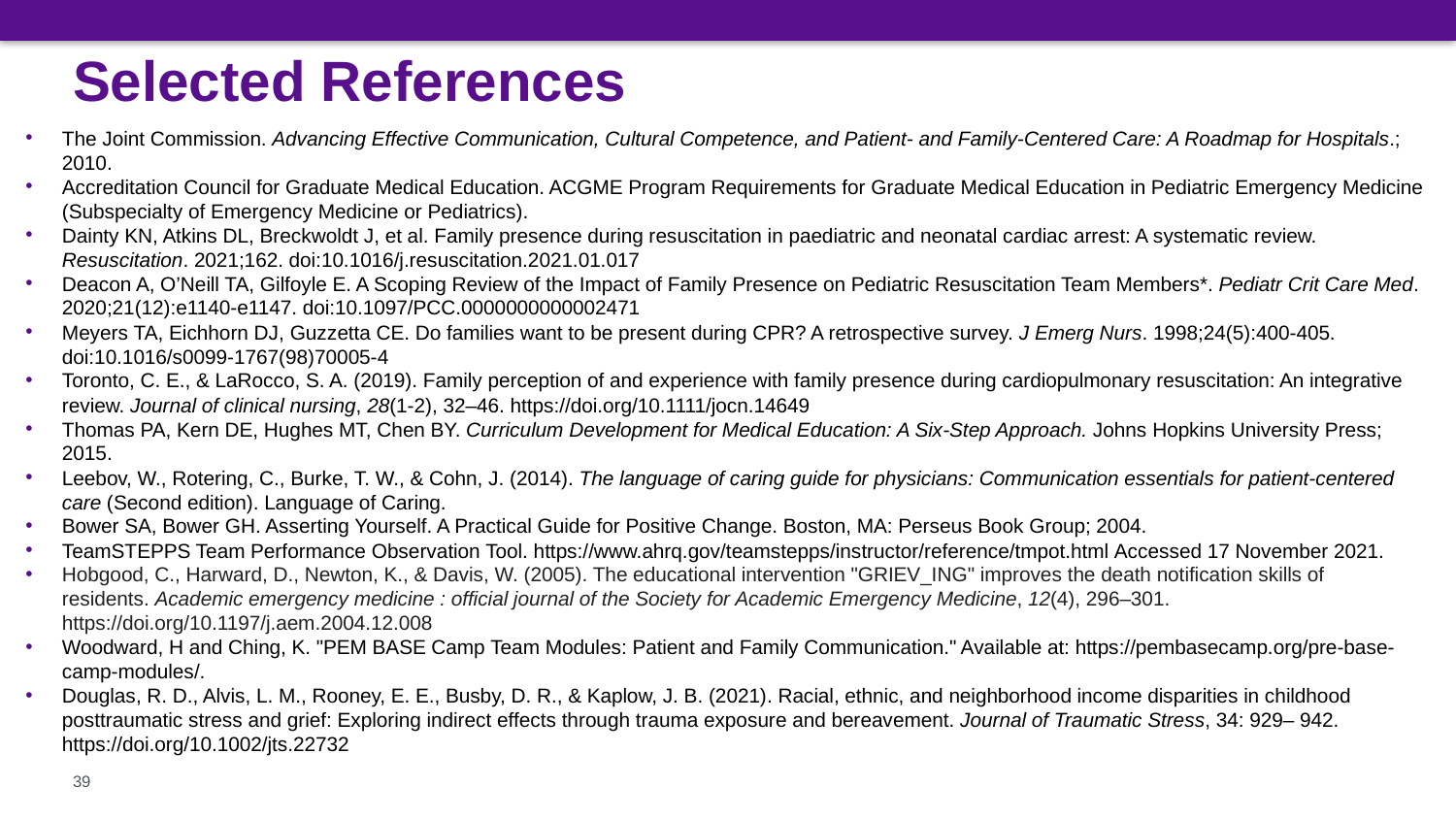

# Selected References
The Joint Commission. Advancing Effective Communication, Cultural Competence, and Patient- and Family-Centered Care: A Roadmap for Hospitals.; 2010.
Accreditation Council for Graduate Medical Education. ACGME Program Requirements for Graduate Medical Education in Pediatric Emergency Medicine (Subspecialty of Emergency Medicine or Pediatrics).
Dainty KN, Atkins DL, Breckwoldt J, et al. Family presence during resuscitation in paediatric and neonatal cardiac arrest: A systematic review. Resuscitation. 2021;162. doi:10.1016/j.resuscitation.2021.01.017
Deacon A, O’Neill TA, Gilfoyle E. A Scoping Review of the Impact of Family Presence on Pediatric Resuscitation Team Members*. Pediatr Crit Care Med. 2020;21(12):e1140-e1147. doi:10.1097/PCC.0000000000002471
Meyers TA, Eichhorn DJ, Guzzetta CE. Do families want to be present during CPR? A retrospective survey. J Emerg Nurs. 1998;24(5):400-405. doi:10.1016/s0099-1767(98)70005-4
Toronto, C. E., & LaRocco, S. A. (2019). Family perception of and experience with family presence during cardiopulmonary resuscitation: An integrative review. Journal of clinical nursing, 28(1-2), 32–46. https://doi.org/10.1111/jocn.14649
Thomas PA, Kern DE, Hughes MT, Chen BY. Curriculum Development for Medical Education: A Six-Step Approach. Johns Hopkins University Press; 2015.
Leebov, W., Rotering, C., Burke, T. W., & Cohn, J. (2014). The language of caring guide for physicians: Communication essentials for patient-centered care (Second edition). Language of Caring.
Bower SA, Bower GH. Asserting Yourself. A Practical Guide for Positive Change. Boston, MA: Perseus Book Group; 2004.
TeamSTEPPS Team Performance Observation Tool. https://www.ahrq.gov/teamstepps/instructor/reference/tmpot.html Accessed 17 November 2021.
Hobgood, C., Harward, D., Newton, K., & Davis, W. (2005). The educational intervention "GRIEV_ING" improves the death notification skills of residents. Academic emergency medicine : official journal of the Society for Academic Emergency Medicine, 12(4), 296–301. https://doi.org/10.1197/j.aem.2004.12.008
Woodward, H and Ching, K. "PEM BASE Camp Team Modules: Patient and Family Communication." Available at: https://pembasecamp.org/pre-base-camp-modules/.
Douglas, R. D., Alvis, L. M., Rooney, E. E., Busby, D. R., & Kaplow, J. B. (2021). Racial, ethnic, and neighborhood income disparities in childhood posttraumatic stress and grief: Exploring indirect effects through trauma exposure and bereavement. Journal of Traumatic Stress, 34: 929– 942. https://doi.org/10.1002/jts.22732
39

## Slide 40
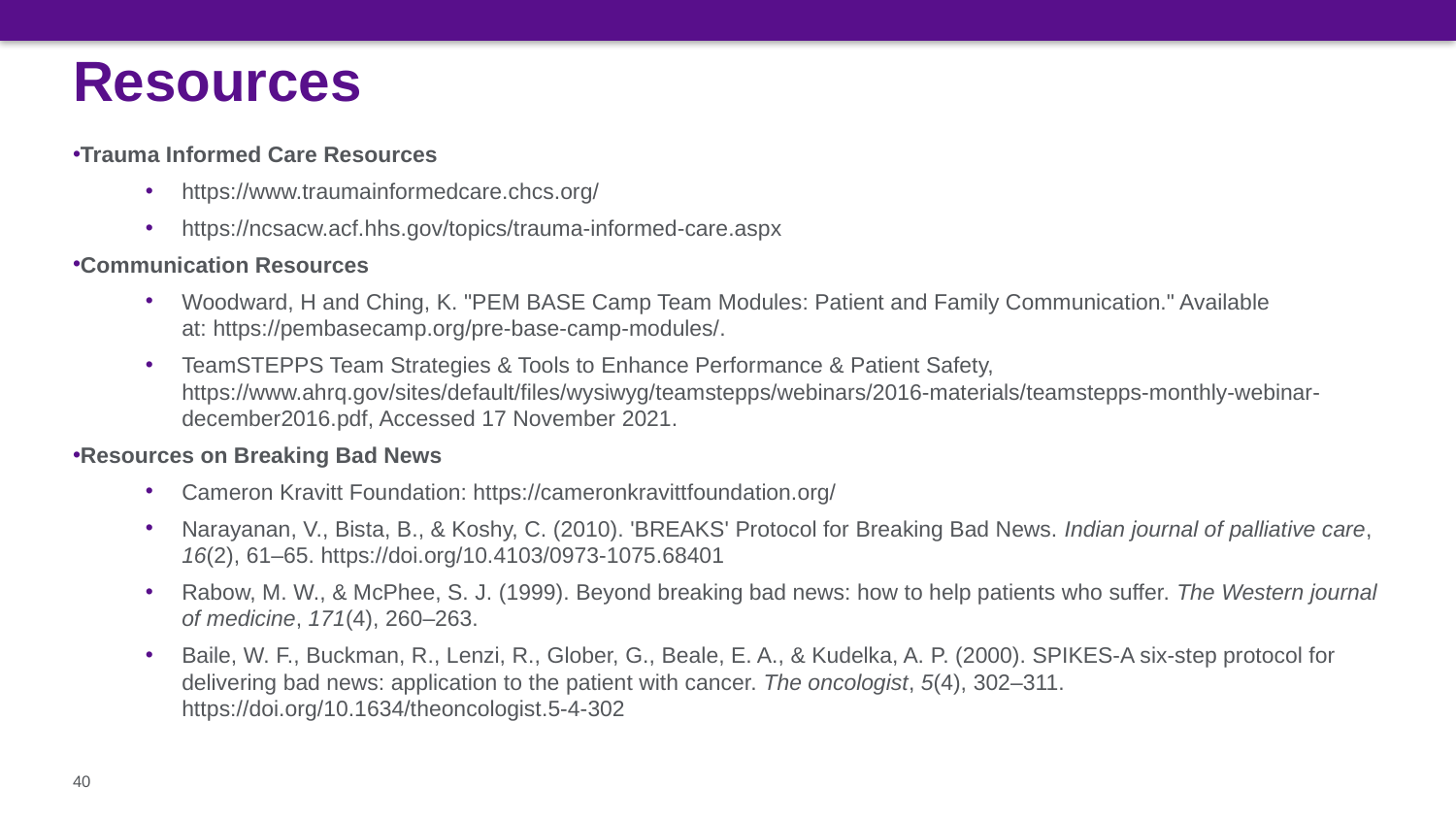

# Resources
Trauma Informed Care Resources
https://www.traumainformedcare.chcs.org/
https://ncsacw.acf.hhs.gov/topics/trauma-informed-care.aspx
Communication Resources
Woodward, H and Ching, K. "PEM BASE Camp Team Modules: Patient and Family Communication." Available at: https://pembasecamp.org/pre-base-camp-modules/.
TeamSTEPPS Team Strategies & Tools to Enhance Performance & Patient Safety, https://www.ahrq.gov/sites/default/files/wysiwyg/teamstepps/webinars/2016-materials/teamstepps-monthly-webinar-december2016.pdf, Accessed 17 November 2021.
Resources on Breaking Bad News
Cameron Kravitt Foundation: https://cameronkravittfoundation.org/
Narayanan, V., Bista, B., & Koshy, C. (2010). 'BREAKS' Protocol for Breaking Bad News. Indian journal of palliative care, 16(2), 61–65. https://doi.org/10.4103/0973-1075.68401
Rabow, M. W., & McPhee, S. J. (1999). Beyond breaking bad news: how to help patients who suffer. The Western journal of medicine, 171(4), 260–263.
Baile, W. F., Buckman, R., Lenzi, R., Glober, G., Beale, E. A., & Kudelka, A. P. (2000). SPIKES-A six-step protocol for delivering bad news: application to the patient with cancer. The oncologist, 5(4), 302–311. https://doi.org/10.1634/theoncologist.5-4-302
40

## Slide 41
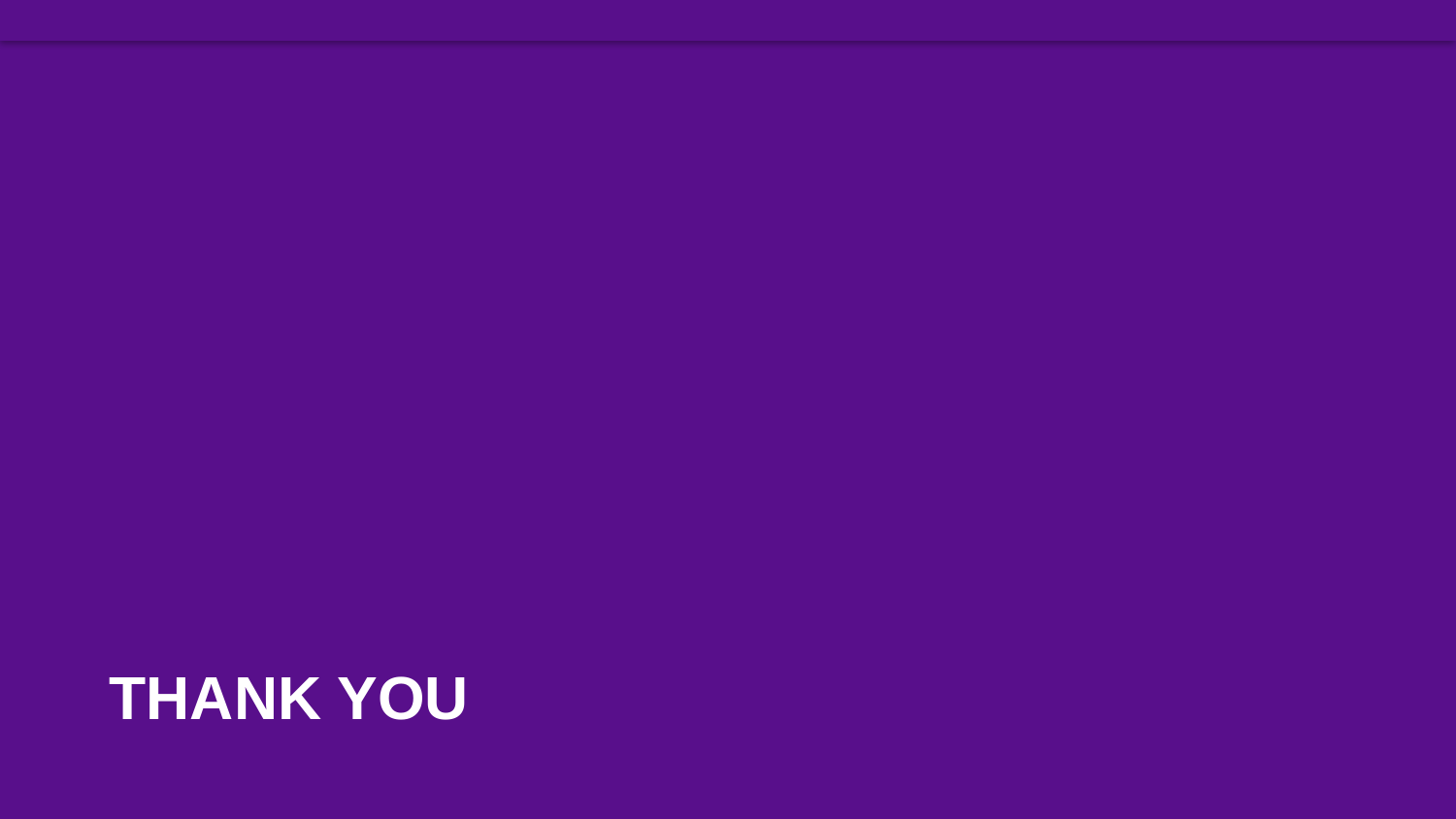

Supplement: Supplementary file 1 — FPF Curriculum.pptxFPF Curriculum Recording.mp4Role-Play Script Without FPF.docxRole-Play Script With FPF.docxFPF Participant Worksheet.docxFPF Instructor Worksheet.docxFPF Survey.docxSP Training.pptxSimulated Participant Training Case.docxFPF-SAT.docx [file mep_2374-8265.11445-s001.zip › A. FPF Curriculum.pptx]
