# Supplementary material for: Development and Implementation of a Family Presence Facilitator Curriculum for Interprofessional Use in Pediatric Medical Resuscitations
Source: MedEdPORTAL. 2024 Oct 8;20:11445. doi: 10.15766/mep_2374-8265.11445 (PMC11458738; doi:10.15766/mep_2374-8265.11445)
Supplement: Supplementary file 1 — FPF Curriculum.pptxFPF Curriculum Recording.mp4Role-Play Script Without FPF.docxRole-Play Script With FPF.docxFPF Participant Worksheet.docxFPF Instructor Worksheet.docxFPF Survey.docxSP Training.pptxSimulated Participant Training Case.docxFPF-SAT.docx [file mep_2374-8265.11445-s001.zip › C. Role-Play Script Without FPF.docx]

**Participants**^[[1]](#footnote-2)^:

Team Leader

Airway Provider

Nurse

Family Member

***Setting****: Pediatric Emergency Department Resuscitation Bay*

*Materials:*

- *Table (for simulated resuscitation)*
- *Infant manikin or low-fidelity doll*
- *Infant bag-valve-mask*
- *Miller 1 laryngoscope, 3.5 (and 3.0) endotracheal tubes*
- *IV taped to manikin arm*
- *2 empty 3-5cc syringes to simulate medication administration*
- *Small blanket (for shoulder roll)*

**Background**: *Junior, a 6-month-old boy in the Pediatric Emergency Department, is being resuscitated in the setting of pneumonia and respiratory failure*

**Team leader**: Okay, everybody, we have a 6-month-old boy presenting in hypotensive septic shock and respiratory failure in the setting of pneumonia. We have given 40 mL/kg of normal saline and have ordered a norepinephrine drip for refractory hypotension. We have also given vancomycin and ceftriaxone. We are currently bag-mask ventilating the patient and are preparing for intubation. We are using ketamine and rocuronium for intubation.

**Family Member** [*to team*]: What’s going on? What is that thing over his mouth? Please tell me what’s happening to Junior!

**Team Leader:** Mom, we’re glad you're here with us, and I’m sorry that we don't have any available staff members to be with you at this moment. Right now, we need to focus on treating your child, but we will update you as soon as possible. Please feel free to sit down or step away if you would like to.

**Family Member** [*to team*]: What is happening? Is he not breathing? Is he going to be okay?!

**Nurse:** I will have someone come talk to you as soon as I can. Doctor, I have the RSI medications prepared. Shall we go through our intubation checklist?

**Team Leader**: Yes, let’s do that. Dr. Y, can you please tell me what we have available?

**Airway Provider**: We have suction, a bag-valve-mask, a 3.5 cuffed endotracheal tube with a 3.0 available as well, a Miller 1 blade, and video laryngoscopy as a backup airway modality. We also have in-line end-tidal CO2 monitoring available. We have placed a shoulder roll for the patient, and the bed is at an appropriate height. Nurse, I believe you have ketamine and rocuronium drawn up, is that correct?

**Nurse**: Yes, that is correct.

**Team Leader**: Is everyone ready for intubation?

**Family Member** [*to team*]: Intubation? What is that? What are you going to do to Junior? Is he going to be ok? [*to self*] This is all so overwhelming!

**Team**: [*all together*] Yes.

**Team Leader**: Nurse, please administer the ketamine.

**Nurse**: [*administers ketamine*] I have given the ketamine.

**Team Leader**: Nurse, please give the rocuronium, and we will wait for 45 seconds.

Family Member [*to self*]: Waiting? What are they waiting for?! Junior, Mommy’s here...Mommy’s here...

**Nurse**: [*administers rocuronium*] I have given the rocuronium. [*45 seconds pass*] It has now been 45 seconds since the paralytic was given.

**Team Leader**: Dr. Y, you may proceed with intubation.

**Family Member** [to team]*:* Paralytic? Is he *paralyzed*?? Wait, what are you doing with that tube? I don’t understand what’s happening!

[*Team Is Intubating; Family Member is in a corner, looking distraught (hand over mouth, rocking, crying)*]

1. We recognize that team structures differ by location and/or setting; please modify these roles as necessary to replicate your team [↑](#footnote-ref-2)
