# Supplementary material for: Development and Implementation of a Family Presence Facilitator Curriculum for Interprofessional Use in Pediatric Medical Resuscitations
Source: MedEdPORTAL. 2024 Oct 8;20:11445. doi: 10.15766/mep_2374-8265.11445 (PMC11458738; doi:10.15766/mep_2374-8265.11445)
Supplement: Supplementary file 1 — FPF Curriculum.pptxFPF Curriculum Recording.mp4Role-Play Script Without FPF.docxRole-Play Script With FPF.docxFPF Participant Worksheet.docxFPF Instructor Worksheet.docxFPF Survey.docxSP Training.pptxSimulated Participant Training Case.docxFPF-SAT.docx [file mep_2374-8265.11445-s001.zip › D. Role-Play Script With FPF.docx]

**Participants^[[1]](#footnote-2)^:**

Team Leader

Airway Provider

Nurse

Family Member

Family Presence Facilitator

***Setting****: Pediatric Emergency Department Resuscitation Bay*

*Materials:*

- *Table (for simulated resuscitation)*
- *Infant manikin or low-fidelity doll*
- *Infant bag-valve-mask*
- *Miller 1 laryngoscope, 3.5 (and 3.0) endotracheal tubes*
- *IV taped to manikin arm*
- *2 empty 3-5cc syringes to simulate medication administration*
- *Small blanket (for shoulder roll)*

**Background**: *Junior, a 6-month-old boy in the Pediatric Emergency Department, is being resuscitated in the setting of pneumonia and respiratory failure*

**Team leader**: Okay, everybody, we have a 6-month-old boy presenting in hypotensive septic shock and respiratory failure in the setting of pneumonia. We have given 40 mL/kg of normal saline and have ordered a norepinephrine drip for refractory hypotension. We have also given vancomycin and ceftriaxone. We are currently bag-mask ventilating the patient and are preparing for intubation. We are using ketamine and rocuronium for intubation.

**Family Member:** What’s going on? What is that thing over his mouth? Please tell me what’s happening to Junior!

**Team Leader**: My name is Dr. X and I am leading the team taking care of your child. I would like to focus my attention on Junior right now, but I will speak to you as soon as I can.

**FPF** [*approaching patient’s mother, who is at the bedside*]: Are you Junior’s mother? My name is Dr. Z, and I am one of the resident doctors on the team. I am here to support you while the team takes care of your son. Let’s take one step back to give the other doctors and nurses a little more space to care for your child. Then I can give you some information about what is happening right now.

**Family Member:** Ok.

**FPF:** As you can see, there is a lot going on right now for Junior. Dr. X at the foot of the bed is our supervising doctor, and Dr. Y is at the head of the bed. His primary nurse is to his right. Additional team members will arrive shortly to assist.

**Family Member**: Can you please tell me what’s happening to Junior? He has had a cough but seemed fine otherwise until today. I don't understand why he’s struggling so hard to breathe!

**FPF:** Yes, I can hear your concern, and the team is responding to Junior’s difficulty breathing. I can tell you more about the equipment and support they are using for Junior, if that is helpful to you? [*pause*]

**Family Member:** Yes [*or head nod*]

**FPF:** The mask that Dr. Y is using on Junior’s face is helping give his body oxygen since he’s having trouble breathing on his own. Because his body is working so hard to breathe, they are also preparing to give him a breathing tube that will be inserted through his mouth. This will make sure he continues to get the oxygen he needs.

**Family Member**: Is he not breathing at all? Is he going to be okay?!

**FPF** [*Placing hand on shoulder*]: I hear your concerns. Right now, we can see that Junior has a strong heartbeat and the team is giving him oxygen. You did the right thing by bringing him into the emergency room.

**Family Member***:* Why did this happen? What went wrong? What is the team doing now?

**FPF:** These are important questions, and I am going to try to answer as many as I can and help you remember the others so you can address them with Dr. X. Right now, he has a strong heartbeat, and the team is giving him oxygen and preparing to place a breathing tube into his windpipe to help him breathe. This is something we do commonly for children with severe difficulty breathing. First, the team will give medications to sedate him. This will make his body still and comfortable so he can tolerate the breathing tube. [*pause*] The procedure is about to happen; would you like to sit down or step outside for the procedure?

**Family Member:** I would like to sit.

**FPF:** (*gets chair.* *crouching to be eye level with mother)*: You are doing such an amazing job being here for your child. Does anyone else know you are here now?

**Family Member**: I should really call my husband. He doesn’t even know...Oh goodness, I don’t even know what to tell him…Do you think Junior can hear me? What’s going to happen next?

**FPF:** I think that it can be helpful for family members to talk to their child, even with the medication he is getting. You can speak from here or we can go together to the foot of the bed once the breathing tube is secured.

**Family Member:** I would like to stay here.

**FPF**: Of course. I will be here with you.

*[pause as team performs intubation]*

**FPF:** If you would like, when you are ready, we can reach out to your husband and anyone else you would like to update. I can help you with that phone call. Once Dr. X is ready, she will come and give you an update and discuss next steps for Junior.

1. We recognize that team structures differ by location and/or setting; please modify these roles as necessary to replicate your team [↑](#footnote-ref-2)
