# Supplementary material for: Development and Implementation of a Family Presence Facilitator Curriculum for Interprofessional Use in Pediatric Medical Resuscitations
Source: MedEdPORTAL. 2024 Oct 8;20:11445. doi: 10.15766/mep_2374-8265.11445 (PMC11458738; doi:10.15766/mep_2374-8265.11445)
Supplement: Supplementary file 1 — FPF Curriculum.pptxFPF Curriculum Recording.mp4Role-Play Script Without FPF.docxRole-Play Script With FPF.docxFPF Participant Worksheet.docxFPF Instructor Worksheet.docxFPF Survey.docxSP Training.pptxSimulated Participant Training Case.docxFPF-SAT.docx [file mep_2374-8265.11445-s001.zip › E. FPF Participant Worksheet.docx]

**Instructions**:

You will observe two role-play demonstrations of a pediatric medical resuscitation: the first without a Family Presence Facilitator (FPF) and the second with an FPF present. After the first demonstration, you will break up into small groups and discuss how you would provide support to the family member using specific competencies discussed in the FPF curriculum. Each small group will have 20 minutes to discuss their ideas and to select one example from each assigned behavior to share during the report-out. There will then be a second role-play demonstration, after which each small group will compare the two scenarios, focusing particularly on what went well and on what could be improved. This will be followed by a second report-out.

| **Competency** | **Behavior & Key Components** | **Scenario Prompts** | **Notes** |
| --- | --- | --- | --- |
| **Respect & Value** | **Introductions**  Performed all introductions between self/family, including defining roles and relationships, and identified key team members | At what point in the scenario would you speak up?  What would you say? |  |
| **Respect & Value** | **Positioning of family members inside and/or outside of room**  Positioning consistently promoted patient/family preferences for psychosocial support and allowed for effective medical care | How would you position yourself and the family member in this scenario? |  |
| **Information Sharing** | **Empathetic verbal and non-verbal communication**  Communication consistently conveyed empathy | How would you respond when the family member said, “This is all so overwhelming!” |  |
| **Information Sharing** | **Information quantity**  Skillfully elicited and tailored quantity of information to patient/family preferences/needs | Which elements of the resuscitation would you narrate? |  |
| **Information Sharing** | **Information content**  Information shared was consistently appropriate for the listener to understand and not laden with medical jargon | Provide two examples of how you would phrase explanations of the medical care being provided. |  |
| **Information Sharing** | **Objective, non-speculative information**  Consistently provided objective clinical information without inappropriate speculation about the future | How would you answer the question, “Is he going to be ok?” |  |
| **Information Sharing** | **Questions & clarifications**  Consistently invited questions and offered clarifications, when appropriate | How would you engage the family member to encourage questions/provide clarifications? |  |
| **Summary & Follow-Up** | **Summary communication**  Advocated for and facilitated summary communication with team leader | How would you facilitate a summary from the team leader? |  |
| **Summary & Follow-Up** | **Next steps & follow-up**  Next steps and/or resources were skillfully communicated | How might you discuss the next steps with the family member? |  |
