# Supplementary material for: Development and Implementation of a Family Presence Facilitator Curriculum for Interprofessional Use in Pediatric Medical Resuscitations
Source: MedEdPORTAL. 2024 Oct 8;20:11445. doi: 10.15766/mep_2374-8265.11445 (PMC11458738; doi:10.15766/mep_2374-8265.11445)
Supplement: Supplementary file 1 — FPF Curriculum.pptxFPF Curriculum Recording.mp4Role-Play Script Without FPF.docxRole-Play Script With FPF.docxFPF Participant Worksheet.docxFPF Instructor Worksheet.docxFPF Survey.docxSP Training.pptxSimulated Participant Training Case.docxFPF-SAT.docx [file mep_2374-8265.11445-s001.zip › F. FPF Instructor Worksheet.docx]

**Instructions**:

You will observe two role-play demonstrations of a pediatric medical resuscitation: the first without a Family Presence Facilitator (FPF) and the second with an FPF present. After the first demonstration, you will break up into small groups and discuss how you would provide support to the family member using specific competencies discussed in the FPF curriculum. Each small group will have 20 minutes to discuss their ideas and to select one example from each assigned behavior to share during the report-out. There will then be a second role-play demonstration, after which each small group will compare the two scenarios, focusing particularly on what went well and on what could be improved. This will be followed by a second report-out.

| **Competency** | **Behavior & Key Components** | **Scenario Prompts** | **Notes** |
| --- | --- | --- | --- |
| **Respect & Value** | **Introductions**  Performed all introductions between self/family, including defining roles and relationships, and identified key team members | At what point in the scenario would you speak up?  What would you say? | - Perform introductions as soon as possible:   - e.g., “My name is Dr. Z, and I am one of the resident doctors on the team. Dr. X at the foot of the bed is the supervising doctor. Your child’s nurse is here, along with other members of the team.” - “There is a lot going on right now, and I am here to support you. I want to provide as much information as you would like to have.” |
| **Respect & Value** | **Positioning of family members inside and/or outside of room**  Positioning consistently promoted patient/family preferences for psychosocial support and allowed for effective medical care | How would you position yourself and the family member in this scenario? | - Positioning the family member at the side or foot of the bed can allow for comfort. It is important to ensure that the family member is within eyesight of the patient, when possible - Offer a chair whenever possible - Remain physically close to the family member but be mindful of cues around touch and need for personal space. |
| **Information Sharing** | **Empathetic verbal and non-verbal communication**  Communication consistently conveyed empathy | How would you respond when the family member said, “This is all so overwhelming!” | Heart-Head-Heart Model:   - Heart: “I hear that you are overwhelmed" - Head: “The team is working to provide Junior with the support he needs.” - Heart: “I am here to support you. Please let me know if you would like to step out at any point.” |
| **Information Sharing** | **Information quantity**  Skillfully elicited and tailored quantity of information to patient/family preferences/needs | Which elements of the resuscitation would you narrate? | - Take cues from the family member: the intubation is an appropriate procedure to narrate - Give information in small amounts and elicit whether family member wants to know more - Answer only questions asked - Allow pauses for information processing |
| **Information Sharing** | **Information content**  Information shared was consistently appropriate for the listener to understand and not laden with medical jargon | Provide two examples of how you would phrase explanations of the medical care being provided. | **Examples:**   - “The team is getting ready to place a breathing tube so that Junior can get oxygen to his body.” - “The team is concerned that he may have a serious infection and are giving him antibiotics to help his body fight the infection.” - Focus discussion of procedures on how they help the child (e.g., “The mask is helping Junior’s body get oxygen”) or on physiologic facts (e.g., “Junior’s heart is beating strongly.”) |
| **Information Sharing** | **Objective, non-speculative information**  Consistently provided objective clinical information without inappropriate speculation about the future | How would you answer the question, “Is he going to be ok?” | Heart-Head-Heart:   - “I can see that you are concerned.” - “The team is working very hard to provide Junior with the support he needs. His oxygen level and blood pressure are both increasing, so it looks like he is responding to the oxygen and fluids that the team is giving him.” - “You are doing a great job being here for your child and I am here for you during this difficult time.” |
| **Information Sharing** | **Questions & clarifications**  Consistently invited questions and offered clarifications, when appropriate | How would you engage the family member to encourage questions/provide clarifications? | - “Please let me know if anything you are hearing or seeing is confusing.” - “I am going to try to answer as many as I can and help you remember the others so you can address them with Dr. X.” |
| **Summary & Follow-Up** | **Summary communication**  Advocated for and facilitated summary communication with team leader | How would you facilitate a summary from the team leader? | - “I’m going to speak with the team leader to make sure she comes to talk to you as soon as she can.” |
| **Summary & Follow-Up** | **Next steps & follow-up**  Next steps and/or resources were skillfully communicated | How might you discuss the next steps with the family member? | - Reiterate plan discussed with medical team/team leader - Offer to assist with communication with additional family - Ensure additional staff is available for ongoing support (e.g., social work, chaplaincy) |
