# Supplementary material for: Development and Implementation of a Family Presence Facilitator Curriculum for Interprofessional Use in Pediatric Medical Resuscitations
Source: MedEdPORTAL. 2024 Oct 8;20:11445. doi: 10.15766/mep_2374-8265.11445 (PMC11458738; doi:10.15766/mep_2374-8265.11445)
Supplement: Supplementary file 1 — FPF Curriculum.pptxFPF Curriculum Recording.mp4Role-Play Script Without FPF.docxRole-Play Script With FPF.docxFPF Participant Worksheet.docxFPF Instructor Worksheet.docxFPF Survey.docxSP Training.pptxSimulated Participant Training Case.docxFPF-SAT.docx [file mep_2374-8265.11445-s001.zip › G. FPF Survey.docx]

Thank you for participating in the Family Presence Facilitator (FPF) Curriculum. Please take a few minutes to complete this anonymous post-training survey. Your feedback, especially qualitative comments, is appreciated!

I enjoyed this curriculum

- Strongly disagree
- Somewhat disagree
- Somewhat agree
- Strongly agree

I found this curriculum engaging

- Strongly disagree
- Somewhat disagree
- Somewhat agree
- Strongly agree

The information in this curriculum is relevant to my practice

- Strongly disagree
- Somewhat disagree
- Somewhat agree
- Strongly agree

This curriculum increased my knowledge and skills in providing the following:

Empathetic, respectful communication to patients and families

- Strongly disagree
- Somewhat disagree
- Somewhat agree
- Strongly agree

Information that is non-speculative and easy to understand

- Strongly disagree
- Somewhat disagree
- Somewhat agree
- Strongly agree

Non-verbal support (in the form of physical touch, receptive listening skills, etc.)

- Strongly disagree
- Somewhat disagree
- Somewhat agree
- Strongly agree

This curriculum increased my confidence in providing patient- and family-centered communication as an FPF in the Pediatric Emergency Department during medical resuscitations

- Strongly disagree
- Somewhat disagree
- Somewhat agree
- Strongly agree

Implementation of this curriculum will improve the patient care experience

- Strongly disagree
- Somewhat disagree
- Somewhat agree
- Strongly agree

Based on this curriculum, I am likely to change my practice regarding family presence during resuscitations

- Strongly disagree
- Somewhat disagree
- Somewhat agree
- Strongly agree

If you answered somewhat agree or strongly agree to the previous question, please describe how you may change your practice

Did you participate in hands-on practice during the workshop?

If yes, please answer the following questions about the hands-on practice component of the curriculum:

I found the hands-on component to be useful

- Strongly disagree
- Somewhat disagree
- Somewhat agree
- Strongly agree

**Demographic information**

Have you received similar training prior to the session?

- Yes
- No

What is your gender?

- Male
- Female
- Non-binary / third gender
- Prefer not to say

What is your age?

- 25 years or younger
- 26-35 years
- 36-45 years
- 46-55 years
- 56 years or older

What is your occupation?

- Attending physician
- Fellow
- Resident
- Medical student
- Nurse
- Advanced practice provider
- Child life specialist
- Social worker
- Chaplain
- Other (free text)

Any additional comments or suggestions?
