# Supplementary material for: Development and Implementation of a Family Presence Facilitator Curriculum for Interprofessional Use in Pediatric Medical Resuscitations
Source: MedEdPORTAL. 2024 Oct 8;20:11445. doi: 10.15766/mep_2374-8265.11445 (PMC11458738; doi:10.15766/mep_2374-8265.11445)

## Slide 1
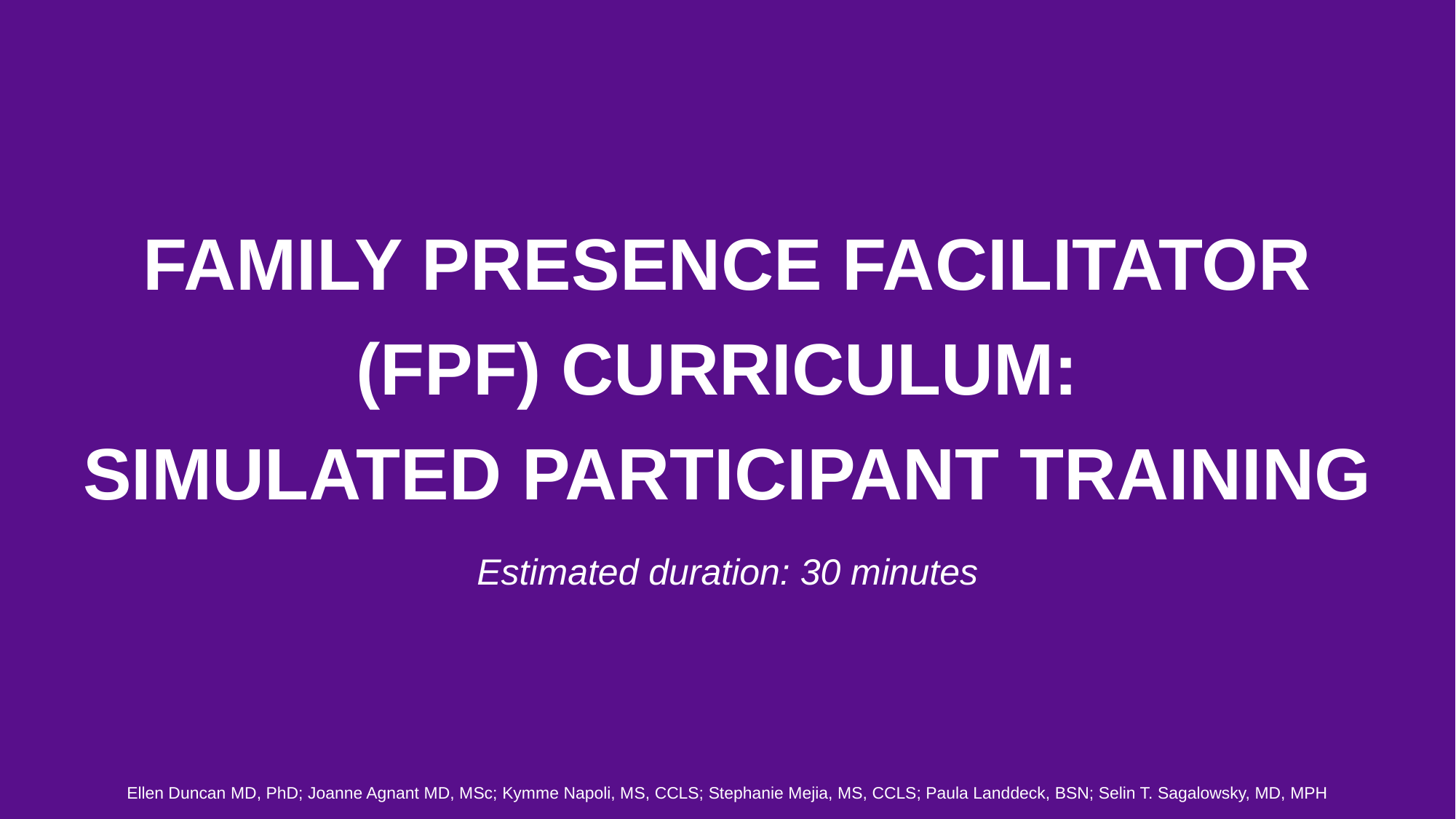

# Family Presence Facilitator (FPF) Curriculum: simulated PARTICIPANT Training
Estimated duration: 30 minutes
Ellen Duncan MD, PhD; Joanne Agnant MD, MSc; Kymme Napoli, MS, CCLS; Stephanie Mejia, MS, CCLS; Paula Landdeck, BSN; Selin T. Sagalowsky, MD, MPH

## Slide 2
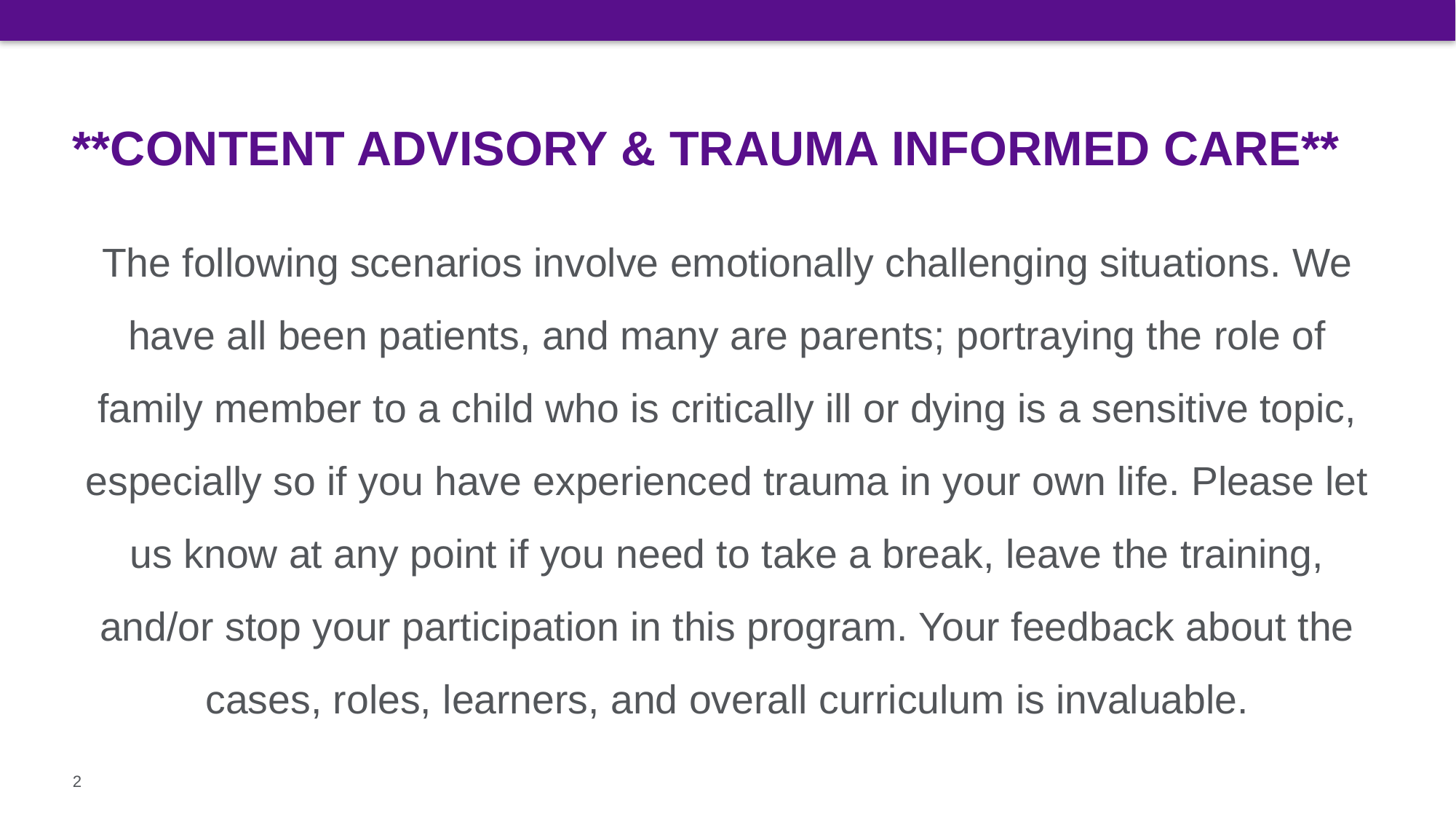

# **CONTENT ADVISORY & TRAUMA INFORMED CARE**
The following scenarios involve emotionally challenging situations. We have all been patients, and many are parents; portraying the role of family member to a child who is critically ill or dying is a sensitive topic, especially so if you have experienced trauma in your own life. Please let us know at any point if you need to take a break, leave the training, and/or stop your participation in this program. Your feedback about the cases, roles, learners, and overall curriculum is invaluable.
2

## Slide 3
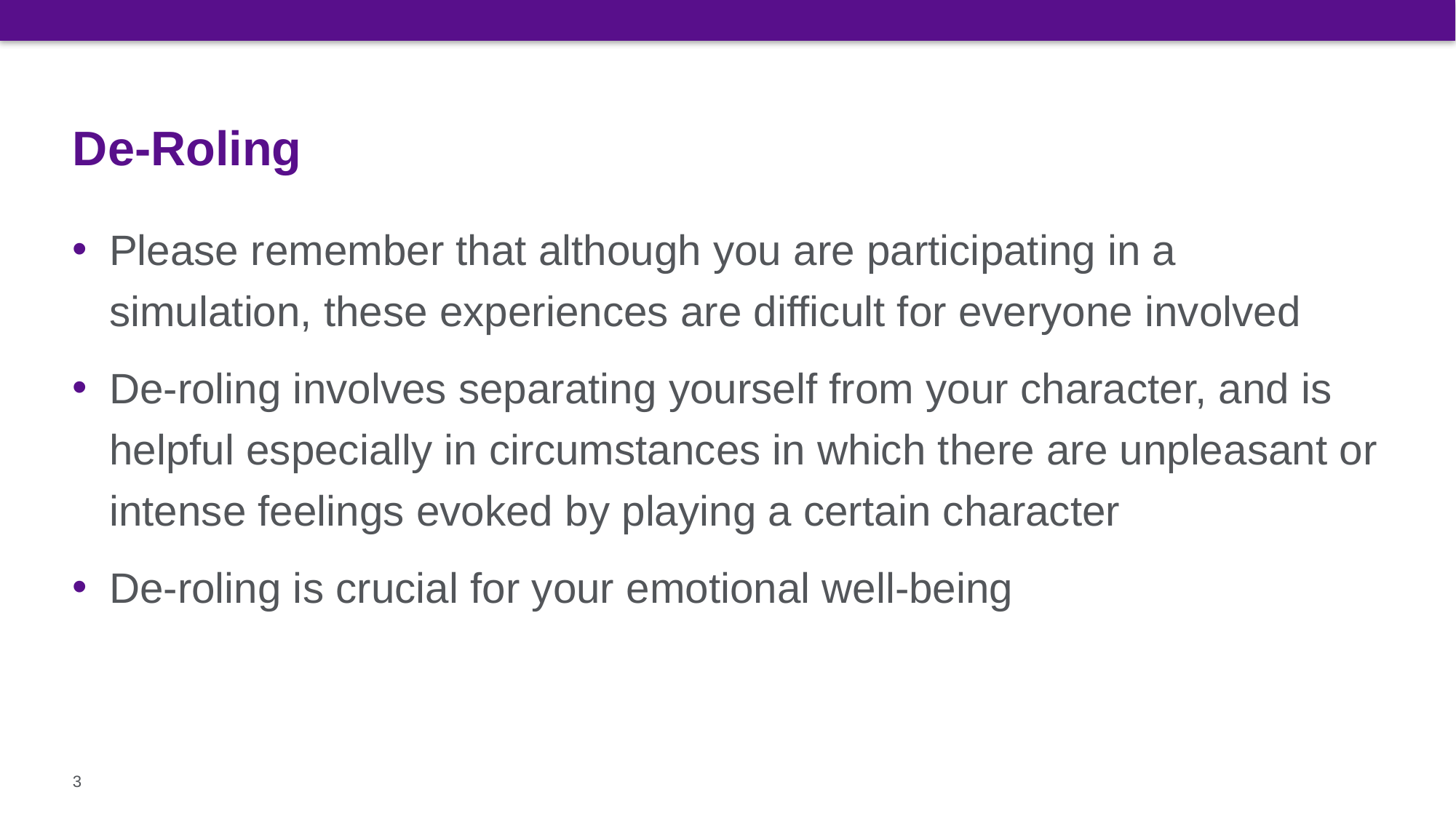

# De-Roling
Please remember that although you are participating in a simulation, these experiences are difficult for everyone involved
De-roling involves separating yourself from your character, and is helpful especially in circumstances in which there are unpleasant or intense feelings evoked by playing a certain character
De-roling is crucial for your emotional well-being
3

## Slide 4
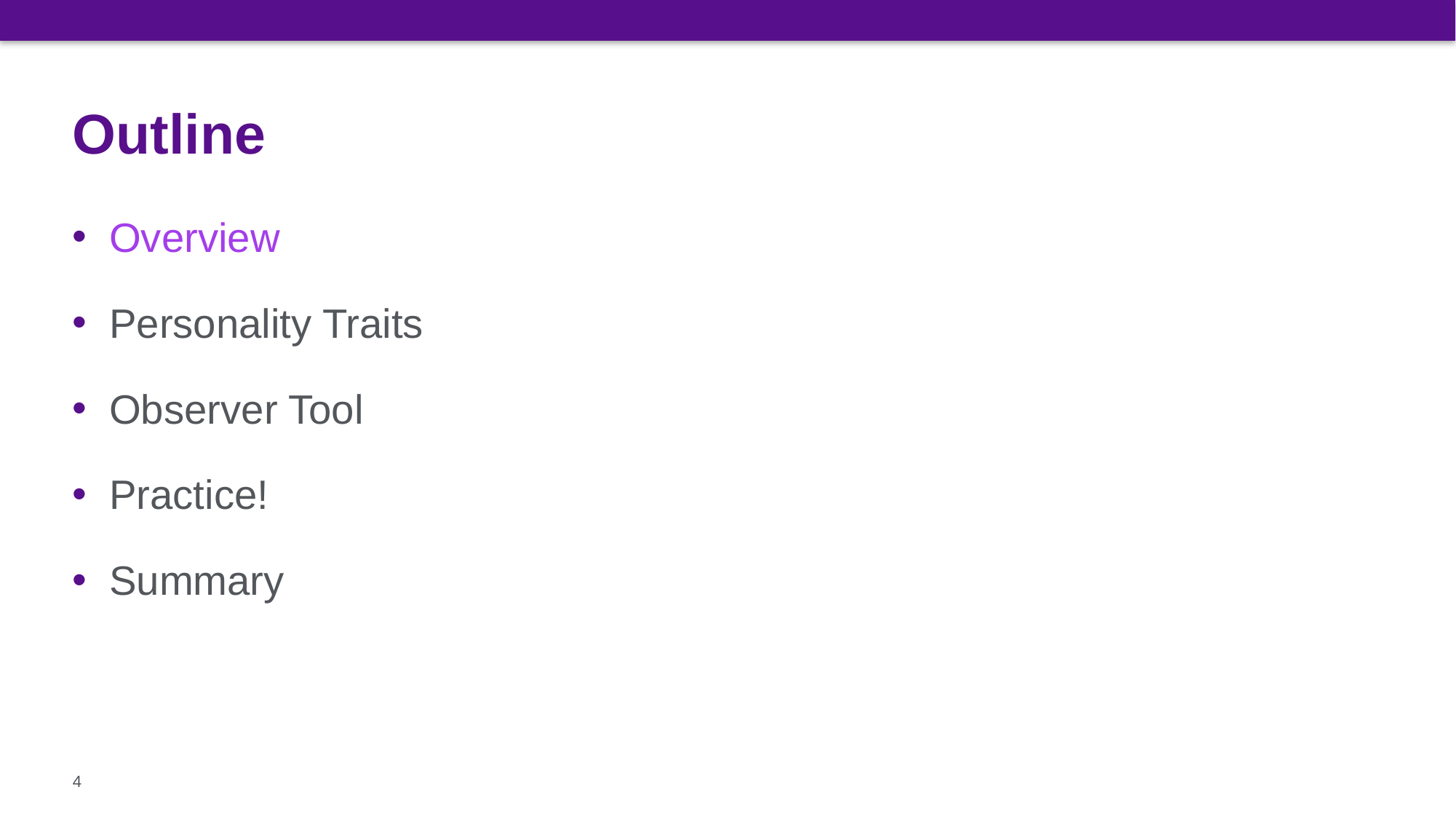

# Outline
Overview
Personality Traits
Observer Tool
Practice!
Summary
4

## Slide 5
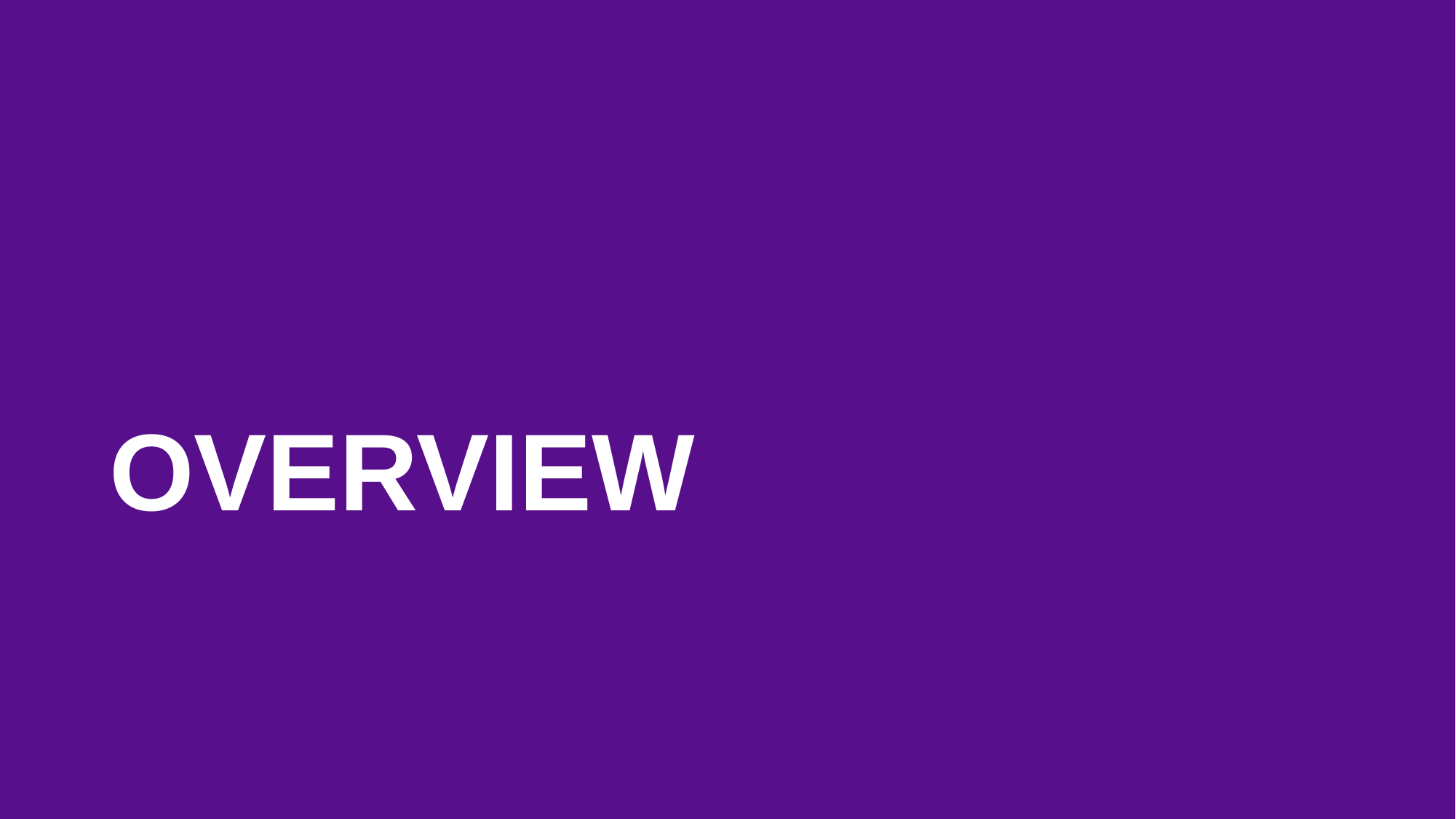

# overview

## Slide 6
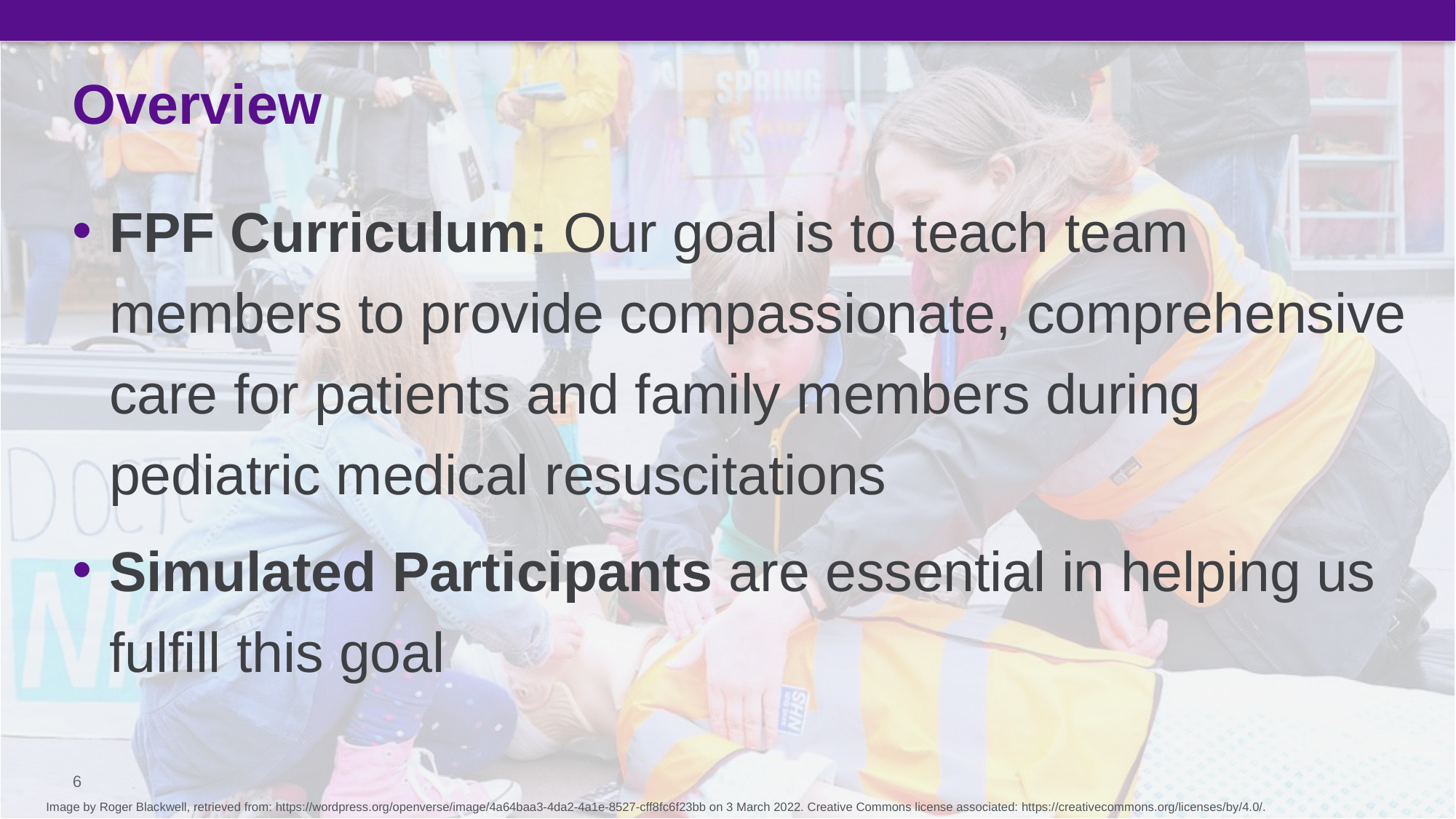

# Overview
FPF Curriculum: Our goal is to teach team members to provide compassionate, comprehensive care for patients and family members during pediatric medical resuscitations
Simulated Participants are essential in helping us fulfill this goal
6
Image by Roger Blackwell, retrieved from: https://wordpress.org/openverse/image/4a64baa3-4da2-4a1e-8527-cff8fc6f23bb on 3 March 2022. Creative Commons license associated: https://creativecommons.org/licenses/by/4.0/.

## Slide 7
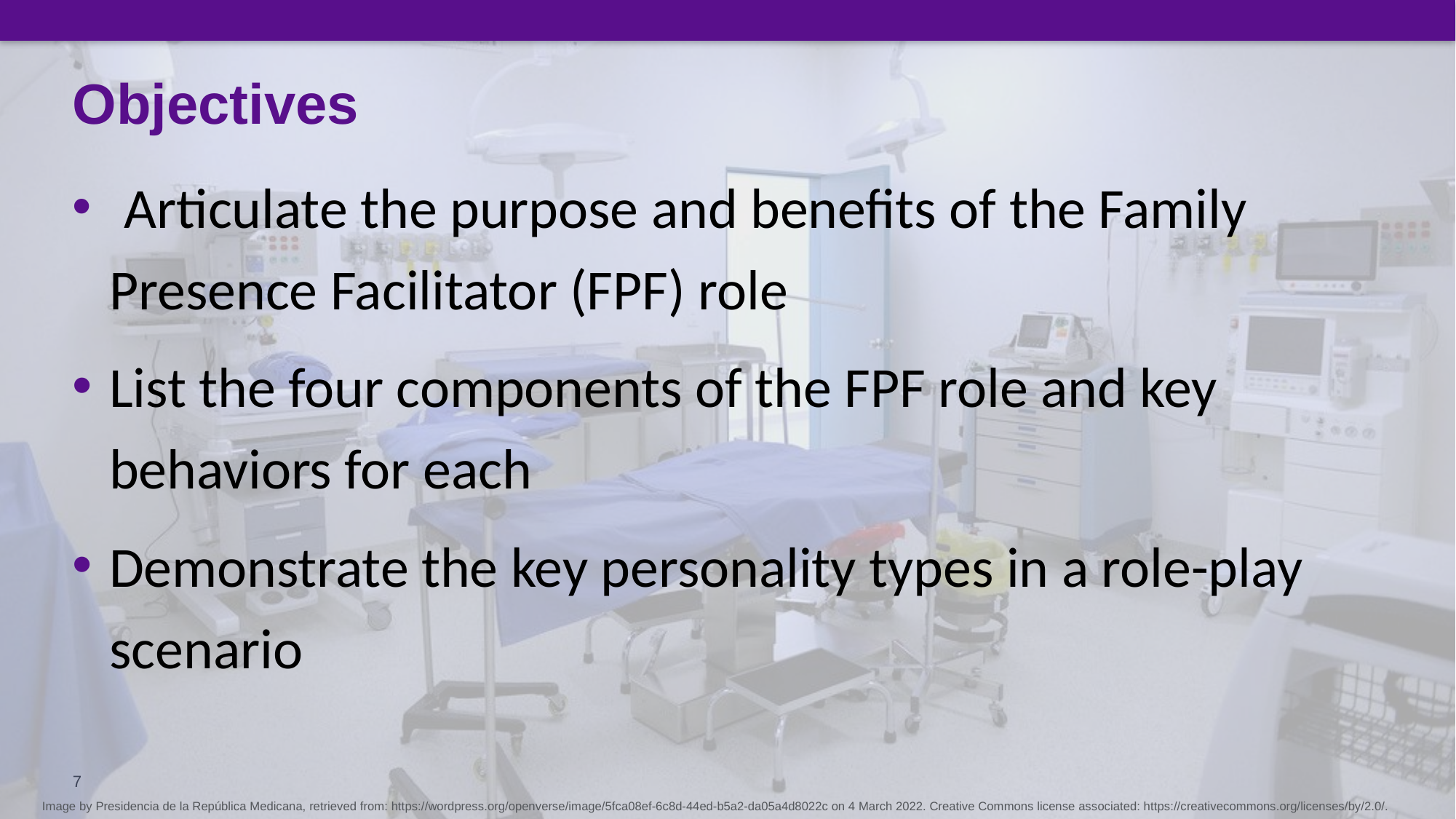

# Objectives
 Articulate the purpose and benefits of the Family Presence Facilitator (FPF) role
List the four components of the FPF role and key behaviors for each
Demonstrate the key personality types in a role-play scenario
7
Image by Presidencia de la República Medicana, retrieved from: https://wordpress.org/openverse/image/5fca08ef-6c8d-44ed-b5a2-da05a4d8022c on 4 March 2022. Creative Commons license associated: https://creativecommons.org/licenses/by/2.0/.

## Slide 8
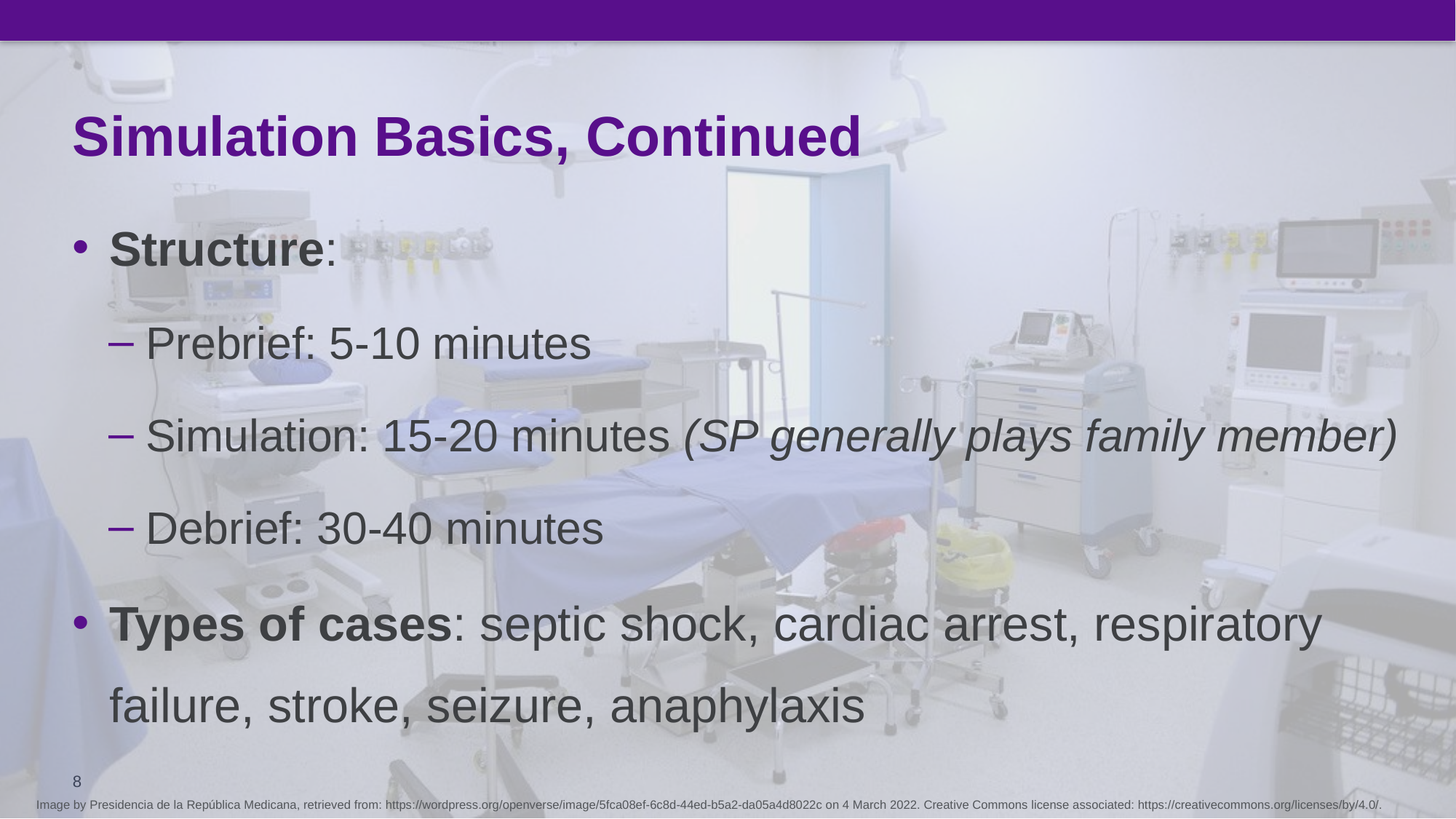

# Simulation Basics, Continued
Structure:
Prebrief: 5-10 minutes
Simulation: 15-20 minutes (SP generally plays family member)
Debrief: 30-40 minutes
Types of cases: septic shock, cardiac arrest, respiratory failure, stroke, seizure, anaphylaxis
8
Image by Presidencia de la República Medicana, retrieved from: https://wordpress.org/openverse/image/5fca08ef-6c8d-44ed-b5a2-da05a4d8022c on 4 March 2022. Creative Commons license associated: https://creativecommons.org/licenses/by/4.0/.

## Slide 9
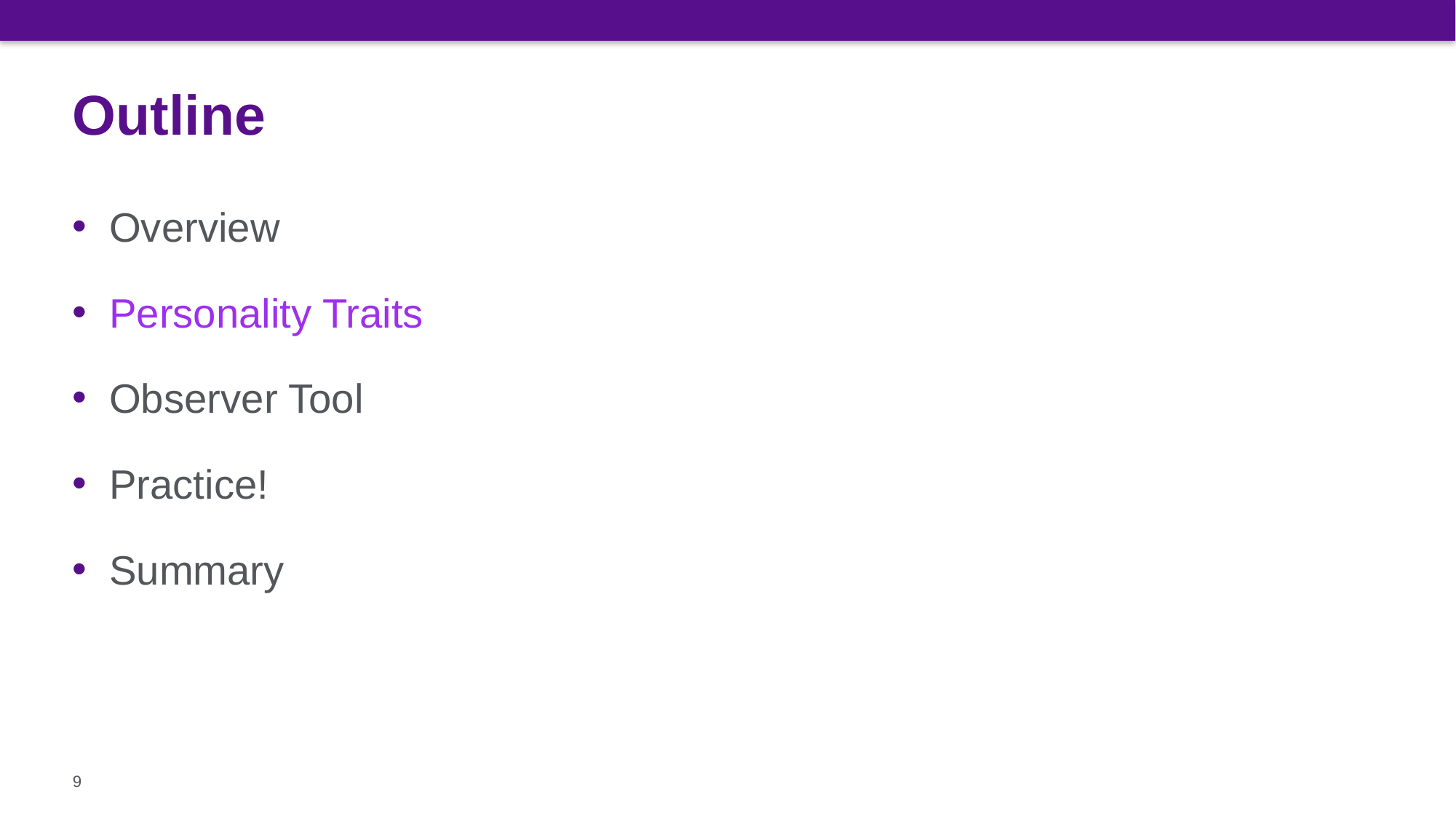

# Outline
Overview
Personality Traits
Observer Tool
Practice!
Summary
9

## Slide 10
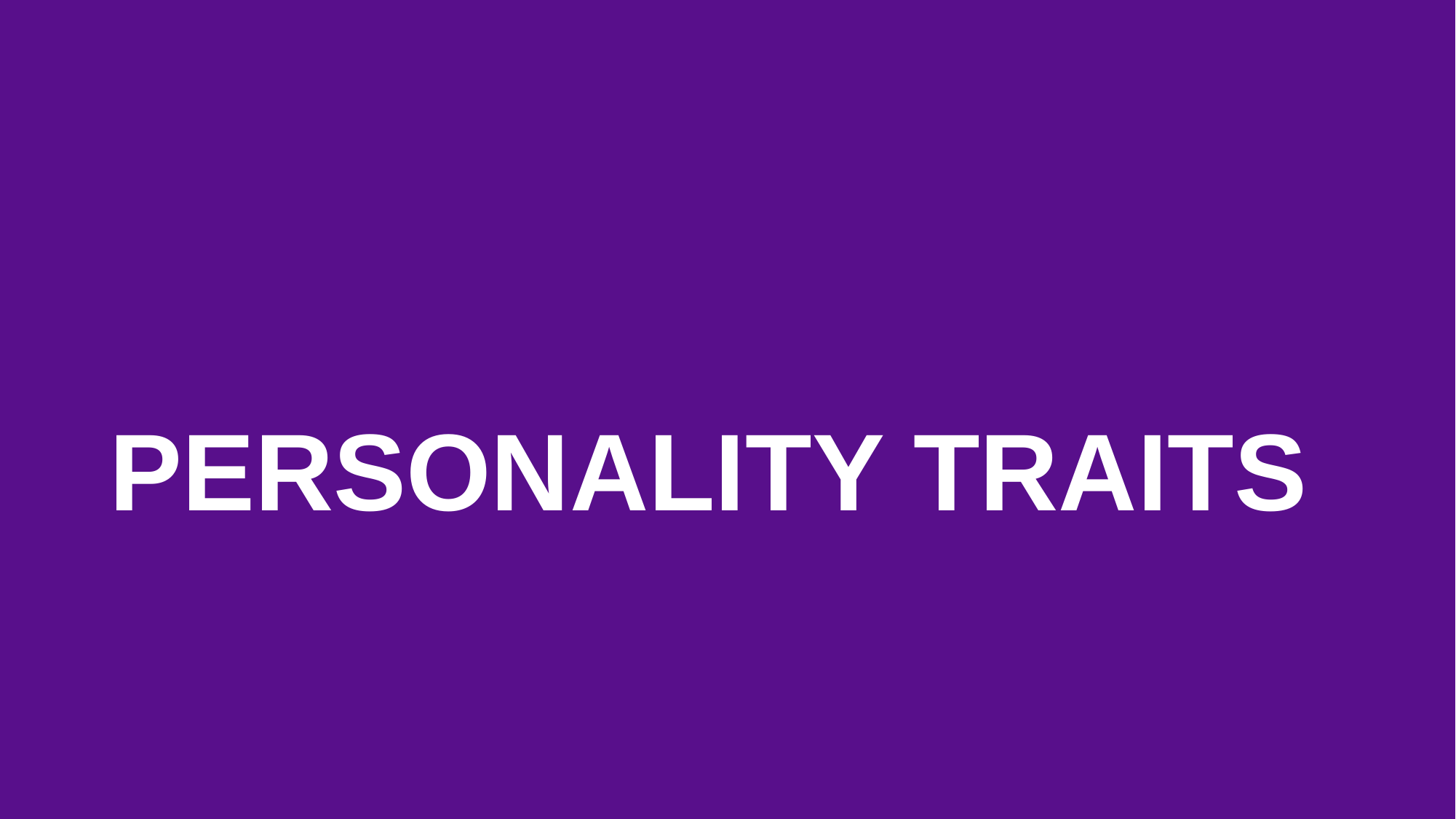

# Personality traits

## Slide 11
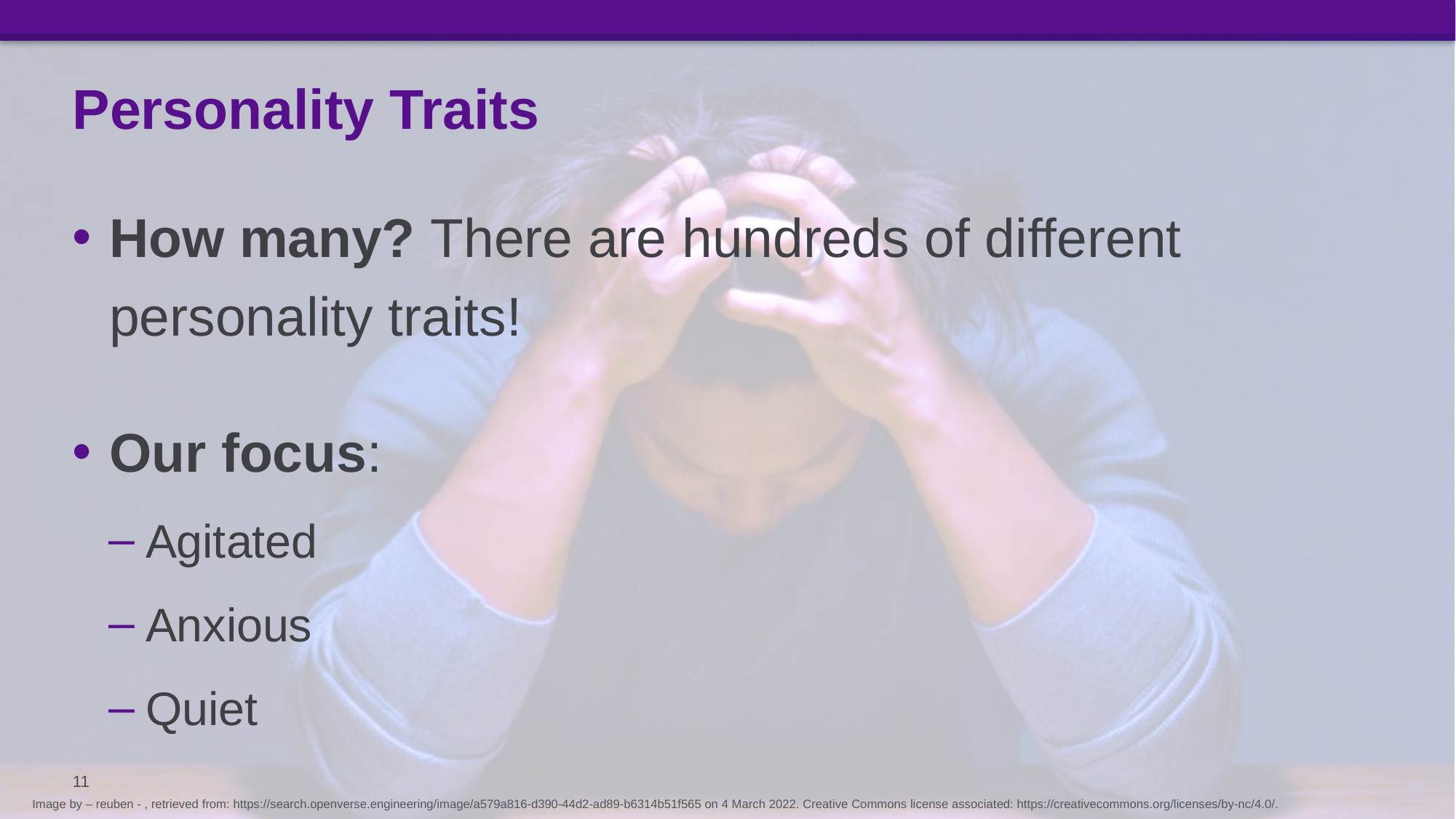

# Personality Traits
How many? There are hundreds of different personality traits!
Our focus:
Agitated
Anxious
Quiet
11
Image by – reuben - , retrieved from: https://search.openverse.engineering/image/a579a816-d390-44d2-ad89-b6314b51f565 on 4 March 2022. Creative Commons license associated: https://creativecommons.org/licenses/by-nc/4.0/.

## Slide 12
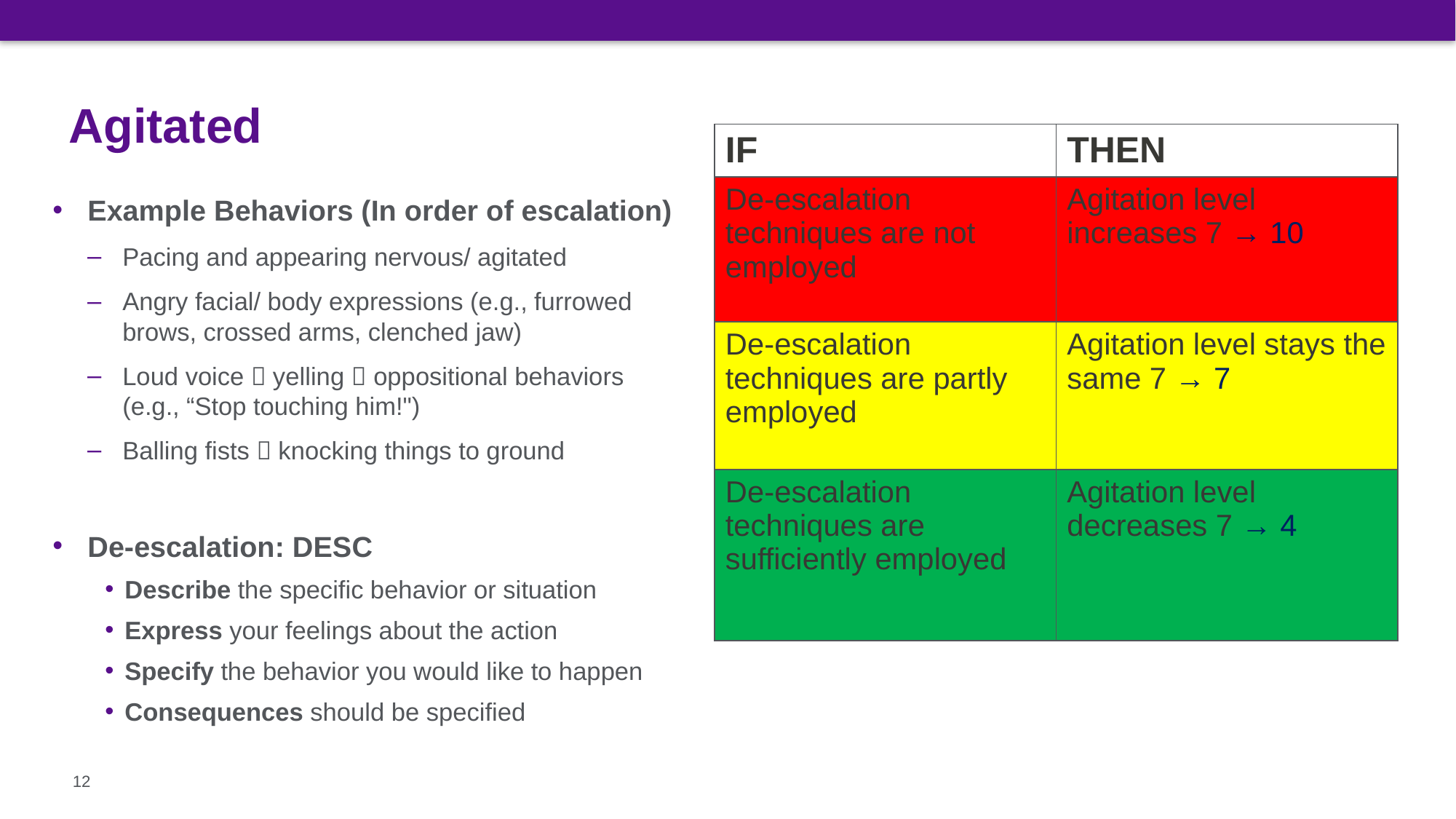

# Agitated
| IF | THEN |
| --- | --- |
| De-escalation techniques are not employed | Agitation level increases 7 → 10 |
| De-escalation techniques are partly employed | Agitation level stays the same 7 → 7 |
| De-escalation techniques are sufficiently employed | Agitation level decreases 7 → 4 |
Example Behaviors (In order of escalation)
Pacing and appearing nervous/ agitated
Angry facial/ body expressions (e.g., furrowed brows, crossed arms, clenched jaw)
Loud voice  yelling  oppositional behaviors (e.g., “Stop touching him!")
Balling fists  knocking things to ground
De-escalation: DESC
Describe the specific behavior or situation
Express your feelings about the action
Specify the behavior you would like to happen
Consequences should be specified
12

## Slide 13
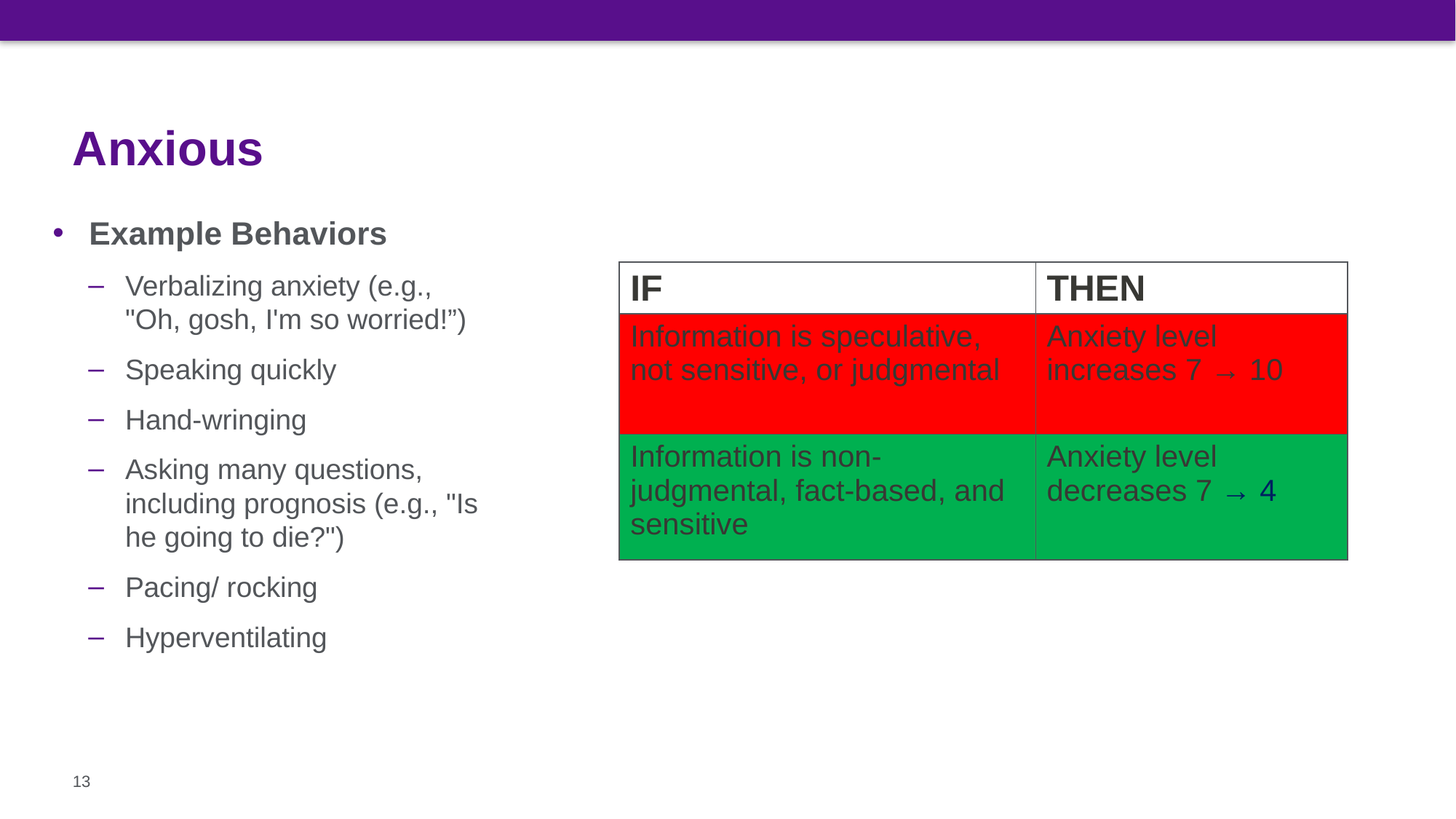

# Anxious
Example Behaviors
Verbalizing anxiety (e.g., "Oh, gosh, I'm so worried!”)
Speaking quickly
Hand-wringing
Asking many questions, including prognosis (e.g., "Is he going to die?")
Pacing/ rocking
Hyperventilating
| IF | THEN |
| --- | --- |
| Information is speculative, not sensitive, or judgmental | Anxiety level increases 7 → 10 |
| Information is non-judgmental, fact-based, and sensitive | Anxiety level decreases 7 → 4 |
13

## Slide 14
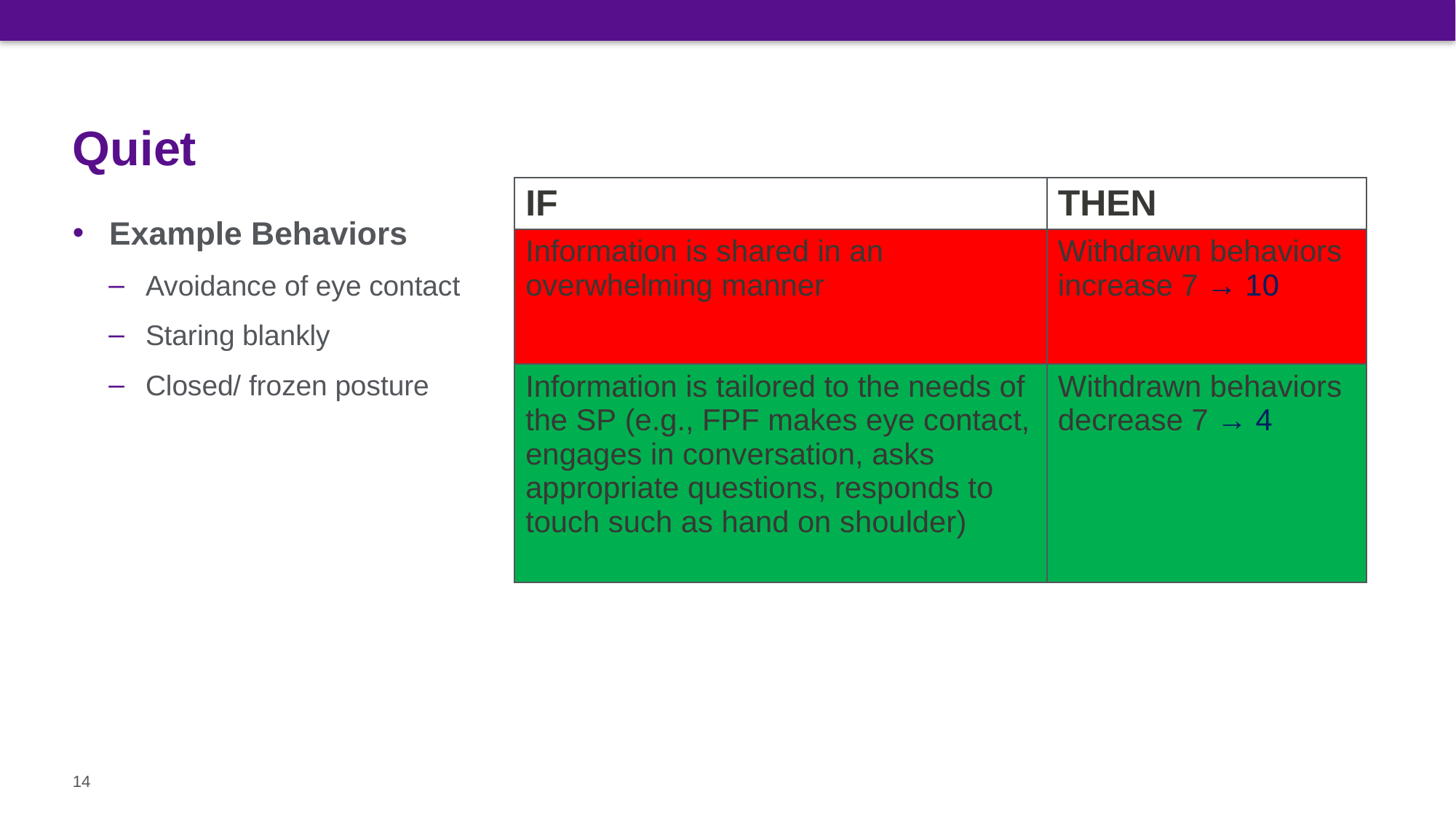

# Quiet
| IF | THEN |
| --- | --- |
| Information is shared in an overwhelming manner | Withdrawn behaviors increase 7 → 10 |
| Information is tailored to the needs of the SP (e.g., FPF makes eye contact, engages in conversation, asks appropriate questions, responds to touch such as hand on shoulder) | Withdrawn behaviors decrease 7 → 4 |
Example Behaviors
Avoidance of eye contact
Staring blankly
Closed/ frozen posture
14

## Slide 15
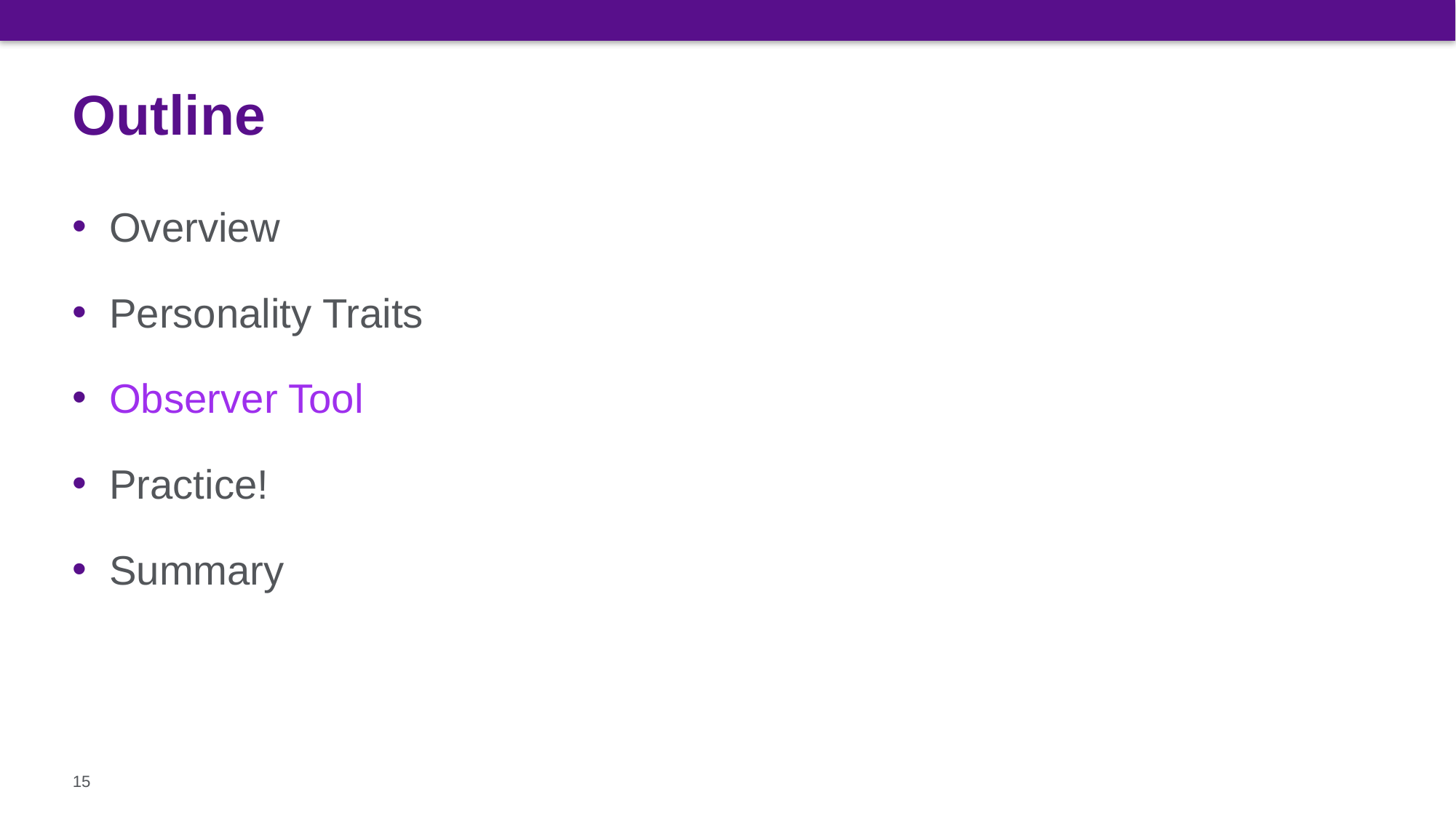

# Outline
Overview
Personality Traits
Observer Tool
Practice!
Summary
15

## Slide 16
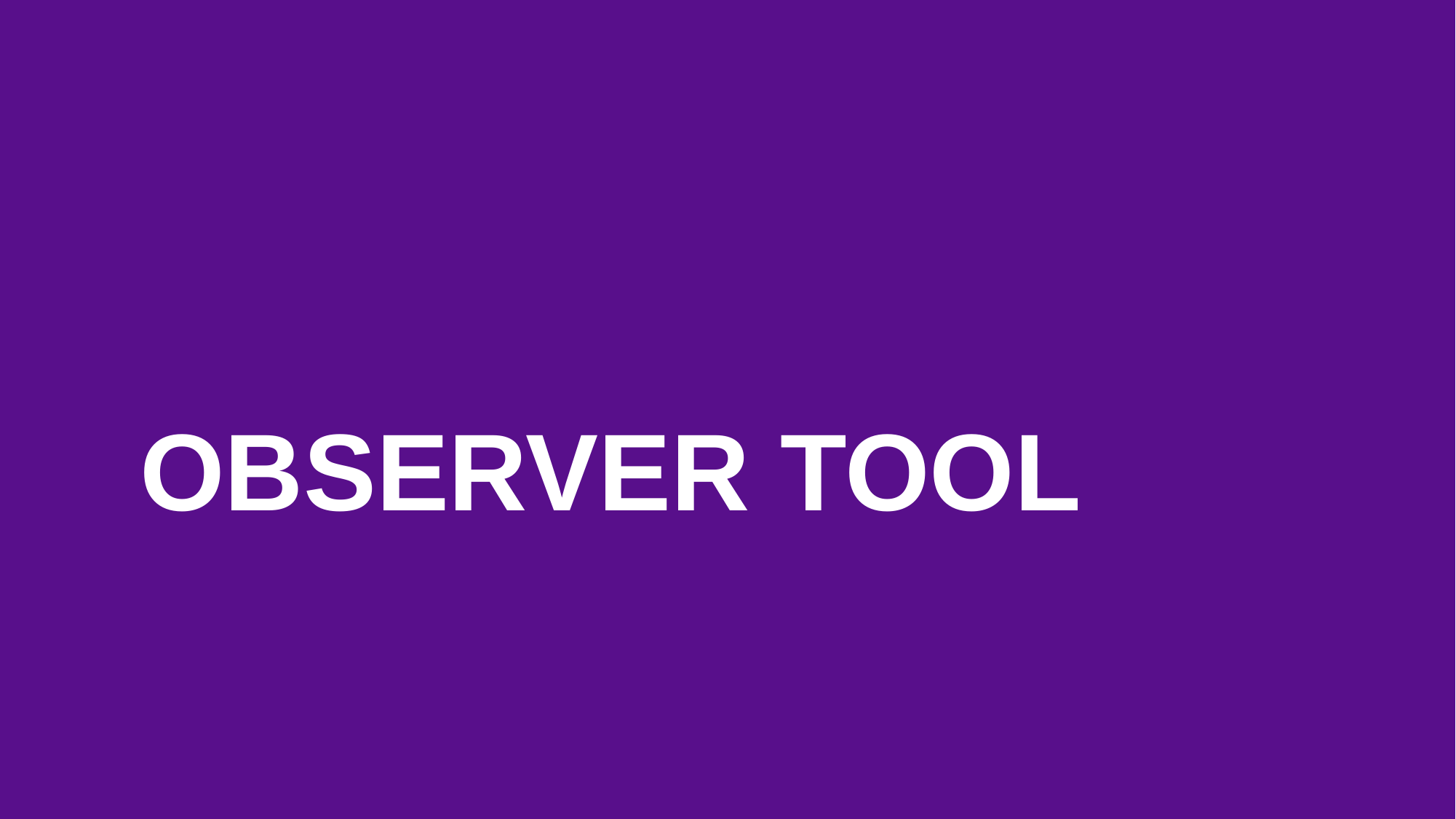

# OBSERVER TOOL

## Slide 17
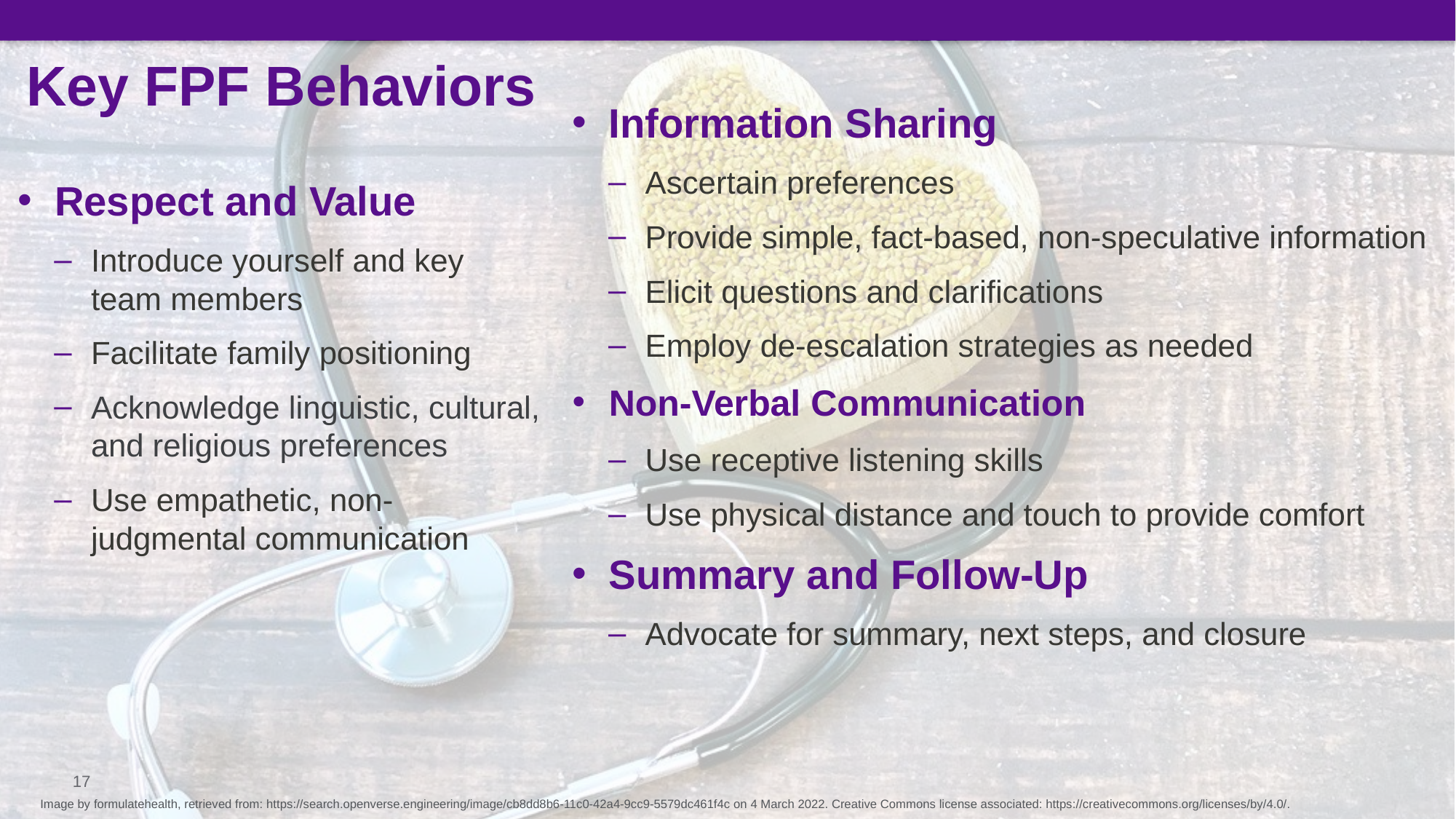

# Key FPF Behaviors
Information Sharing
Ascertain preferences
Provide simple, fact-based, non-speculative information
Elicit questions and clarifications
Employ de-escalation strategies as needed
Non-Verbal Communication
Use receptive listening skills
Use physical distance and touch to provide comfort
Summary and Follow-Up
Advocate for summary, next steps, and closure
Respect and Value
Introduce yourself and key team members
Facilitate family positioning
Acknowledge linguistic, cultural, and religious preferences
Use empathetic, non-judgmental communication
17
Image by formulatehealth, retrieved from: https://search.openverse.engineering/image/cb8dd8b6-11c0-42a4-9cc9-5579dc461f4c on 4 March 2022. Creative Commons license associated: https://creativecommons.org/licenses/by/4.0/.

## Slide 18
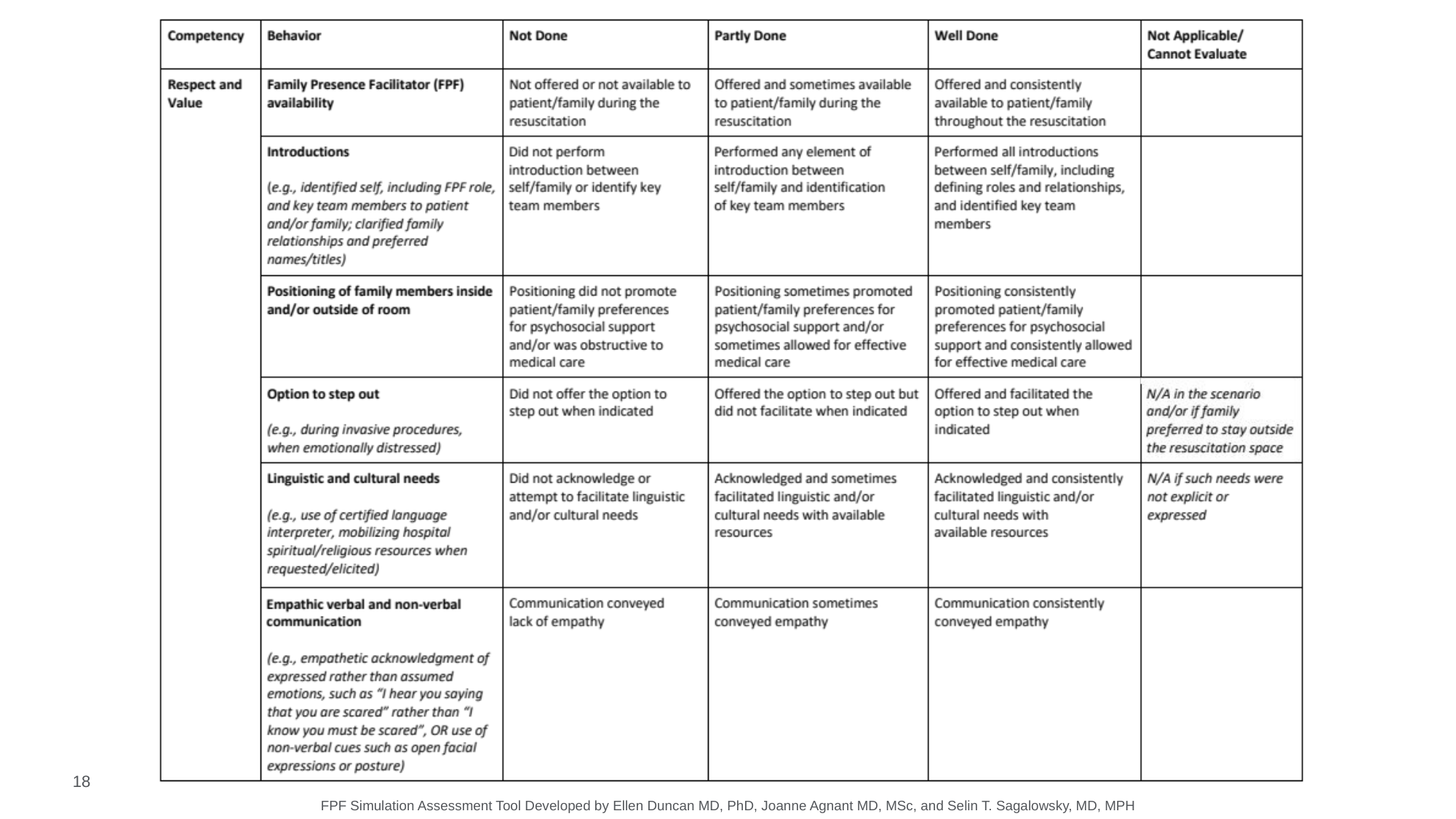

18
FPF Simulation Assessment Tool Developed by Ellen Duncan MD, PhD, Joanne Agnant MD, MSc, and Selin T. Sagalowsky, MD, MPH

## Slide 19
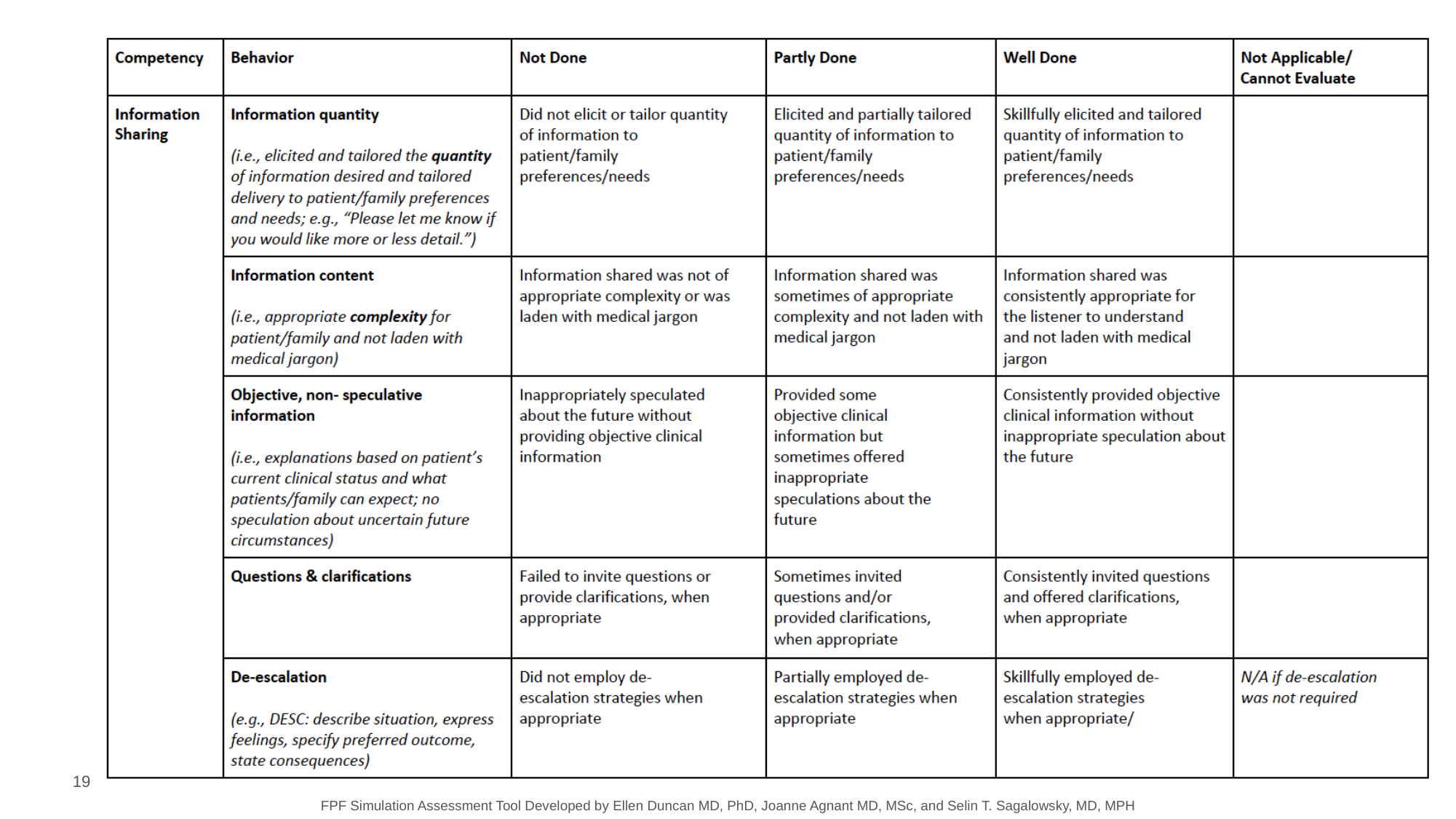

19
FPF Simulation Assessment Tool Developed by Ellen Duncan MD, PhD, Joanne Agnant MD, MSc, and Selin T. Sagalowsky, MD, MPH

## Slide 20
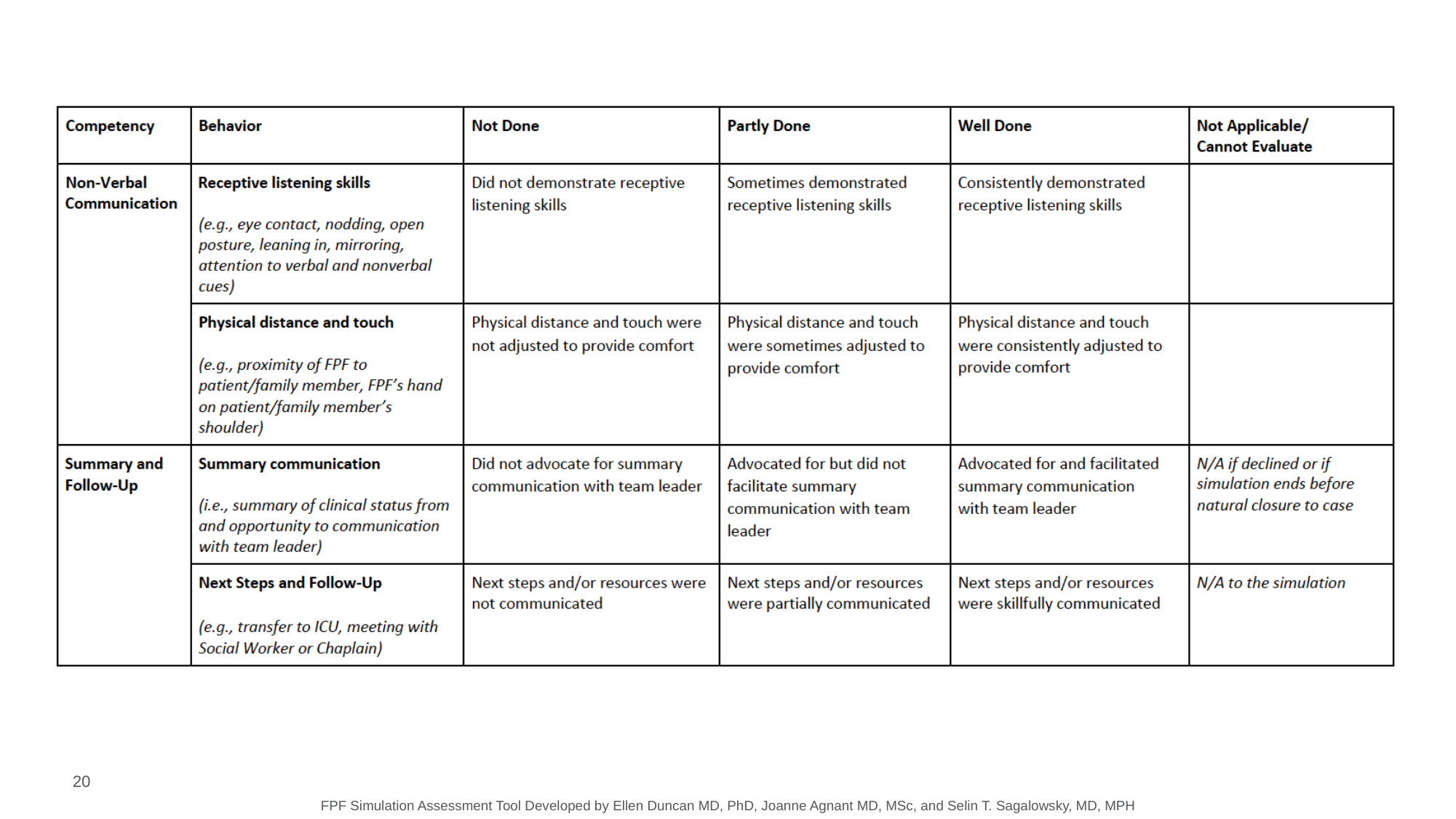

20
FPF Simulation Assessment Tool Developed by Ellen Duncan MD, PhD, Joanne Agnant MD, MSc, and Selin T. Sagalowsky, MD, MPH

## Slide 21
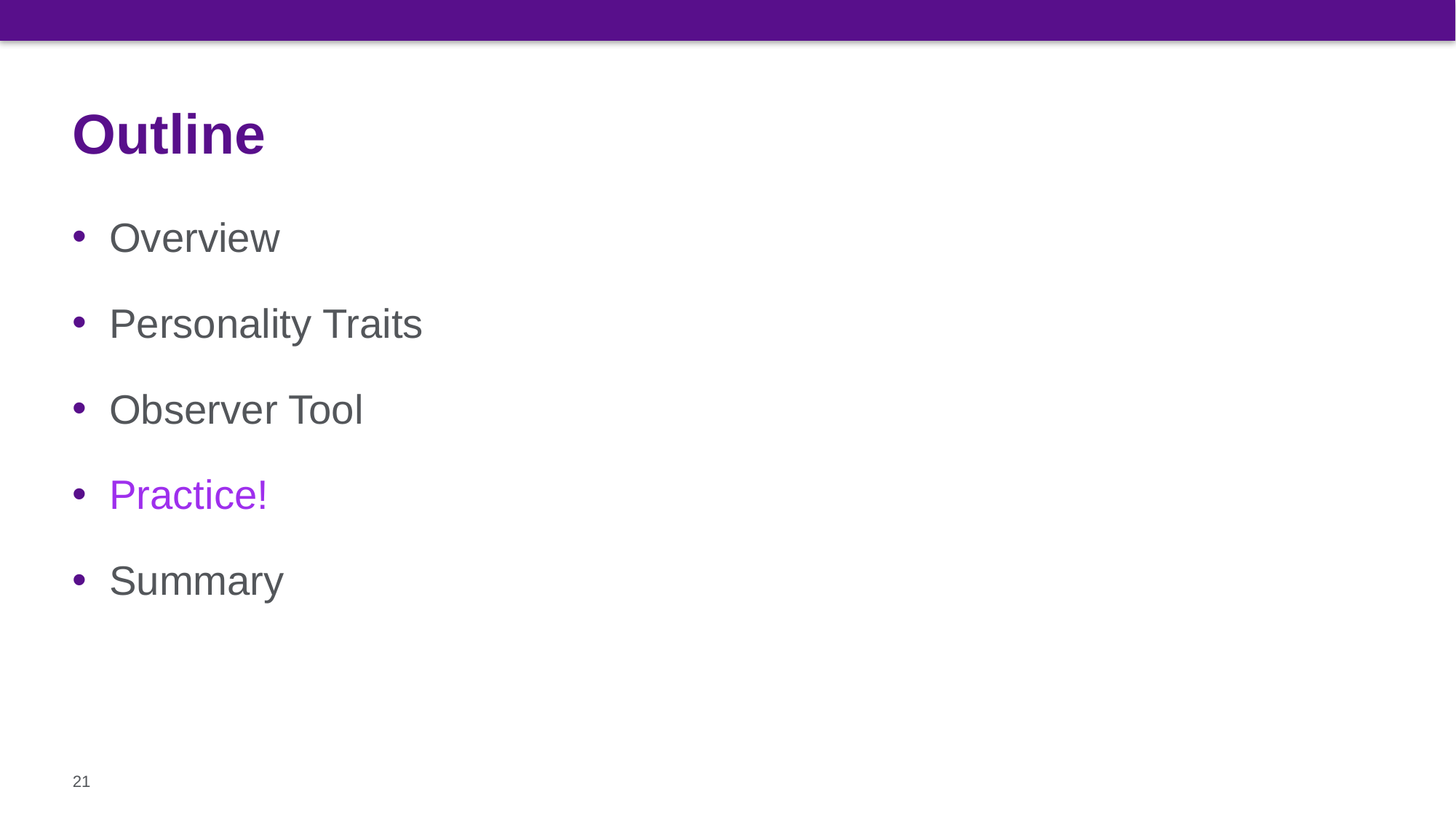

# Outline
Overview
Personality Traits
Observer Tool
Practice!
Summary
21

## Slide 22
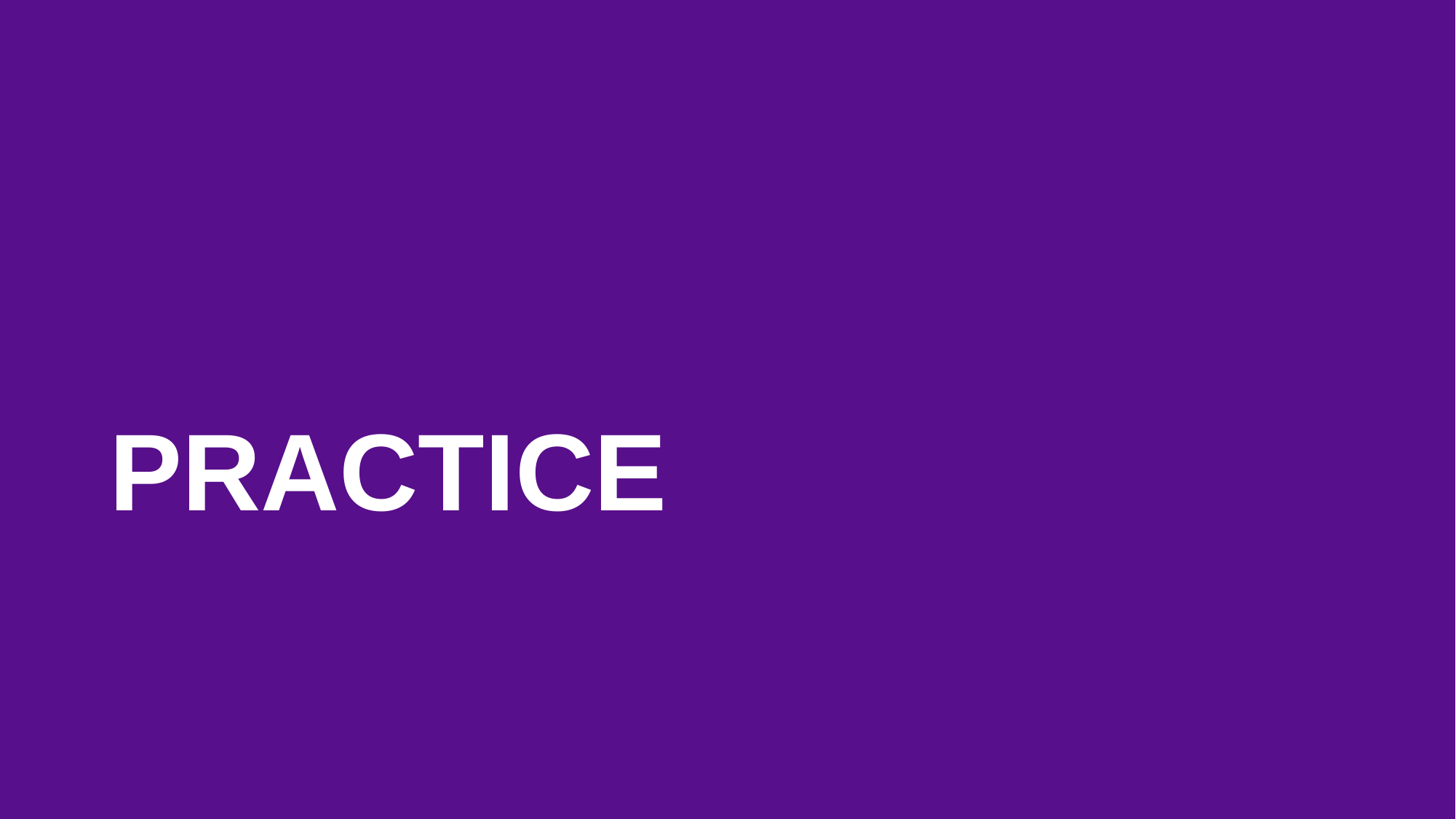

# Practice

## Slide 23
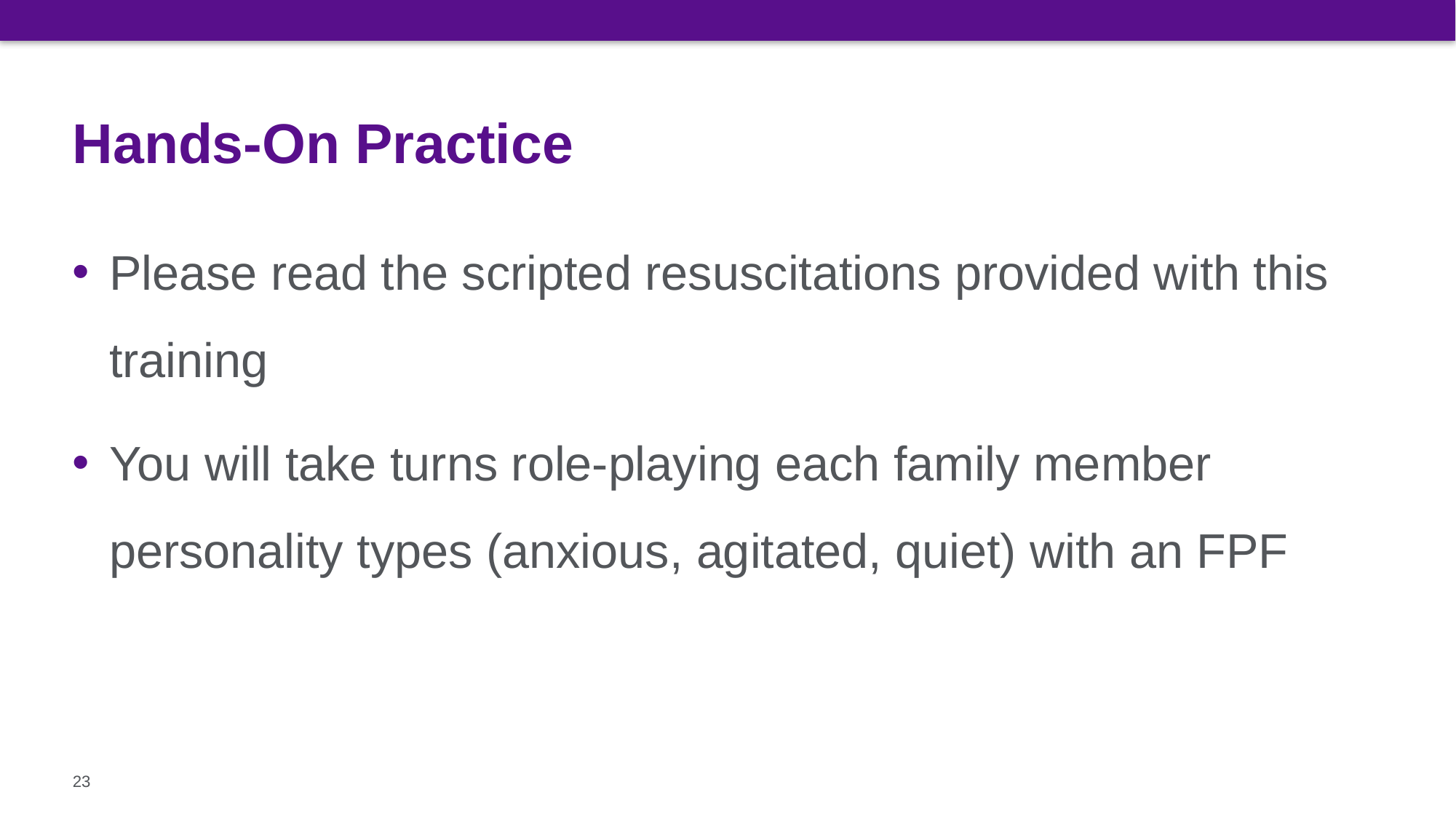

# Hands-On Practice
Please read the scripted resuscitations provided with this training
You will take turns role-playing each family member personality types (anxious, agitated, quiet) with an FPF
23

## Slide 24
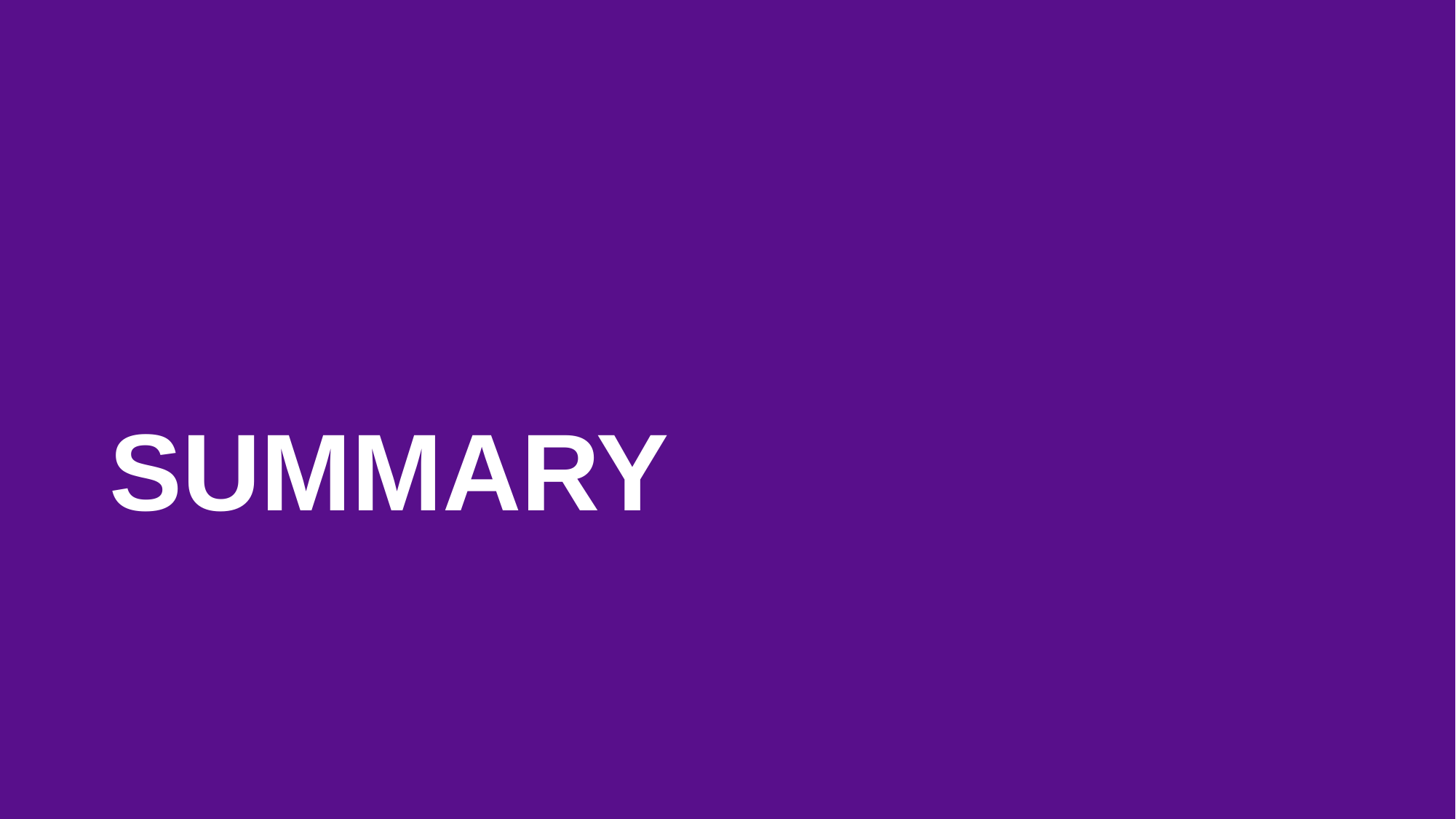

# Summary

## Slide 25
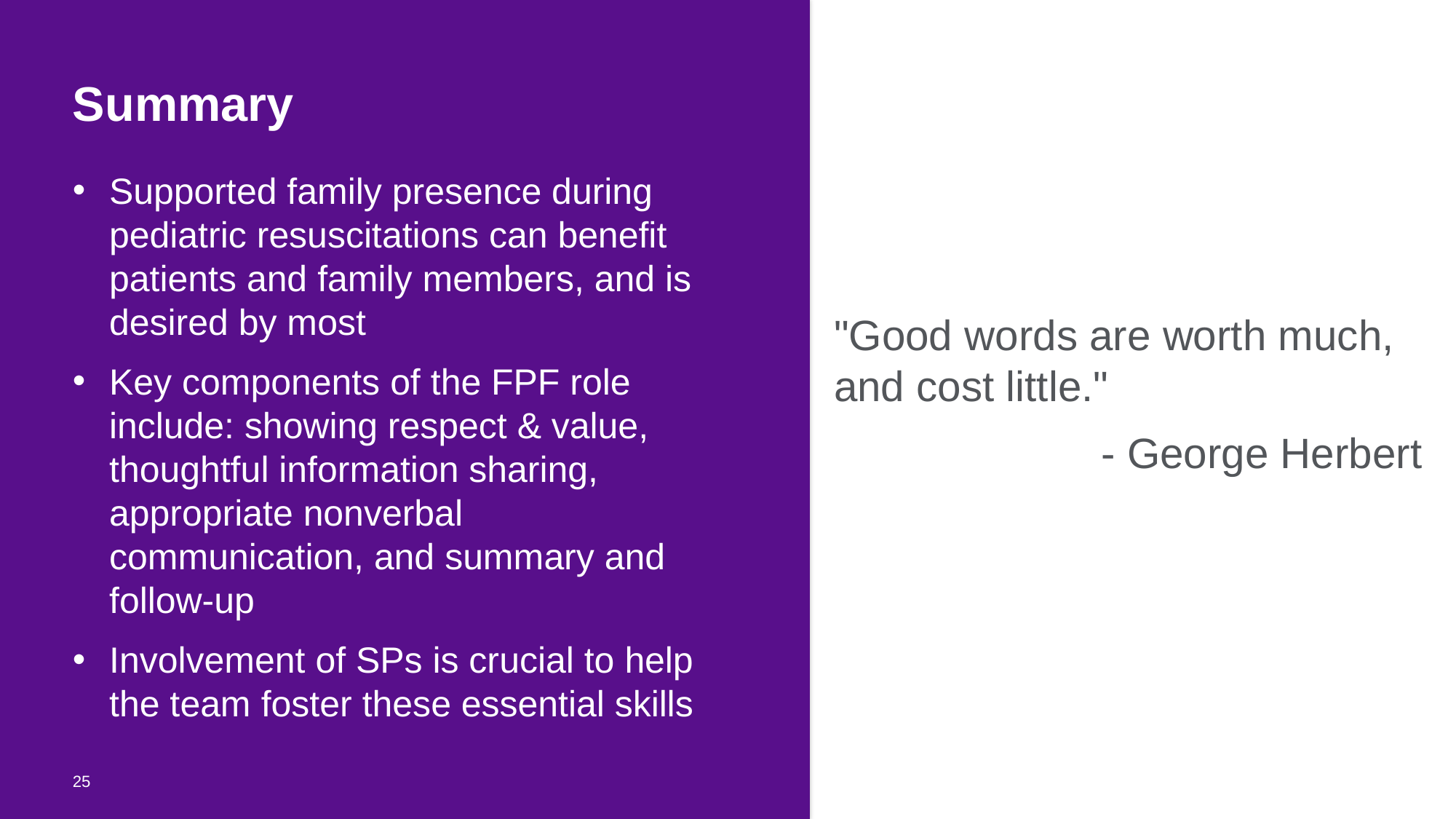

# Summary
Supported family presence during pediatric resuscitations can benefit patients and family members, and is desired by most
Key components of the FPF role include: showing respect & value, thoughtful information sharing, appropriate nonverbal communication, and summary and follow-up
Involvement of SPs is crucial to help the team foster these essential skills
"Good words are worth much, and cost little."
- George Herbert
25

## Slide 26
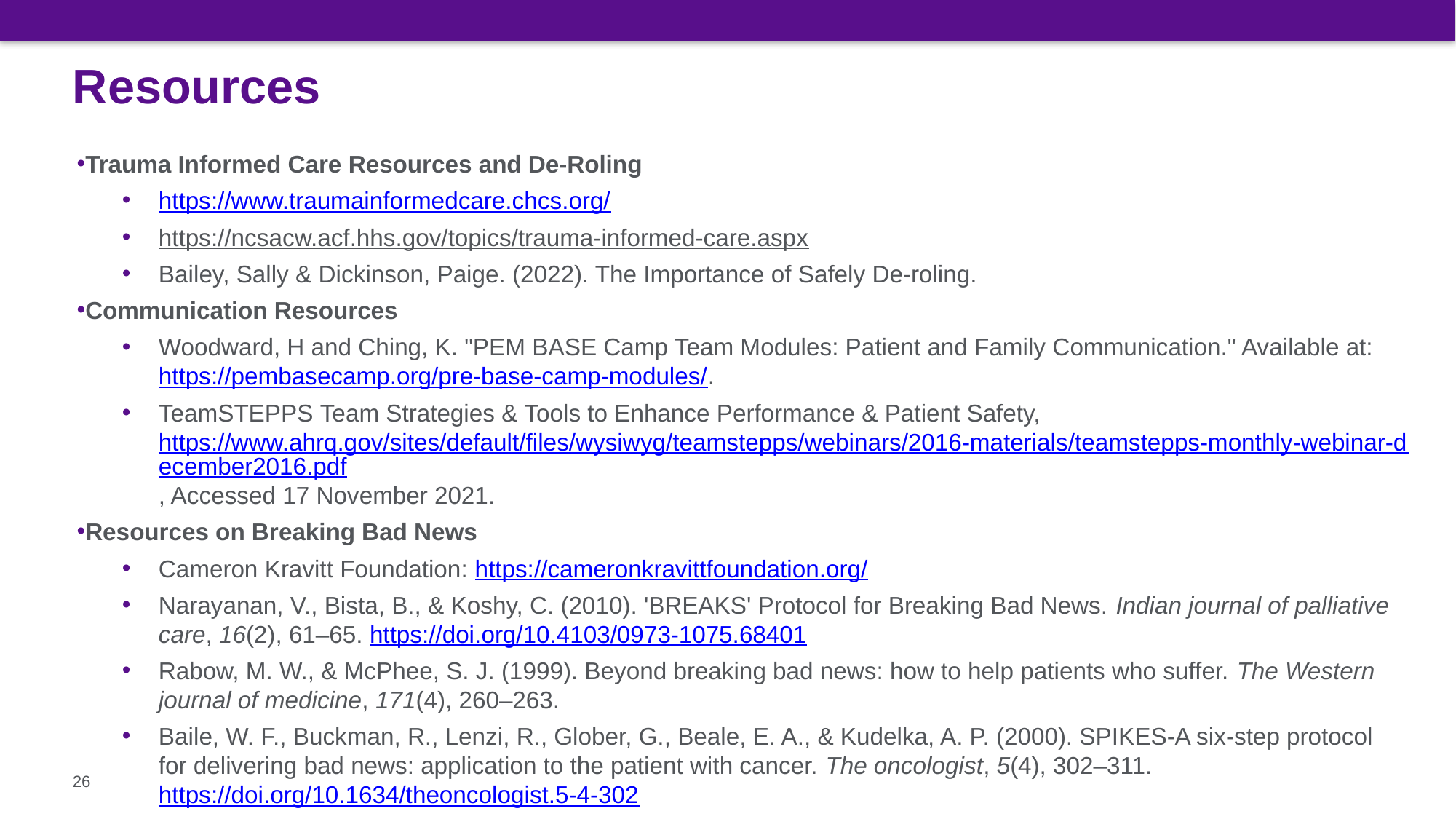

# Resources
Trauma Informed Care Resources and De-Roling
https://www.traumainformedcare.chcs.org/
https://ncsacw.acf.hhs.gov/topics/trauma-informed-care.aspx
Bailey, Sally & Dickinson, Paige. (2022). The Importance of Safely De-roling.
Communication Resources
Woodward, H and Ching, K. "PEM BASE Camp Team Modules: Patient and Family Communication." Available at: https://pembasecamp.org/pre-base-camp-modules/.
TeamSTEPPS Team Strategies & Tools to Enhance Performance & Patient Safety, https://www.ahrq.gov/sites/default/files/wysiwyg/teamstepps/webinars/2016-materials/teamstepps-monthly-webinar-december2016.pdf, Accessed 17 November 2021.
Resources on Breaking Bad News
Cameron Kravitt Foundation: https://cameronkravittfoundation.org/
Narayanan, V., Bista, B., & Koshy, C. (2010). 'BREAKS' Protocol for Breaking Bad News. Indian journal of palliative care, 16(2), 61–65. https://doi.org/10.4103/0973-1075.68401
Rabow, M. W., & McPhee, S. J. (1999). Beyond breaking bad news: how to help patients who suffer. The Western journal of medicine, 171(4), 260–263.
Baile, W. F., Buckman, R., Lenzi, R., Glober, G., Beale, E. A., & Kudelka, A. P. (2000). SPIKES-A six-step protocol for delivering bad news: application to the patient with cancer. The oncologist, 5(4), 302–311. https://doi.org/10.1634/theoncologist.5-4-302
26

## Slide 27
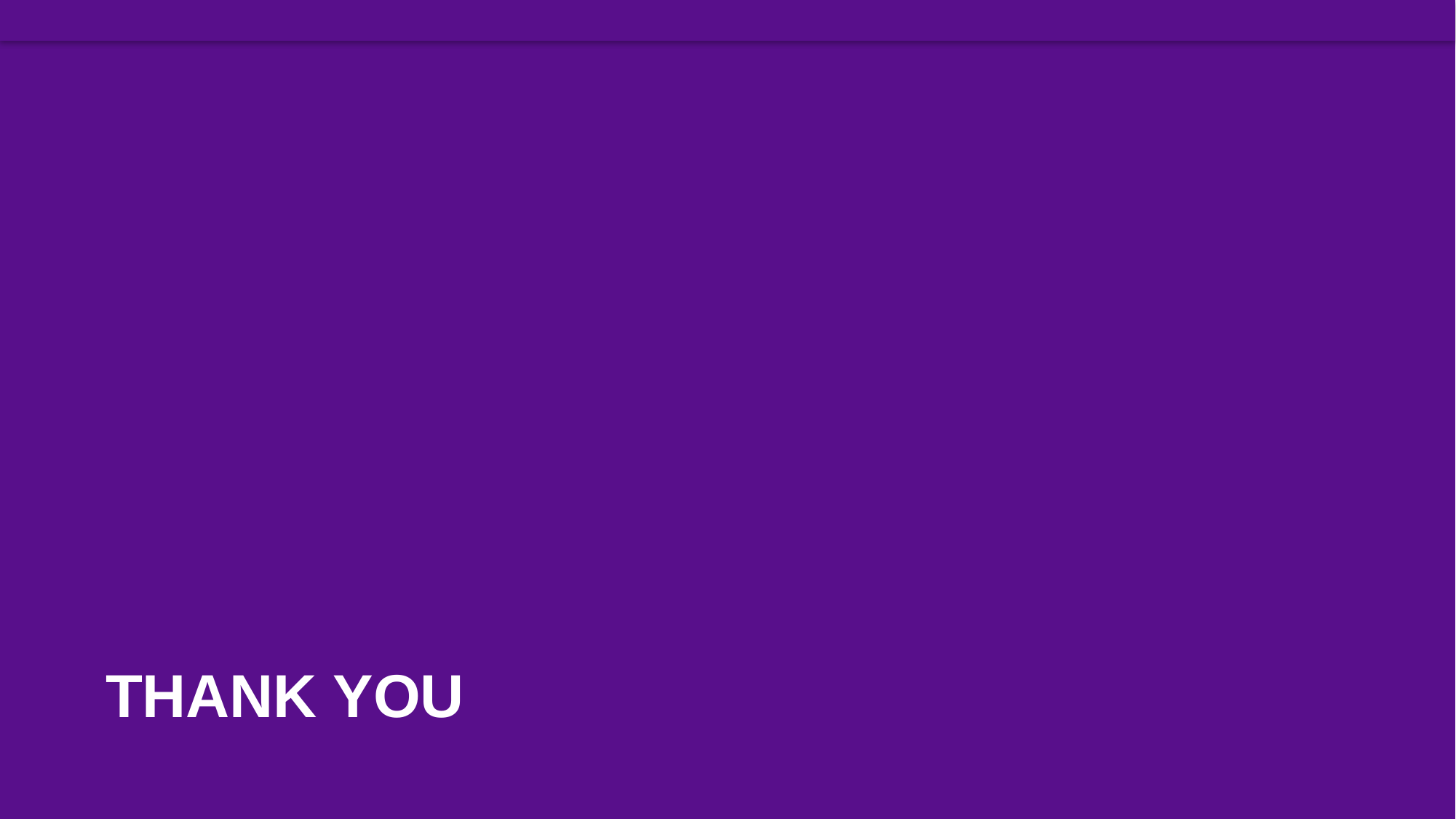

Supplement: Supplementary file 1 — FPF Curriculum.pptxFPF Curriculum Recording.mp4Role-Play Script Without FPF.docxRole-Play Script With FPF.docxFPF Participant Worksheet.docxFPF Instructor Worksheet.docxFPF Survey.docxSP Training.pptxSimulated Participant Training Case.docxFPF-SAT.docx [file mep_2374-8265.11445-s001.zip › H. SP Training.pptx]
