# Supplementary material for: Development and Implementation of a Family Presence Facilitator Curriculum for Interprofessional Use in Pediatric Medical Resuscitations
Source: MedEdPORTAL. 2024 Oct 8;20:11445. doi: 10.15766/mep_2374-8265.11445 (PMC11458738; doi:10.15766/mep_2374-8265.11445)
Supplement: Supplementary file 1 — FPF Curriculum.pptxFPF Curriculum Recording.mp4Role-Play Script Without FPF.docxRole-Play Script With FPF.docxFPF Participant Worksheet.docxFPF Instructor Worksheet.docxFPF Survey.docxSP Training.pptxSimulated Participant Training Case.docxFPF-SAT.docx [file mep_2374-8265.11445-s001.zip › I. Simulated Participant Training Case.docx]

**Appendix I: *MedEdPORTAL* Simulated Participant Case Development Tool**

Date: May 8, 2024

Primary Case Author: Ellen Duncan, MD, PhD

Secondary Case Author: Selin Sagalowsky, MD, MPH

Standardized Patient Educator: Virginia Drda, BA

Name of Case: Infant with Cardiac Arrest due to Ventricular Fibrillation

Name of Educational and/or Assessment Activity: Family Presence Facilitator (FPF) Curriculum – Simulated Participant Training

Patient Name: Erika

Parent Name: All may be used

Chief Complaint: Episode of cyanosis and limpness

Most Likely Diagnosis and Differential with Rationale From History and/or Physical Exam: Cardiovascular Arrest due to Ventricular Fibrillation

Domains: Check all that apply

☒ Professionalism

☒ Communication and Interpersonal Skills

- Medical History
- Physical Exam
- Shared Decision-Making
- Patient Education
- Clinical Reasoning
- Documentation
- Handoff
- Presentation
- Other:

Type and Level of Learner: Learners participating in this in-situ simulation are interdisciplinary, comprising: medical students (0-1); resident physicians (0-2); pediatric emergency medicine fellows (1-6); attending physicians (0-2); nurses of various experience levels (2-3); clinical pharmacists (1-2); and occasionally colleagues including social workers, child life specialists, respiratory therapists, and/or subspecialty providers. The intention is for the composition of the team participating in this simulation exercise to reflect that of a real-life team caring for this patient.

**Case Objectives**: Please list specific objectives for each of the domains checked above.

Learning objectives:

1. Articulate the purpose and benefits of the Family Presence Facilitator (FPF) role
2. Demonstrate the key behaviors associated with the specified personality type(s)
3. Demonstrate escalation and/or de-escalation of behaviors, based on support provided by the FPF

| SETTING: outpatient, inpatient, ED, home, nursing home, rehab, group, etc. | Pediatric Emergency Department |
| --- | --- |
| FAMILY MEMBER PROFILE: You (SP) are one of two parents of Erika, a 6-month-old infant girl who is otherwise healthy but has had a very concerning episode at home of loss of consciousness, as described below. | |
| Age range | 20s-50s |
| Religious/spiritual background | All may be used |
| Sex (e.g., male, female, intersex, transwoman, transman) | All may be used |
| Sexual orientation (e.g., heterosexual, lesbian, gay, bisexual, pansexual, queer, asexual) | All may be used |
| Gender expression (e.g., man, woman, genderqueer) | All may be used |
| Race and ethnicity | All may be used |
| Physical description (e.g., BMI, height range) | All may be used |
| Physical limitations | All may be used |
| Family member appearance (e.g., disheveled, hospital gown, business casual, casual) | Casual, business casual |
| Moulage + location (e.g., none, bruises, scars, body piercing, tattoos) | N/A |
| Affect (e.g., pleasant, cooperative) | Family member should portray one of the three possible affects as described below (anxious, agitated, or quiet) |
| Family group (e.g., who is family, whom they live with) | Infant (manikin) in this case lives with the parent you are portraying, as well as a second parent and 3-year-old brother. The second parent can be of any gender expression, and has stayed home with the 3-year-old. The presenting parent (SP) rode in the ambulance with Erika to the hospital.  Erika and her brother attend daycare three times a week from 9am-1pm. Today she was not in daycare, but home with her family the whole day (it is a weekend). |
| Education | College-educated |
| Level of health literacy | Good health literacy |
| Employment, if any - present and past, noting any current stresses | SP parents works part-time from home as a technology consultant; the second parent works at an advertising firm. |
| Home/homeless - type of dwelling, number of stories, owned or rented | Family lives in a two-bedroom apartment in New York City |
| Financial situation - any current stresses | No current stressors |
| Insurance status (e.g., un/under/insured, public/private, HMO/PPO) | Insured |
| Habits (i.e., diet, exercise, caffeine, smoking, alcohol, drugs) | N/A |
| Activities (i.e., hobbies, sports, clubs, friends) | N/A |
| Typical day - usual daily routine | Three days per week, both parents work while kids go to daycare 9a-1p. Two day per week, the presenting parent stays home with the children. Erika usually naps twice per day (once in the morning and once in the afternoon), and has been eating purees for about 1 month. |

| CASE INFORMATION: |  |
| --- | --- |
| Chief Concern: | Erika is a 6-month-old infant who was sitting in her bouncer chair at home when an episode occurred that caused her parents to be very concerned. Details:   - The whole family was in the kitchen together – SP parent was cleaning dishes and the other parent was feeding Erika’s 3-year-old brother - Erika was sitting in her bouncer chair, about 30 min after consuming pureed foods and formula (pureed pears and rice cereal, which she had had many times before) - Suddenly, she made a gurgling noise and became pale/blue in the face. Her eyes rolled back and her body went limp. - Presenting parent jumped over and grabbed her, patted her back, and tried to breathe into her mouth. Presenting parent’s partner called 911. After a little while (parent is not sure how long – it felt like an eternity but was probably less than 1 minute), Erika regained consciousness and cried - Now she seemed sleepier/weaker than usual - There is no concern that anybody has hurt or harmed Erika. She was with parents and sibling and supervised the entire day |

| CASE INFORMATION: | **Anxious Parent** | **Agitated Parent** | **Quiet Parent** |
| --- | --- | --- | --- |
|  | “She was just sitting in her bouncer and then she turned blue and stopped moving – I thought she was dying!” | “She turned blue and stopped moving, and when we got here the nurse just LEFT US in the room! She almost DIED!” | “She turned blue and limp…It all happened so quickly…” |
| Additional Concerns: | None | | |
| THE PARENT’S STORY: | “Erika was just sitting in her bouncer in the living room, when suddenly, she turned blue and stopped moving! I had no idea what was happening, so I just blew on her face and she seemed to “come to.” It was so scary!” | “I’ve already told multiple people about what happened – why are you asking me again instead of taking care of her?? No one is doing anything, and she almost DIED!” | [Frozen in place, unable to provide more information] |
| PATIENT’S HISTORY OF PRESENT ILLNESS: | | | |
| Onset | Sudden onset of limpness and blue color | | |
| Setting | Erika finished a feed 30 minutes prior to the event | | |
| Duration | 10 seconds | | |
| Time relationships | One-time episode | | |
| Location | N/A | | |
| Radiation | N/A | | |
| Quality | N/A | | |
| Amount | N/A | | |
| Aggravated by what | N/A | | |
| Relieved by what | N/A | | |
| Associated with what | N/A | | |
| Attitude (what does the presenting parent think is the problem, and how do they feel about it) | N/A | | |
| Overall course | N/A | | |
| PATIENT’S REVIEW OF SYSTEMS: | | | |
| No preceding or associated fever  No cough or cold symptoms  No difficulty breathing before this episode  No preceding or associated vomiting or diarrhea  No associated allergic symptoms: no rashes, no swelling of face, lips, or tongue; no associated vomiting | | | |
| Past medical history | Ex-full-term infant, born without complications via vaginal delivery. No complications or infectious during pregnancy. If asked, the mother was negative for Group B Strep bacteria. Infant was discharged home from the nursery with parents. | | |
| Medication allergies (name and reaction) | None | | |
| Environmental allergies (name and reaction) | None | | |
| Illnesses | None | | |
| Vaccinations | Up to date for age | | |
| Surgeries | None | | |
| Accidents/injuries/trauma | None | | |
| Hospitalization | None | | |
| Medications | Vitamin D supplements | | |
| Immunizations | Has received routine pediatric 2-, 4-, and 6-month vaccines | | |
| List any other important social history or information important to this case | Erika was with her family throughout the day and under parental supervision. There is no concern that anybody might have hurt or shaken her. | | |
|  |  | | |
| Family history |  | | |
| Mother, father, siblings, grandparents, and other significant findings | No significant family medical problems. Specifically, if asked, there is no family history of sudden cardiac or unexplained death in childhood. No family history of cardiac rhythm disturbances. | | |
| Physical Exam (**Patient manikin)** - List exam maneuvers expected for this case and any abnormal findings that SP will simulate. (tenderness, hyper-hypo reflex, rebound, weakness, etc.)  At time = 0  HR 150, RR 40, O2 95%, BP 80/40  Pt (manikin) is lying in bed, crying weakly  She is tired but awake  Normal heart rate and blood flow  Lungs clear  Soft abdomen  Moving all extremities  No evidence of trauma  At time = 1 min  HR ventricular fibrillation; RR 0, BP undetectable, O2 undetectable  Pt pulseless and unconscious  No spontaneous cardiac or respiratory activity  Tone is limp  Extremities are dusky/cool  At time = 10 min  HR 150, RR 40, O2 95%, BP 90/40  Crying | | | |
|  | | | |
| PHYSICAL EXAM FINDINGS |  | | |
| 1. Written in layperson’s terms | Parent should know (and state if asked) that patient weighs 6kg | | |
| 1. General appearance - affect, appearance, position of patient at opening (i.e., sitting, lying down, holding abdomen, etc.) | As above (manikin) | | |
| 1. Vital signs | As above (manikin) | | |
| 1. Specific findings and affect | As above (manikin) | | |
| 1. Response to certain physical movements | As above (manikin) | | |
|  |  | | |
| DIAGNOSIS AND DIFFERENTIAL |  | | |
| Diagnosis with support from positive and negative history and PE findings | Cardiopulmonary arrest due to ventricular fibrillation | | |
| Differential with support from positive and negative history and PE findings | Brief resolved unexplained event  Gastroesophageal reflux  Respiratory infection  Seizure  Non-accidental trauma | | |
|  |  | | |
| MANAGEMENT OR DIAGNOSTIC PLAN | At time = 1 min, patient decompensates into cardiopulmonary arrest owing to ventricular fibrillation (see case branch point below), requiring oxygen delivery via bag-valve mask; chest compressions; placement of an intravenous line; and several rounds of electrical shocks and medications. | | |
|  |  | | |
|  | **Anxious Parent** | **Agitated Parent** | **Quiet Parent** |
| CASE BRANCH POINT (At time = 1 min): Patient becomes unconscious, with no detectable pulse or respiratory effort. Medical team commences cardiopulmonary resuscitation (CPR). | “What is HAPPENING?? Oh my gosh, my baby! Is she DEAD??”  “This is just like what happened at home!”  “Oh no, oh no, oh no…”  “CPR – does that mean her heart stopped? Will she feel that shock?”  “Is she going to DIE??” | “What the heck is going on? Why is nobody telling me anything?”  “They have to stop pounding on her chest so hard! They’re hurting her!”  “Do these people even know what to do??” | [Parent is initially stunned and frozen in place]  [If not asked, prompt FPF to bring you a chair, e.g., “I think I need to sit down.”] |
|  |  | | |
| CASE BRANCH POINT (At time = 10 min): Patient is successfully defibrillated, and her pulse and breathing return to normal. | “Oh my gosh, she’s crying! Erika, mommy’s here! Can I see her??” | “Oh wow, she’s crying!” | [Gasp and place hand over mouth in surprise] |
|  |  | | |
| COMMUNICATION CHALLENGES | FPF should provide compassionate support, which can help decrease your anxiety.  When you ask “Is she going to die,” the FPF should provide non-speculative information using the ”Heart-Head-Heart” model (e.g., “I can see that you are worried. The team is now working to bring her heart to a normal rhythm. I am here to support you.” If this is successfully employed, you may dial down your anxiety.  Once the patient is stabilized, the FPF should encourage the Team Leader to provide a summary and next steps. If not spontaneously initiated, you can prompt this (e.g., “What does that mean? Can somebody tell me what happened?”) | FPF should provide compassionate support, which can help decrease your agitation.  If the FPF does not employ de-escalation strategies, you can use angrier body language (balling fists, knocking something to the ground, angry facial expressions) and even try to impede the medical care of your child (e.g., “Stop! You can’t do that to her!”)  Once the patient is stabilized, the FPF should encourage the Team Leader to provide a summary and describe next steps. If not spontaneously initiated, you can prompt, e.g., “What does that mean? Can somebody PLEASE tell me what happened?” | FPF should approach you with an introduction, at which time you can begin to engage and ask appropriate questions (e.g., “What’s going on?”)  Your default should be to appear shocked/stunned but respond well to information (e.g., “Thank you for explaining that.”)  Once the patient is stabilized, the FPF should encourage the Team Leader to provide a summary and describe next steps. The quiet parent need not prompt this but should wait in silence for direction. |
| ANTICIPATED MANAGEMENT MISTAKES | Difficulty providing non-speculative information, especially in the face of repeated prognostication questions. | Difficulty employing de-escalation strategies in the face of increasing agitation. | Difficulty “drawing out” the quiet parent (learners are more likely to delay engaging the quiet parent). |
|  | Difficulty in knowing how much to persist in “persona” role when team performs well: If the FPF is doing a good job in the assigned role, it is okay to dial down your personality and be a cooperative, well-supported parent. That is the goal! | | |
